# Conservation status of ethnic minority medicinal plants in China for the thirty by thirty target

**DOI:** 10.1016/j.isci.2026.116140

**Published:** 2026-06-01

**Authors:** Baocai Han, Jiejing Gao, Changying Xia, Ruyu Yao, Xiaoxia Zhang, Yunfeng Huang, Yude Peng, Tiantian Xue, Chong Luo, Rainer W. Bussmann, Yaodong Qi, Shengxiang Yu

**Affiliations:** 1 State Key Laboratory of Plant Diversity and Specialty Crops, Institute of Botany, Chinese Academy of Sciences, Beijing 100093, China; 2 Key Laboratory of Vegetation and Environmental Change, Institute of Botany, Chinese Academy of Sciences, Beijing 100093, China; 3 University of Chinese Academy of Sciences, Beijing 100049, China; 4 China National Botanical Garden, Beijing 100093, China; 5 School of Life Science, Guizhou Normal University, Guiyang 550025, China; 6 College of Teacher Education, Southwest University, Chongqing, China; 7 Institute of Medicinal Plant Development, Chinese Academy of Medical Sciences and Peking Union Medical College, Beijing 100193, China; 8 Kunming Institute of Botany, Chinese Academy of Sciences, Kunming 650201, China; 9 Guangxi Key Laboratory of Traditional Chinese Medicine Quality Standards, Nanning 530022, China; 10 Guangxi Institute of Chinese Medicine and Pharmaceutical Science, Nanning 530022, China; 11 Guangxi Botanical Garden of Medicinal Plants, Nanning 530023, China; 12 School of Teacher Education, Guizhou Normal University, Guiyang 550025, China; 13 Department of Ethnobotany, Institute of Botany and Bakuriani Alpine Botanical Garden, Ilia State University, Tbilisi 0105, Georgia; 14 Department of Botany, State Museum for Natural History Karlsruhe, 76133 Karlsruhe, Germany

**Keywords:** environmental policy, nature conservation, plant biogeography

## Abstract

Ethnic minority medicinal plants (EMMPs) are an underutilized but critical resource for drug discovery; yet, face escalating threats from overharvesting and climate change. Here, we conduct the first comprehensive assessment of 5,820 EMMPs, evaluating their distribution, conservation status, and climate vulnerability to support global biodiversity goals. Key findings reveal distinct biogeographic patterns among ethnic minority medicinal clades, with southwest China harboring most biodiversity hotspots. Alarmingly, conservation effectiveness declines sharply at higher priority thresholds, leaving many critical habitats unprotected. Meanwhile, climate projections reveal severe range contractions for endemic and threatened EMMPs, even in traditional refugia like the Hengduan Mountains. Our study exposes urgent gaps in EMMP conservation and provides an evidence-based framework to prioritize protection efforts. These insights directly support the implementation of the “30 × 30” initiative, thus offering targeted strategies to safeguard this vital yet neglected component of biodiversity.

## Highlights

• Southwest China harbors the highest diversity of ethnic minority medicinal plants
• Conservation effectiveness declines sharply at higher protection thresholds
• Climate change threatens endemic EMMPs even in traditional refugia
• Priority areas identified support the “30 × 30” conservation target

## Graphical abstract

**Figure:**
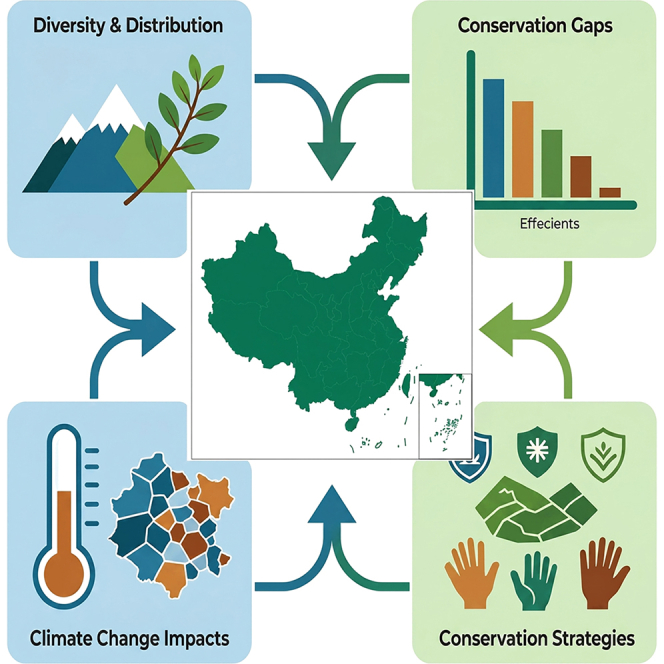

## Introduction

The current global sixth mass extinction event is accelerating due to human activities including significant land use changes and climate disturbances.^1^^,^^2^^,^^3^ Extinction rates are currently exceeding historical background levels by three orders of magnitude,^4^^,^^5^ highlighting an urgent need for coordinated global conservation initiatives. In response, the COP15 framework has set the ambitious “30 × 30” target, which seeks to conserve 30% of terrestrial and marine ecosystems by 2030, anchoring it as a key component of global biodiversity governance.^6^

As a global biodiversity hotspot, China faces urgent conservation challenges, particularly for ecologically and culturally significant species.^7^ Among these, medicinal plants play a vital role in sustaining human health, traditional knowledge, and ecosystem stability.^8^^,^^9^ However, ethnic minority medicinal plants (EMMPs)—distinct from mainstream Han Chinese traditional medicine—are disproportionately understudied despite their critical importance to the livelihoods, cultural heritage, and ecological resilience of indigenous communities.^10^^,^^11^

Unlike Han Chinese traditional medicine, EMMPs represent unique ethnobotanical knowledge systems tied to specific indigenous practices, rituals, and ecological adaptations.^10^^,^^12^ Their conservation is not only a biodiversity priority but also a safeguard against the erosion of irreplaceable cultural heritage.^11^^,^^13^ Alarmingly, over 70% of EMMPs are wild-harvested—a significantly higher proportion than that of mainstream Han medicinal plants.^13^^,^^14^^,^^15^ Compounding this threat, many EMMPs are restricted to fragile ecosystems (e.g., cliff faces, arid deserts, etc.), where even minor climatic shifts or habitat disturbance can trigger population collapse.^16^^,^^17^ This ecological specialization heightens their extinction risk. Of the 603 threatened medicinal plant species in China, EMMPs are disproportionately affected due to narrow distributions, small populations, and dependence on climate-sensitive habitats.^8^

Given the severe threats posed by overharvesting, habitat degradation, and climate change to EMMPs, it is imperative to incorporate their conservation into the “30 × 30” initiative. This integration is crucial not only for safeguarding biodiversity but also for preserving the traditional knowledge systems that are intricately linked to these botanical resources. The lack of detailed research on EMMPs is concerning as it restricts our understanding of their ecological roles and the potential erosion of invaluable medicinal knowledge, a situation with grave implications for biodiversity and human health.

To comprehensively address these critical gaps, we have integrated a robust analytical framework that harnesses large-scale occurrence data alongside multiple indices. This framework includes metrics such as species complementarity (SC) referring to the algorithms which define biodiversity hotspots by selecting the minimum number of areas that could cover all the species,^8^^,^^18^ and weighted endemism, which assigns high weights to species with small ranges and smaller weights to widespread species.^19^^,^^20^^,^^21^^,^^22^ Furthermore, we have employed advanced metrics like phylogenetic diversity (PD) and phylogenetic endemism (PE). PE computes the phylogenetic equivalent of species endemism, assessing the total phylogenetic branch length found within a specific area, adjusted by the global range size of its descendant clade.^23^^,^^24^ To explore how species are distributed under environmental changes, we adopted the species distribution model, which refers to the analysis that combines species distribution records with environmental variables, aiming at predicting the potential distribution range of species.^25^^,^^26^^,^^27^ These indices and models are now increasingly heralded as vital tools in biodiversity conservation, offering deep evolutionary insights that are pivotal for setting conservation priorities.

Incorporating these complex metrics with cutting-edge algorithms of species distribution modeling and ecological niche modeling, our research methodically maps the extant habitats of EMMPs and also projects future habitat shifts under diverse climate scenarios. This dual approach not only details current landscapes but also anticipates the adaptability and resilience needed in forthcoming conservation strategies. The driving force of our study is to lay down a strategic framework aimed at achieving numerous goals: (1) to delineate the distribution patterns and pinpoint the biodiversity hotspots of EMMPs in China; (2) to scrutinize the efficacy of current conservation measures and identify regions where protection is deficient; (3) to gauge the impacts of climatic alterations on the distribution and population health of endemic and imperiled EMMPs; and (4) to forge a comprehensive strategy that aligns the preservation of EMMPs with the ambitious “30 × 30” global biodiversity conservation targets. Through this synthesized and multi-faceted approach, we aim to provide a roadmap that not only ensures the survival of these vital plant species but also fortifies the ecological and cultural landscapes in which they thrive.

## Results

### Diversity and clustering of EMMPs

**Figure 1:**
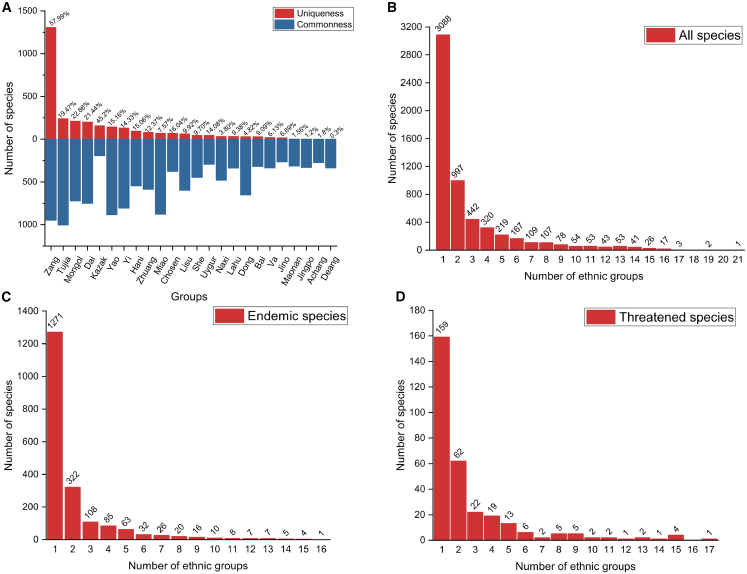
Statistics of species composition and species property (A) Species composition of common species and unique species of 24 ethnic minorities. (B) Species frequency distributions of all EMMPs. (C) Species frequency distributions of endemic EMMPs. (D) Species frequency distributions of threatened EMMPs.

Among EMMPs, notable differences in species richness were observed: Zang medicine (2,259 species), Tujia medicine (1,248 species), and Yao medicine (1,031 species) had the highest EMMP diversity (Table S1). Uniqueness analysis indicated that Zang medicine had the greatest number of unique species (1,310 species), followed by Tujia medicine (243 species) and Mongol medicine (214 species) (Figure 1A). Frequency distributions showed that 3,088 EMMPs were used by just one ethnic group, 997 species by two ethnic groups, and 442 species by three ethnic groups (Figure 1B). For endemic EMMPs, 322 species were used by two ethnic groups, 108 are shared by three ethnic groups, and just one ethnic group used 1,271 endemic EMMPs (Figure 1C). Among threatened EMMPs, 159, 62, and 22 species were used by one, two, and three ethnic groups respectively (Figure 1D).

**Figure 2:**
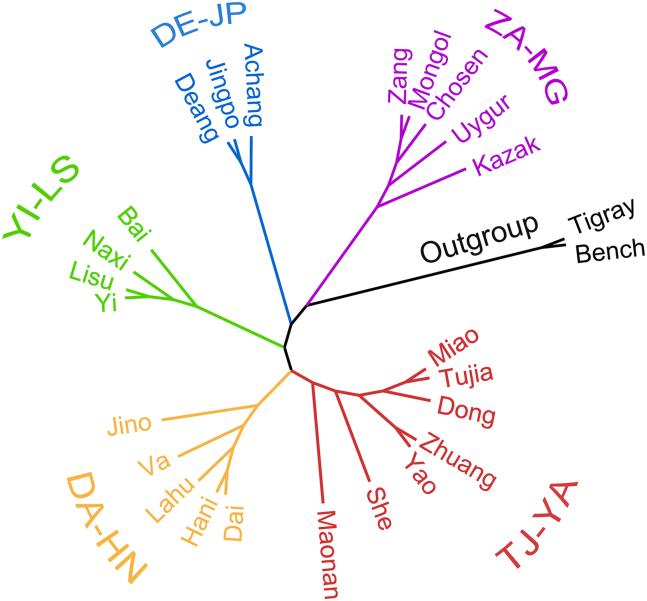
Cluster analysis of all EMMPs of 24 ethnic minorities

Cluster analysis classified EMMPs from 24 ethnic minorities into five clades (Figures 2 and S3A), with the ZA-MG clade diverged first, followed sequentially by the DE-JP, YI-LS, DE-JP, and TJ-YA clades. Shared EMMPs within each clade included 49 species across all seven ethnic groups in the TJ-YA clade (Figure S3B), 61 across all five ethnic groups in the DA-HN clade (Figure S3C), 61 across all four ethnic groups in the YI-LS clade (Figure S3D), 196 across all three ethnic groups in the DE-JP clade (Figure S3E), and 13 across all five ethnic groups in the ZA-MG clade (Figure S3F).

### Geographical distribution pattern and hotspots of EMMPs

The grids with higher EMMP SR were primarily distributed in southwest China, including in the Hengduan Mountains, Qinling Mountains, Bashan-Wushan Mountains, Nanling Mountains, and Wuling Mountains, (Figure S4A). Higher EMMP SR regions for the five clades were mainly distributed in southwest China, such as the Hengduan Mountains and surrounding areas (Figures S4B–S4F). Correlation analysis showed a weak correlation between the ZA-MG clade’s distribution and other clades, with the weakest with DA-HN clade (r = 0.58, *p* < 0.01). The distribution patterns of the DE-JP and DA-HN clades showed a strong correlation (r = 0.98, *p* < 0.01) (Figure S5). Endemic EMMPs were largely distributed in the Hengduan Mountains, northern Yunnan, the Bashan-Wushan Mountains, and the Nanling Mountains (Figure S6). In contrast, the distribution area of threatened EMMPs with higher SR was focused on southern China, especially the Hengduan Mountains, southern and southeastern Yunnan, southwestern Guangxi, and northern Guangxi (Figure S7). SC algorithms results showed high-richness grids were mainly in southwest China (Figures S8–S10), consistent with the distribution patterns of the WE algorithm, PD, and PE (Figures S11–S15). Top 5% threshold for SR, SC, WE, and PA were mostly confined to southwest China (Figure S16).

**Figure 3:**
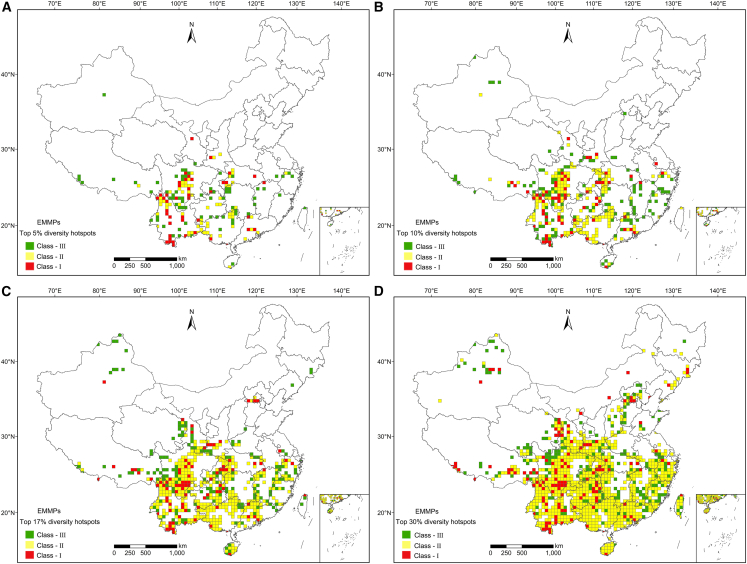
Spatial distribution patterns of final diversity hotspots for all EMMPs at thresholds of top 5% (A), top 10% (B), top17% (C), and top 30% (D)

Across all thresholds, most final EMMP hotspots were located in southwestern China (Figure 3). The top 5% hotspots included 179 grids, contained 97.11% of all species, 97.08% of endemic species, 96.73% of threatened species, and 97.31% of protected species (Tables S4 and S5), and were mainly distributed in the northern and southern Hengduan Mountains, southern and southeastern Yunnan, and the Wushan Mountains (Figure 3A). Among these hotspot grids, 42 were class-I hotspots, which were identified as hotspots by all four algorithms and primarily distributed in the mountainous regions of southwestern China. The top 10% hotspots (358 grids) covered 98.44% of all species, 98.84% of endemic species, 98.04% of threatened species, and 99.1% of protected species (Tables S4 and S5), adding southeast Xizang, the Qinling Mountains, the Wuling Mountains, and northern Yunnan to the 5% hotspots (Figures 3A and 3B). The top 17% hotspots (609 grids) contained 99.43% of all species, 99.60% of endemic species, 99.67% of threatened species, and 100% of protected species (Tables S4 and S5), with Qilian Mountains, Tianmu Mountains, and northern Taihang Mountains added (Figures 3A–3C). The top 30% hotspots (1,075 grids) covered 99.9% of all species, 100% of endemic species, threatened species, and protected species (Figure 3D, Tables S4 and S5), incorporating three new regions, namely the Tianshan Mountains, Changbai Mountains, and central Himalayas (Figures 3A–3D).

Hotspots of the TJ-YA clade were dispersed with increasing threshold values throughout the southern Hengduan Mountains, southern and southeastern Yunnan, Wushan Mountains, south-central Guangdong, Nanling Mountains, Tianmu Mountains, and Taihang Mountains (Figure S17). Hotspots of the DA-HN clade were found distributed primarily in Yunnan, including the southern Hengduan Mountains and southern Yunnan (Figure S18). Hotspots of the YI-LS clade were distributed in the southern Hengduan Mountains, the intersection of Sichuan and Yunnan, and southern Yunnan (Figure S19). Hotspots of the DE-JP clade were confined to the northern Hengduan Mountains, and these hotspot areas grew as the threshold rose (Figure S20). The Qilian and Qinling Mountains, the northern Hengduan Mountains, northwest Yunnan, and southeast Xizang harbor distinctive hotspots of the ZA-MG clade (Figure S21).

### EMMP conservation effectiveness and gaps

**Figure 4:**
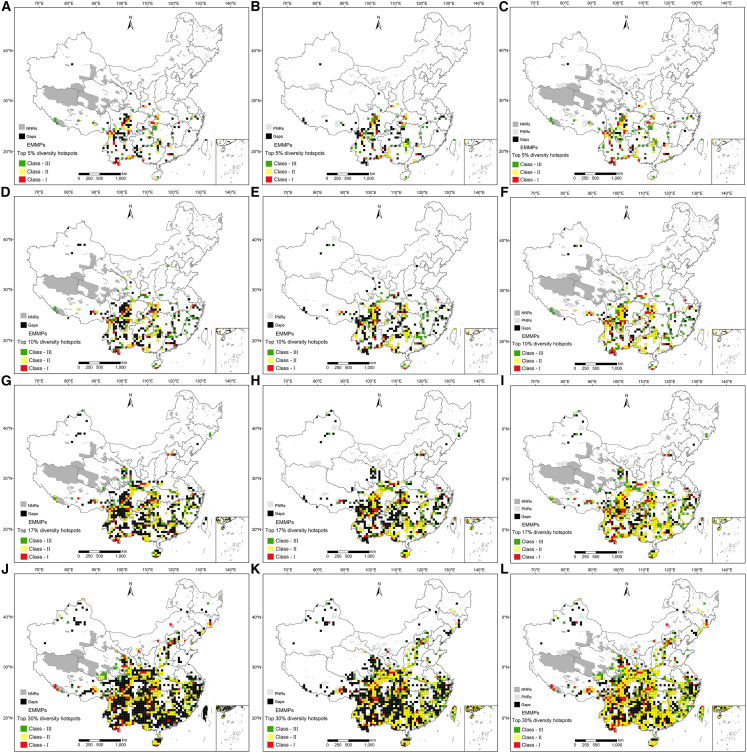
Spatial distribution patterns of conservation effectiveness and gaps for all EMMPs based on four different thresholds Based on top 5% (A–C), top 10% (D–F), top 17% (G–I), and top 30% (J–L) threshold, conservation effectiveness and gaps for NNRs, PNRs, and NNRs-PNRs, respectively.

Conservation effectiveness analysis revealed that as the hotspot threshold increased, the overall conservation effectiveness of EMMPs decreased, while the scope of conservation gaps expanded progressively (Figure 4).

At the top 5% threshold, for national nature reserves (NNRs), the protection rate of hotspot grids was 56.42% (101/179), with effective conservation rates of 90.98% (5,295 species) for all species, 94.16% (1,869 species) for endemic species, 90.2% (276 species) for threatened species, and 89.24% (199 species) for protected species. For NNRs-provincial nature reserves (PNRs) combinations, the conservation effectiveness of hotspot grids reached 82.12% (147/179), and the corresponding conservation rates increased to 93.06% (5,416 species), 95.62% (1,898 species), 92.81% (284 species), and 91.93% (205 species) respectively. Conservation gaps included 78 grids unprotected by NNRs, 83 by PNRs, and 32 by neither, mainly distributed in the northern Hengduan Mountains, Guizhou-Guangxi junction, and central Guangdong (Figures 4A–4C) . These gaps contained 87.04% (5,066 species) of all species, 83.32% (1,654 species) of endemic species, 78.1% (239 species) of threatened species, and 84.3% (188 species) of protected species.

At the top 10% threshold, NNRs’ protection rate of hotspot grids decreased to 54.75% (196/358), and NNRs-PNRs combinations achieved 81.28% (291/358) conservation effectiveness. Conservation gaps involved 162 grids unprotected by NNRs, 168 by PNRs, and 67 by neither, scattered in the Hengduan Mountains, Wushan Mountains, and Guizhou (Figures 4D–4F). These gaps contained 90.36% (5,259 species) of all species, 87.71% (1,741 species) of endemic species, 84.31% (258 species) of threatened species, and 89.24% (199 species) of protected species.

At the top 17% threshold, NNRs’ protection rate further declined to 49.26% (300/609), with PNRs outperforming NNRs in conservation effectiveness. NNRs-PNRs combinations had 75.21% (458/609) conservation effectiveness for hotspot grids. Conservation gaps included 309 grids unprotected by NNRs, 301 by PNRs, and 151 by neither, mostly located in northern Xinjiang, Tianshan, Altai Mountains, Qilian Mountains, Hengduan Mountains, central-northern Yunnan, central-southern Guizhou, southern Jiangxi, and Tianmu Mountains (Figures 4G–4I). These gaps contained 94.62% (5,507 species) of all species, 92.8% (1,842 species) of endemic species, 89.87% (275 species) of threatened species, and 94.62% (211 species) of protected species.

At the top 30% threshold, NNRs’ protection rate dropped to 41.58% (447/1,075), and NNRs-PNRs combinations decreased to 68.93% (741/1,075) conservation effectiveness. Conservation gaps expanded significantly, with 628 grids unprotected by NNRs, 559 by PNRs, and 334 by neither, concentrated in Altai Mountains, Tianshan Mountains, Qilian Mountains, Hengduan Mountains, southern Sichuan, and central-northern Yunnan (Figures 4J–4L). These gaps included 96.89% (5,639 species) of all species, 95.16% (1,889 species) of endemic species, 91.18% (279 species) of threatened species, and 96.86% (216 species) of protected species.

**Figure 5:**
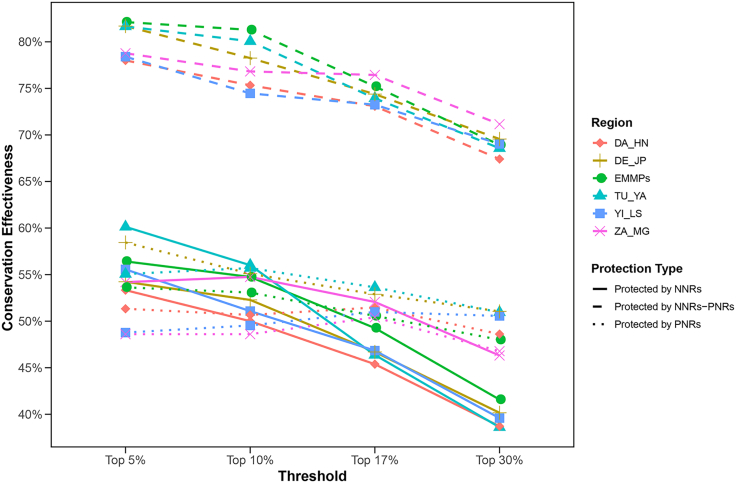
Statistics of conservation effectiveness of all EMMPs of 24 ethnic minorities (EMMPs), all EMMPs of TJ-YA clade (TJ-YA), all EMMPs of DA-HN clade (DA-HN), all EMMPs of YI-LS clade (YI-LS), all EMMPs of DE-JP clade (DE-JP), and all EMMPs of ZA-MG clade (ZA-MG)

For the five EMMP clades, conservation effectiveness showed a gradual declining trend with increasing thresholds (Figure 5). In NNRs, the TJ-YA clade performed best at top 5%, and top 10%; ZA-MG clade at top 17% and top 30%, while DA-HN clade ranked lowest at top 5%, top 10% and 17%; and TJ-YA clade at top 30%. In PNRs, DE-JP clade excelled at top 5% and 30%; TJ-YA clade at top 10% and 17%; ZA-MG clade was the lowest. For NNRs-PNRs combinations, ZA-MG clade (top 17%, top 30%) and all EMMPs (top 5%/, top 10%) had the highest effectiveness, while DA-HN clade (top 5%, top 17%, top 30%) and YI-LS clade (top 10%) were the lowest. The overall conservation status varied among clades (Figures 5 and S23–S27, and Table S4).

### Impacts of climate change on EMMPs

Prediction analysis indicated 2,132 (99.72%) endemic and threatened species had TSS ≥0.5 and AUC ≥0.7 (Table S2). Prediction analysis also showed that 49.62% (representative concentration pathway [RCP] 2.6), 51.74% (RCP 4.5), 52.63% (RCP 6.0) and 53.19% (RCP 8.5) of EMMPs would experience increases in their potential distribution areas under various climate change scenarios (Figure S28 and Table S6). The potential distribution areas of roughly 50.28% (RCP 2.6), 48.26% (RCP 4.5), 47.37% (RCP 6.0), and 46.76% (RCP 8.5) of species will shrink (Figure S28 and Table S6), while the potential distribution areas of a small number of species will remain unaltered (Table S6). The SR of endemic and threatened EMMPs is higher in southern China under the current climate and four future climate change scenarios, particularly in the Himalayas, southeastern Xizang, and the Hengduan Mountains (Figures S29–S33). The potential distribution areas of the DA-HN, YI-LS, DE-JP, and ZA-MG clades are generally congruent in spatial terms, covering the Himalayas, southeast Xiang, the Hengduan Mountains, Yunnan, and Taiwan (Figures S29–S33). In contrast to the other clades, the primary potential distribution areas of the TJ-YA clade under the current and future climate change scenarios are spread throughout central, southern, and southeastern China (Figures S29–S33).

### Predicted EMMP SR distribution patterns and their shifts

**Figure 6:**
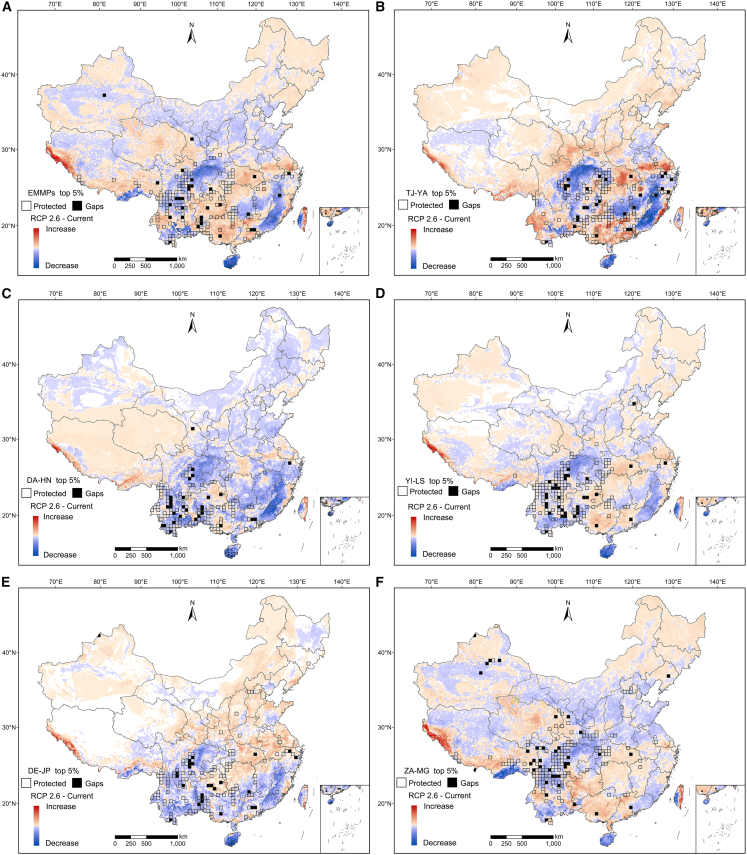
Species richness change for different plant categories by 2070 under the emission scenarios of representative concentration pathway (RCP) 2.6. The white and black grids represent the top 5% hotspot grids covered or uncovered by national and provincial nature reserves, respectively (A) Endemic and threatened EMMPs of 24 ethnic minorities. (B) Endemic and threatened EMMPs of TJ-YA clade. (C) Endemic and threatened EMMPs of DA-HN clade. (D) Endemic and threatened EMMPs of YI-LS clade. (E) Endemic and threatened EMMPs of DE-JP clade. (F) Endemic and threatened EMMPs of ZA-MG clade.

Predicted SR in southwestern and southeastern China varied significantly under different future climate scenarios. For example, predicted EMMP SR increased in 57.99% (RCP 2.6), 56.54% (RCP 4.5), 56.83% (RCP 6.0), and 55.39% (RCP 8.5) of grids (Table S7), which were mainly distributed in the eastern and western Himalayas, Central China, central and southern Yunnan, western and southern Guangxi, and central and western Guangdong (Figures 6 and S34–S36). However, southwest China, especially the Hengduan Mountains, is predicted to experience a major decrease in SR (Figures 6 and S34–S36), and will also experience significantly decreases in the predicted SR of all five clades under all scenarios (Figures 6 and S34–S36). With the top 5% hotspot threshold, only a small number of these hotspot grids were included, most of which are effectively conserved in NNRs and PNRs (Figure 6). However, 37.64% (RCP 2.6), 38.73% (RCP 4.5), 38.84% (RCP 6.0), and 40.48% (RCP 8.5) of grids will face SR decreases (Table S7), primarily distributed in the Bashan region of central China, the northern Hengduan Mountains, southeastern Tibet, northern Guangxi, eastern and southeastern regions of China, and Hainan Island (Figures 6 and S34–S36). These regions, like the northern Hengduan Mountains, stood out as the primary hotspots for EMMPs with conservation gaps. Furthermore, in regions where richness was rising, few or no gaps were found in the distribution of hotspot grids under the top 5% hotspot threshold, although conservation gaps persisted in regions where SR was falling, such as the Hengduan Mountains (Figures 6 and S34–S36). For other hotspot thresholds, conservation gaps expand alongside increases in hotspot grid numbers.

## Discussion

Based on a dataset of over 900,000 geo-referenced occurrence points, we have constructed a comprehensive picture of EMMPs in terms of diversity, distribution, and conservation by employing multiple diversity algorithms. This study sheds new light on the relationships among EMMPs used by different ethnic minorities, the current conservation status of EMMPs and potential challenges posed by climate change. Therefore, the findings of this study will support the priority management of EMMP conservation and sustainability, and will be helpful in realizing the conservation target of the “30 × 30” target.

### Composition and distribution characteristics of EMMPs

EMMPs comprise large percentages of Chinese traditional medicinal plants (66%), protected species (21.6%), and threatened species (7.88%).^9^^,^^28^^,^^29^ While the Zang and Tujia ethnic groups utilized the largest number of EMMP species, our findings highlight distinct differences in the species composition of EMMPs used by different ethnic minorities. On one hand, certain groups exhibited remarkable uniqueness in their medicinal plant usage. For instance, the EMMPs of Dai, Zang, Kazak, and Mongol showed the highest proportions of unique species (Figure 1). This suggests that these groups have preserved unique medicinal traditions tied to EMMP usage, and provides valuable insights into the historical development and cultural diversity of medicinal plant discovery. On the other hand, our study also uncovered strong evidence of cultural blending through the cross-use of medicinal materials. A significant proportion of EMMPs (46.94%) were shared among multiple ethnic groups, with *Acorus gramineus*, for example, being employed by 21 different groups (Table S1). This widespread sharing of medicinal resources demonstrates their interchangeability in regions where ethnic communities coexist or interact, such as cross- or mixed-habitation areas.^30^ This interplay between uniqueness and shared usage underscores the complex cultural and ecological relationships shaping EMMP utilization across ethnic minorities.

The hotspots of all EMMPs were mainly located in the mountains of southwestern China, including the Hengduan Mountains, Bashan-Wushan Mountains, and Nanling Mountains (Figure S4), which is congruent with the distribution patterns of Chinese medicinal plants, seed plants, and threatened plants.^9^^,^^28^^,^^31^ Furthermore, by examining the hotspots of each of the five EMMP clades, we found most hotspots on the clade level were confined to southwest China (Figures 3 and S17–S21). This pattern showed strong similarity to the distribution patterns of Chinese endemic woody plants and national key protected wild plants.^32^^,^^33^^,^^34^

### Suggestions and strategies for conservation networks focused on EMMPs

Our analysis indicated a sharp decline in the conservation effectiveness of NNRs and PNRs as the hotspot threshold increased. At a 30% threshold, combined NNR and PNR coverage protected less than 70% of hotspots (Figure 5 and Table S4). Notably, under the top 5% threshold, only 82.12% of EMMP hotspots (147/179) were adequately protected, a lower effectiveness than reported by Xia et al.^9^ for Chinese medicinal plants (83.33% of 150 hotspot grids), despite our smaller sample size (5,820 EMMPs vs. 9,756 medicinal plants). Furthermore, EMMP conservation effectiveness lagged behind that of Chinese seed plants (90.53%) and threatened medicinal plants (85.7%).^8^^,^^31^ These findings underscore the inadequacy of current protected area networks (NNRs and PNRs) for safeguarding EMMPs. To meet China’s biodiversity conservation targets, including the “30 × 30” goal, prioritizing the protection of EMMP diversity hotspots is imperative.

The present study further revealed distinct disparities in EMMP conservation between NNRs and PNRs. Despite the large numbers of numerous hotspots within NNRs, their conservation efficacy for EMMPs was lower than that of PNRs (Figure 5 and Table S4). Although NNRs cover a substantially larger area than PNRs (941,700 vs. 331,600 km^2^) and serve important conservation functions, NNRs are less effective than PNRs for EMMP conservation. A reasonable explanation for this divergence is that PNRs have a numerical advantage (806 PNRs vs. 464 NNRs), which contributes to their high conservation effectiveness for EMMPs. Another possible contributing factor is that PNRs are distributed more closer to populated areas, which might have led to their coincidental coverage of EMMP hotspots. This study also indicated that limited hotspots were present in some large NNRs in western China (Figures 3 and S17–S21), which may indicate that the area of nature reserves limits conservation efforts in regions with low biodiversity. To address these problems, a serious effort to protect biodiversity hotspots is recommended, including establishing new nature reserves,^33^ optimizing the layout of nature reserves,^9^ *in situ* or *near-situ* conservation projects,^35^^,^^36^^,^^37^ and community co-construction and co-management endeavors.^17^ In addition, conservation effectiveness can be increased by considering different types of protected habitats, including wild nurseries and botanical gardens as *ex situ* conservation area.^38^^,^^39^ Therefore, focusing on EMMPs with particular properties, we recommend targeted conservation strategies for EMMPs in the future based on the establishment of plant micro-reserves with the benefits of low cost, quick construction, immediate effects, and regional coverage.

With regard to the conservation situation of individual EMMP clades, evident discrepancies in conservation effectiveness were observed. When the hotspot threshold was set to the top 5% or top 10%, the DA-HN, YI-LS, and ZA-MG had much lower conservation effectiveness than other clades (Figure 5 and Table S4). For the ZA-MG clade, the conservation effectiveness of NNRs under the top 5%, top 10%, and top 30% thresholds was equal to the effectiveness of PNRs (Figure 5 and Table S4). Our results also indicate that, even within a single clade, EMMP conservation effectiveness can exhibit variability. Therefore, in evaluations of conservation effectiveness, EMMP taxa have received limited targeted conservation efforts. Given that these taxa represent a significant portion of Chinese medicinal plants,^11^^,^^40^ already have been the source of many new drug discoveries,^41^^,^^42^^,^^43^^,^^44^ and have numerous cultural applications,^45^^,^^46^ we propose that future research and conservation efforts should be expanded to develop these plant resources with significant medicinal value and thus safeguard human health.

### Challenges to EMMP conservation posed by climate change

Based on our prediction of potential suitable habitat areas under multiple climate change scenarios, the potential distributions of endemic and threatened EMMPs are predicted to vary significantly across different climate change scenarios, indicating that climate change will substantially alter their distribution ranges. Our result of potential distributions showed that SR will decrease in southwestern China, especially in the Hengduan Mountains and Yunnan, under three scenarios, although increasing under the RCP 4.5 scenario (Figures 6 and S34–S36). Notably, the predicted SR of the ZA-MG clade will decrease sharply in its hotspots of the Hengduan Mountains and surrounding areas under the same three climate change scenarios, posing a great challenge to EMMPs confined to northern China (Figures 6 and S34–S36). In spatial terms, many biodiversity hotspots reported previously, such as the Hengduan Mountains, Nanling Mountains, and most of eastern China, will confront a high risk of decreases in SR,^20^^,^^28^^,^^29^^,^^30^ despite being predicted to have high SR under various future climate change scenarios (Figures 6 and S34–S36). Decreased SR will lead to a considerable risk of species loss from nature reserves, posing a major challenge to existing static conservation networks.^47^^,^^48^^,^^49^

In addition, we also found many EMMPs are projected to expand their distribution ranges under future climate change scenarios (Figures 6 and S34–S36, Tables S6 and S7). For example, the predicted SR of the TJ-YA clade will increase in central China under the RCP 4.5, RCP 6.0, and RCP 8.5 scenarios, but this region, particularly the Sichuan Basin, is predominantly characterized by human-disturbed land. Therefore, this change will make little contribution to the expansion of EMMP distribution ranges, as habitat fragmentation and human activities will offset this alteration.^50^^,^^51^^,^^52^^,^^53^

In addition, this study also indicated that concomitant with the increase in the hotspot threshold, greater numbers of conservation gaps were detected in these high-biodiversity areas. Therefore, fully considering potential changes in species distributions, conservation effectiveness, and gaps caused by climate change, as well as proper optimization of the layout of the protection network, will be necessary to effectively protect Chinese EMMPs and achieve the goals of the Post-2020 Global Biodiversity Framework and the “30 × 30” target.^54^

This study identified key geographical regions that should be prioritized for inclusion in the future 30% protected area system. These include Hengduan Mountains, and areas with overlapping protection gaps between PNRs and NNRs—regions characterized by high EMMP species richness but insufficient existing protection coverage. Prioritizing the protection of these areas can rapidly improve the protection coverage of EMMPs, a key biological group, aligning with the core requirement of the “30 × 30” target. Furthermore, addressing the mismatch between area advantage and conservation effectiveness observed in existing NNRs and PNRs for EMMP protection, the spatial optimization framework for protected areas based on species distribution and conservation effectiveness proposed in this study can guide the refined layout of future “30 × 30” protected area planning. This involves strengthening protection linkage in regions with dense PNRs distribution, supplementing small protected area patches in NNR-deficient areas, and constructing a composite protection network consisting of core protected areas, buffer linkage zones and ecological corridors to fill the structural gaps in the current protection system. finally, the identified differences in conservation efficacy of EMMPs between different types of protected areas (NNRs/PNRs) and their driving factors (e.g., quantity, geographical location) provide a scientific basis for hierarchical management and resource allocation during the advancement of the “30 × 30” target. For instance, in EMMP hotspots adjacent to populated areas or cultural hubs, the geographical advantage of PNRs can be leveraged to strengthen *in-situ* conservation and community co-management, enhancing the feasibility and sustainability of protection measures. In summary, through the precise identification of critical protected areas, the construction of optimized pathways for the protection system, and the adaptation of management strategies, this study provides targeted technical support and decision-making references for the scientific planning and efficient implementation of the “30 × 30” protected area system.

### Limitations of the study

Despite the comprehensive nature of this study, several limitations should be acknowledged. First, the distribution data of EMMPs were primarily derived from specimen records, which may inherently contain spatial biases and gaps in sampling intensity, particularly in remote or under-collected regions. Second, our conservation effectiveness assessment focused on national and provincial nature reserves due to data availability, potentially overlooking other effective area-based conservation measures that also contribute to EMMP protection. Finally, the cultural dimensions of EMMP use, such as the specific knowledge systems and traditional practices, were not directly quantified, despite their importance for holistic conservation strategies.

## Resource availability

### Lead contact

Further information and requests for resources and reagents should be directed to and will be fulfilled by the lead contact, Shengxiang Yu (yushengxiang@ibcas.ac.cn).

### Materials availability

This study did not generate new unique reagents or materials.

### Data and code availability

• All data reported in this study will be shared by the lead contact upon request.
• This study does not report original code.
• Any additional information required to reanalyze the data reported in this study is available from the lead contact upon request.

### Availability of data and materials

Distribution data are available through the Chinese Virtual Herbarium (http://www.cvh.ac.cn/). Other data that support the findings of this study are available in the supplemental information of this article.

## Acknowledgments

We thank the Chinese Virtual Herbarium (CVH) for permission to access species distribution data. This study was funded by the 10.13039/501100012166National Key Research and Development Program of China (2024YFF1307602) and the 10.13039/501100001809National Natural Science Foundation of China (32071654).

## Author contributions

B.H.: conceptualization (equal), methodology (equal), and writing – review and editing (equal); J.G.: data curation (equal), formal analysis (lead), investigation (supporting), methodology (lead), writing – original draft (lead), and writing – review and editing (equal); C.X.: formal analysis (equal), investigation (supporting), methodology (equal), writing – original draft (equal), and writing – review and editing (equal); R.Y.: investigation (supporting), writing – original draft (equal), and writing – review and editing (equal); X.Z.: conceptualization (equal) and formal analysis (supporting); Y.H.: conceptualization (equal) and data curation (supporting); Y.P.: formal analysis (equal) and investigation (supporting); T.X.: data curation (equal), formal analysis (supporting), and investigation (supporting); C.L.: conceptualization (equal), investigation (equal), project administration (equal), supervision (equal), and writing – review and editing (equal); R.W.B.: conceptualization (equal), formal analysis (equal), project administration (equal), and writing – review and editing (equal); Y.Q.: conceptualization (lead), data curation (equal), formal analysis (equal), investigation (equal), project administration (equal), supervision (equal), writing – review and editing (equal); S.Y.: conceptualization (lead), data curation (lead), formal analysis (equal), funding acquisition (lead), investigation (lead), methodology (equal), project administration (lead), supervision (lead), and writing – review and editing (lead).

## Declaration of interests

The authors declare that they have no competing interests.

## STAR★Methods

### Key resources table

**Table:** 

REAGENT or RESOURCE	SOURCE	IDENTIFIER
**Deposited data**		

Ethnic minority medicinal plants (EMMPs) occurrence data	This study	http://www.cvh.ac.cn
EMMPs taxonomic data	This study	http://www.sp2000.org.cn/
Plant occurrence native range data	This study	https://powo.science.kew.org/
China nature reserves geo-document layers	This study	http://www.mee.gov.cn; https://www.protectedplanet.net/

**Software and algorithms**		

ArcGIS (Version 10.6)	Esri	https://www.esri.com/en-us/arcgis/products/arcgis-pro/overview
R (Version 4.2.2)	R Core Team	https://cran.r-project.org/bin/windows/base/old/4.2.2/
ClustalW (Version 2.1)	Thompson et al.^55^	https://bioweb.pasteur.fr/packages/pack@ClustalW@2.1
MrBayes (Version 3.2.0)	Ronquist et al.^56^	https://nbisweden.github.io/MrBayes/download.html

### Experimental model and study participant details

This study does not include experimental model or study participant.

### Method details

#### EMMP list and geographic distribution information

The most comprehensive EMMP list was compiled based on the Dictionary of Chinese Ethnic Medicine, which contains all pharmaceuticals (medical materials) that ethnic minorities in China currently utilize.^12^ In total, 7,022 plant-based medications associated with 55 ethnic minorities have been recorded. Subsequently, We updated the EMMP list using the “Catalogue of Life China 2023 Annual Checklist” (http://www.sp2000.org.cn/), replacing synonyms with their accepted names, while retaining all infraspecific taxa, such as subspecies and varieties. Non-native and cultivated species were excluded.^57^ After synonym standardization, a total of 6,070 species were retained. Among these, 250 species were not recognized as stable medicinal resources. To eliminate potential biases associated with the inclusion of species characterized by ambiguous or inconsistent usage records, these 250 species were excluded from subsequent analyses. After a careful assessment based on these criteria, 24 ethnic groups were confirmed to satisfy the selection benchmarks, and most of them are distributed in Southwest China (Figure S1).

EMMP occurrences were primarily source from the Chinese Virtual Herbarium (http://www.cvh.ac.cn) specimen records, excluding those meeting any of the following criteria: (1) lacking detailed locality information (occurrence records with accurate locality information were geo-referenced according to Chinese gazetteers); (2) identical latitude and longitude; (3) zero and/or integer latitude and longitude; (4) coordinates falling within sea; (5) coordinates outside the species’ native range recorded in POWO (https://powo.science.kew.org/). A database containing 5,820 species belonging to 1,546 genera and 245 families with 912,163 occurrence points was constructed to analyze the EMMP distribution patterns of 24 ethnic minority groups (Table S1). The occurrence database contained 1,985 endemic species and 306 threatened species in China, including 24 critically endangered species, 94 endangered species, and 188 vulnerable species (Table S1). endemic species were defined followed Anderson et al. (1994),^58^ and threatened EMMPs information followed Qin et al. (2020).^59^

#### Statistical analysis of species properties and cluster analysis

To explore the species composition of different ethnic groups, we classified EMMPs based on their frequency of occurrence. In this analysis, EMMPs used by more than one ethnic group were considered common species, and those by only one were considered unique species. This analysis was conducted for all species, endemic species, and threatened species. After classifying the species, we performed EMMP cluster analysis to explore the relationships among different ethnic groups’ utilization of these plants. For clustering , we coded each ethnic minority utilizing a species as 1; otherwise, we coded that minority–species combination as 0. In total, 24 EMMP matrix codes were acquired, and these codes were then translated into coding sequences (1 = A, 0 = C). Outgroups were selected, including medicinal plants from the Bench (Bench-Maji Zone) and Tigray (Kilte Awulaelo District, Tigray Region) ethnic groups in Ethiopia,^60^^,^^61^ and matrix coding comparison (next format file) was performed using ClustalW software.^55^ A cluster topology including 24 ethnic minorities was created using MrBayes 3.2.0,^56^ and the parameters were descripted in the supplementary text.

#### Distribution patterns of EMMPs

We divided China’s map into 4,069 50 km × 50 km grids using ArcGIS 10.6 for distribution analysis. Five algorithms, namely species richness (SR), SC, weighted endemism (WE), phylogenetic diversity (PD), and phylogenetic endemism (PE) were employed to assess the distribution patterns of all species, endemic species, and threatened species of EMMPs. SR, the most conventional and commonly used quantitative indicator of species diversity hotspots,^62^^,^^63^^,^^64^ was caclculated following Xue et al. (2023).^28^ In accordance with previous studies,^8^^,^^18^^,^^65^ SC, aiming to determine the minimum area that can contain all species^18^^,^^66^ was employed. WE, acconting for species range size, was calculated weighted range size rarity.^28^^,^^67^^,^^68^

Species with distinct evolutionary history deserve greater attention, given evolution’s unpredictability and irreversibility.^69^ PD, integrating SR and phylogenetic data, was comprehensively assessed for all the studied taxa. Maximizing the genetic diversity of protected species, it is widely applied to identify species distribution patterns and conservation priority areas.^70^ However, PD disregards the spatial distribution range of species, which is key for the setting of conservation priorities. Hence, PE was developed to integrate the distribution range of a species and its phylogenetic history.^70^ To accurately calculate the PD and PE indices, we conducted the phylogenetic framework using the package “*V.PhyloMaker2*” in R 4.2.2,^71^ and a phylogenetic tree was constructed that included 5,805 species (99.74%, with 15 bryophyte species omitted). PE and PD were then computed using Biodiverse 3.1 to further analyze the distribution patterns of EMMPs.

Following Xue et al. (2021),^47^ we measure the phylogenetic analysis (PA) of each grid cell in hotspot identification. Then we converted the values of PD and PE in each grid cell into ratios of their maximum values (designated PD_max and PE_max, respectively).

PA = PD/PD__max_ + PE/PE__max_

#### Diversity hotspots of EMMPs and correlations among distribution patterns

In previous studies, the threshold of top 5% and top 10% of the total land area of study region were commonly used as thresholds to define hotspots.^8^^,^^41^^,^^70^ In addition, according to the Aichi biodiversity targets and “30×30” biodiversity targets, 17% and 30% of the land area should be protected, respectively (https://www.cbd.int/gbf/targets). Therefore, in this study, we defined the top 5%, top 10%, top 17%, and top 30% of the total land area of China with the highest SR, SC, WE, and PA as hotspots.^20^^,^^72^^,^^73^ Then, we overlaid these hotspots at different thresholds to obtain the final hotspots of EMMPs, and subsequently the hotspot grids identified by different methods at the same threshold were overlaid separately. Since the number of grids after overlay would exceed the number corresponding to the hotspot threshold, we ensured the final number of comprehensive hotspot grids matched that of the threshold. This study summed and sorted the ranking values of hotspot grids from each method after overlay,^9^ selecting the top 5%, 10%, 17%, and 30% of grids as diversity hotspots at the four threshold levels. Grids identified by all four algorithms were classified as Class-I hotspots, those identified by three algorithms as Class-II hotspots, those identified by two algorithms as Class-III hotspots.

The four final hotspot types, corresponding to the hotspot thresholds of top 5%, top 10%, top 17%, and top 30%, were used to delineate hotspot areas and gaps, and analyze conservation effectiveness of existing conservation networks. In addition, we calculated Pearson correlation coefficient (r) values between distribution patterns to detect correlations of distribution patterns among EMMPs using the “*corrplot*” package in R.^74^ We named hotspot areas and distribution patterns base on administrative subdivision and mountains (Figure S2).

#### Identification of conservation effectiveness and gaps for EMMPs

The network of nature reserves in China plays a significant role inconserving biodiversity, natural landscapes, and ecosystem services.^75^^,^^76^ To date, > 2,700 nature reserves have been established in China, covering almost 15% of China’s total land area (Ministry of Ecology and Environment of the People’s Republic of China. 2018). These national and provincial nature reserves (NNRs and PNRs, respectively) have clear boundaries, and strict,standardized management, making them cornerstones of China’ biodiversity conservation.^8^^,^^66^^,^^77^^,^^78^ Here, we selected NNRs and PNRs to assess the current conservation network’s effectiveness network for EMMPs. First, we compiled geo-document layers of nature reserves based on the official list (http://www.mee.gov.cn) and documents from the World Database on Protected Areas (https://www.protectedplanet.net/), obtaining 464 NNRs and 806 PNRs. Then, we overlaid the final hotspot maps withof NNR and PNR distributions. A grid cell with no nature reserves was identified as a conservation gap.^8^^,^^28^^,^^66^ while one covered by a nature reserve was deemed effectively protected.

#### Effects of climatic and environmental conditions on the potential distribution areas of endemic and endangered species of EMMPs

Rare and endangered species, sensitivity to environmental changes, can serve as indicators of climate change on biodiversity.^79^ Here, 2,138 endemic and endangered EMMPs were selected for prediction analysis (Table S2). First, we obtained 19 bioclimatic variables (Bio1–Bio19) at 10 min resolution for the current (1960–1990, WorldClim, version 1.4) and future (2070, CMIP5) climate data, as well as elevation data (http://www.worldclim.org). Future climate scenarios were derived from the circulation models of BCC-CSM1-1 under four different RCPs (RCP 2.6, RCP 4.5, RCP 6.0, and RCP 8.5). To avoid multicollinearity and overfitting, we screened environmental variables (r > 0.85) using the Pearson correlation analysis function in the package “*complot*” in R. Nine variables (altitude, Bio1, Bio2, Bio 3, Bio4, Bio12, Bio14, Bio15, and Bio18) were retained for further analysis (Table S3). For predicting potential distributions, five widely used, high-performance models (generalized linear model, general additive model, generalized boosting model, random forest, and maximum entropy model), were employed using the package *sdm* in R.^65^^,^^80^^,^^81^^,^^82^ Then, we generated 358,913 distribution data by randomly selecting a distribution point for each species in each grid cell with a precision of 10 min to minimize sampling bias, particularly the impact of spatial autocorrelation on model accuracy.^82^^,^^83^^,^^84^

Subsequently, we randomly produced two sets of pseudo-absence records with a moderate quantity of data (10,000), as all models require data from species absence records.^85^^,^^86^^,^^87^ We integrated the species occurrence and species pseudo-absence points, randomly spliting 70% of occurrence points for training and 30% for evaluation. To ensure robust, model ran 10 times, with performance assessed using true skill statistic (TSS) and the area under the receiver operating characteristic curve (AUC) to assess model performance. Only models with TSS ≥ 0.5 and AUC ≥ 0.7 were retained to build a TSS-weighted ensamble model.^88^^,^^89^

Utilizing a threshold that maximized TSS, we transformed the species habitat suitability map into a binary (presence/absence) distribution map to create a species diversity map.^81^^,^^86^^,^^90^^,^^91^ Finally, to collect SR data for the current and four future climate scenarios, we created additive distribution maps of all predicted species per grid cell. We calculated the SR differences between current and future climate scenarios. Then, we visualized the current and future SR distribution and their discrepancies to explore climate change’s impact on EMMPs.

### Quantification and statistical analysis

Quantification and statistical analysis are presented in method details.

## Supplemental information

Document S1. Figures S1–S36 and Tables S3–S7

Table S1. Species list of Chinese ethnic minority medicinal plants included in this study

Table S2. True skill statistic (TSS) and area under the operating characteristic curve (AUC) values of RCMPs selected for species distribution model and their climatically suitable habitat areas under future (2070) climate scenarios (RCP 2.6, RCP 4.5, RCP 6.0, and RCP 8.5)

## References

[1] Wake D.B., Vredenburg V.T. (2008). Colloquium paper: Are we in the midst of the sixth mass extinction? A view from the world of amphibians. Proc. Natl. Acad. Sci. USA.

[2] Barnosky A.D., Matzke N., Tomiya S., Wogan G.O.U., Swartz B., Quental T.B., Marshall C., McGuire J.L., Lindsey E.L., Maguire K.C. (2011). Has the Earth’s sixth mass extinction already arrived?. Nature.

[3] Kemp M.E., Boville A.E., Carneiro C.M., Jacisin J.J., Law C.J., Ledesma D.T., Meza A., Shields-Estrada A., Xu T. (2023). Looking back for the future, The ecology of terrestrial communities through the lens of conservation paleobiology. Annu. Rev. Ecol. Evol. Syst..

[4] Humphreys A.M., Govaerts R., Ficinski S.Z., Nic Lughadha E., Vorontsova M.S. (2019). Global dataset shows geography and life form predict modern plant extinction and rediscovery. Nat. Ecol. Evol..

[5] Albani Rocchetti G., Carta A., Mondoni A., Godefroid S., Davis C.C., Caneva G., Albrecht M.A., Alvarado K., Bijmoer R., Borosova R. (2022). Selecting the best candidates for resurrecting extinct-in-the-wild plants from herbaria. Nat. Plants.

[6] Kubiak L. (2020).

[7] Huang G., Ping X., Xu W., Hu Y., Chang J., Swaisgood R.R., Zhou J., Zhan X., Zhang Z., Nie Y. (2021). Wildlife conservation and management in China: achievements, challenges and perspectives. Natl. Sci. Rev..

[8] Chi X., Zhang Z., Xu X., Zhang X., Zhao Z., Liu Y., Wang Q., Wang H., Li Y., Yang G. (2017). Threatened medicinal plants in China, Distributions and conservation priorities. Biol. Conserv..

[9] Xia C., Huang Y., Qi Y., Yang X., Xue T., Hu R., Deng H., Bussmann R.W., Yu S. (2022). Developing long-term conservation priority planning for medicinal plants in China by combining conservation status with diversity hotspot analyses and climate change prediction. BMC Biol..

[10] Huang J., Zheng Y., Wu W., Xie T., Yao H., Pang X., Sun F., Ouyang L., Wang J. (2015). CEMTDD, The database for elucidating the relationships among herbs, compounds, targets and related diseases for Chinese ethnic minority traditional drugs. Oncotarget.

[11] Wang S. (2020). Annual advances of Chinese minority traditional medicine in 2019. Tradit. Med. Res..

[12] Jia M., Zhang Y. (2016).

[13] Zaman W., Ye J.F., Ahmad M., Saqib S., Shinwari Z.K., Chen Z.D. (2022). Phylogenetic exploration of traditional Chinese medicinal plants, a case study on Lamiaceae. Pak. J. Bot..

[14] Huang J., Chen B., Liu C., Lai J., Zhang J., Ma K. (2012). Identifying hotspots of endemic woody seed plant diversity in China. Divers. Distrib..

[15] Wei S., Sun T., Du J., Zhang B., Xiang D., Li W. (2018). Xanthohumol, a prenylated flavonoid from Hops, exerts anticancer effects against gastric cancer in vitro. Oncol. Rep..

[16] Li J.C., He X.L., Xu X., Wang Y., Huang L.P. (2019). Overview of modern research and development of Mongolian medicine. J. Southwest. Minzu. Univ. (Nat Sci Edit).

[17] Sun X.M., Zhang X.H., Wang H.F., Zhu G.W. (2020). Industry status and development strategies of Chinese ethnic medicine. Chinese J. Exp. Tradit. Med. Formulae..

[18] Dobson A.P., Rodriguez J.P., Roberts W.M., Wilcove D.S. (1997). Geographic distribution of endangered species in the United States. Science.

[19] Linder H.P. (2001). Plant diversity and endemism in sub-Saharan tropical Africa. J. Biogeogr..

[20] Huang J., Huang J., Liu C., Zhang J., Lu X., Ma K. (2016). Diversity hotspots and conservation gaps for the Chinese endemic seed fora. Biol. Conserv..

[21] Baldwin B.G., Thornhill A.H., Freyman W.A., Ackerly D.D., Kling M.M., Morueta-Holme N., Mishler B.D. (2017). Species richness and endemism in the native flora of California. Am. J. Bot..

[22] Yu F., Skidmore A.K., Wang T., Huang J., Ma K., Groen T.A. (2017). Rhododendron diversity patterns and priority conservation areas in China. Divers. Distrib..

[23] Rosauer D., Laffan S.W., Crisp M.D., Donnellan S.C., Cook L.G. (2009). Phylogenetic endemism, a new approach for identifying geographical concentrations of evolutionary history. Mol. Ecol..

[24] Voskamp A., Fritz S.A., Köcke V., Biber M.F., Nogueira Brockmeyer T., Bertzky B., Forrest M., Goldstein A., Henderson S., Hickler T. (2023). Utilizing multi-objective decision support tools for protected area selection. One Earth.

[25] Tang C.Q., Matsui T., Ohashi H., Dong Y.F., Momohara A., Herrando-Moraira S., Qian S., Yang Y., Ohsawa M., Luu H.T. (2018). Identifying long-term stable refugia for relict plant species in East Asia. Nat. Commun..

[26] Penteriani V., Zarzo-Arias A., Novo-Fernández A., Bombieri G., López-Sánchez C.A. (2019). Responses of an endangered brown bear population to climate change based on predictable food resource and shelter alterations. Glob. Chang. Biol..

[27] Sales L., Ribeiro B.R., Chapman C.A., Loyola R. (2020). Multiple dimensions of climate change on the distribution of Amazon primates. Perspect. Ecol. Conserv..

[28] Xue T.T., Yang X.D., Liu Q., Qin F., Zhang W.D., Janssens S.B., Yu S.X. (2023). Integration of hotspot identification, gap analysis, and niche modeling supports the conservation of Chinese threatened higher plants. J. Syst. Evol..

[29] Qin F., Zhang X.X., Huang Y.F., Wu L., Xu W.B., Xue T.T., Zhang W., Liu Q., Yu J., Gao J. (2023). Geographic distribution, conservation effectiveness, and gaps for national key protected wild plants in China. J. Syst. Evol..

[30] Hu X.G., Jin Y., Wang X.R., Mao J.F., Li Y. (2015). Predicting impacts of future climate change on the distribution of the widespread conifer Platycladus orientalis. PLoS One.

[31] Yang X., Zhang W., Qin F., Yu J., Xue T., Huang Y., Xu W., Wu J., Smets E.F., Yu S. (2022). Biodiversity priority areas and conservation strategies for seed plants in China. Front. Plant Sci..

[32] Huang L.Q., Xiao P.G., Wang Y.Y. (2012).

[33] Qin F., Xue T., Yang X., Zhang W., Wu J., Huang Y., Khan G., Yu S. (2022). Conservation status of threatened land plants in China and priority sites for better conservation targets, distribution patterns and conservation gap analysis. Biodivers. Conserv..

[34] Ye C., Liu H., Qin H., Shu J., Zhou Z., Jin X. (2023). Geographical distribution and conservation strategy of national key protected wild plants of China. iScience.

[35] Volis S., Blecher M. (2010). Quasi in situ, a bridge between ex situ and in situ conservation of plants. Biodivers. Conserv..

[36] Sun W., Han C. (2015). Researches and conservation for plant species with extremely small populations (PSESP). Biodivers. Sci..

[37] Zang R.G. (2020). Research progress in Wild Plant with Extremely Small Populations in China. Biodivers. Sci..

[38] Chen S.L., Yu H., Luo H.M., Wu Q., Li C.F., Steinmetz A. (2016). Conservation and sustainable use of medicinal plants, problems, progress, and prospects. Chin. Med..

[39] Zhao X., Chen H., Wu J., Ren H., Wei J., Ye P., Si Q. (2022). Ex situ conservation of threatened higher plants in Chinese botanical gardens. Glob. Ecol. Conserv..

[40] Ye H., Liu S.L., Zhai Y.S., Huang M.J., Zhang L.D. (2014). Study on sustainable development of industry of ethnic medicine in minority area. China J. Chinese Mater. Med..

[41] Shrestha N., Wang Z. (2018). Selecting priority areas for systematic conservation Of Chinese Rhododendron, hotspot versus complementarity approaches. Biodivers. Conserv..

[42] Milliken W., Walker B.E., Howes M.J.R., Forest F., Nic Lughadha E. (2021). Plants used traditionally as antimalarials in Latin America, Mining the tree of life for potential new medicines. J. Ethnopharmacol..

[43] Zaman W., Ye J., Saqib S., Liu Y., Shan Z., Hao D., Chen Z., Xiao P. (2021). Predicting potential medicinal plants with phylogenetic topology: Inspiration from the research of traditional Chinese medicine. J. Ethnopharmacol..

[44] Cheng Z., Zhang Q., Long C. (2022). Research status of ethnobotany (2017–2022). Biodivers. Sci..

[45] Zhong G.Y., Wang C.H., Zhao J.F., Gu R., Qin S.Y. (2009). Methods for studying national medicinal resources and sustainable utilization of TCM resources. World. Sci. Technol..

[46] Su G.Q., Li H.T., Sun H., Zhang X.B., Zhang L.X., Li Y.J., Huang L.Q., Ma X.J. (2017). Endemic plants for medicine use in China. China J. Chinese Mater. Med..

[47] Xue T., Gadagkar S.R., Albright T.P., Yang X., Li J., Xia C., Wu J., Yu S. (2021). Prioritizing conservation of biodiversity in an alpine region, Distribution pattern and conservation status of seed plants in the Qinghai-Tibetan Plateau. Glob. Ecol. Conserv..

[48] Zomer R.J., Xu J., Wang M., Trabucco A., Li Z. (2015). Projected impact of climate change on the effectiveness of the existing protected area network for biodiversity conservation within Yunnan Province, China. Biol. Conserv..

[49] Attorre F., Abeli T., Bacchetta G., Farcomeni A., Fenu G., De Sanctis M., Gargano D., Peruzzi L., Montagnani C., Rossi G. (2018). How to include the impact of climate change in the extinction risk assessment of policy plant species?. J. Nat. Conserv..

[50] Faith D.P. (2013). Biodiversity and evolutionary history, useful extensions of the PD phylogenetic diversity assessment framework. Ann. N. Y. Acad. Sci..

[51] Qin H., Zhao L., Yu S., Liu H., Liu B., Xia N., Peng H., Li Z., Zhang Z., He X. (2017). Evaluating the endangerment status of China’s angiosperms through the red list assessment. Biodivers. Sci..

[52] Su J.Y., Yan Y., Li C., Li D., Du F. (2020). Informing conservation strategies with genetic diversity in Wild Plant with Extremely Small Populations, a review on gymnosperms. Biodivers. Sci..

[53] Zhang Y., Qian L., Spalink D., Sun L., Chen J., Sun H. (2021). Spatial phylogenetics of two topographic extremes of the Hengduan Mountains in southwestern China and its implications for biodiversity conservation. Plant Divers..

[54] Luo A., Li Y., Shrestha N., Xu X., Su X., Li Y., Lyu T., Waris K., Tang Z., Liu X. (2024). Global multifaceted biodiversity patterns, centers, and conservation needs in angiosperms. Sci. China Life Sci..

[55] Thompson J.D., Higgins D.G., Gibson T.J. (1994). CLUSTAL W: improving the sensitivity of progressive multiple sequence alignment through sequence weighting, position-specific gap penalties and weight matrix choice. Nucleic Acids Res..

[56] Ronquist F., Teslenko M., Van Der Mark P., Ayres D.L., Darling A., Höhna S., Larget B., Liu L., Suchard M.A., Huelsenbeck J.P. (2012). MrBayes 3.2: efficient Bayesian phylogenetic inference and model choice across a large model space. Syst. Biol..

[57] Wu Z.Y., Raven P.H. (1994).

[58] Anderson S. (1994). Area and endemism. Q. Rev. Biol..

[59] Qin H.N. (2020).

[60] Giday M., Asfaw Z., Woldu Z., Teklehaymanot T. (2009). Medicinal plant knowledge of the Bench ethnic group of Ethiopia, an ethnobotanical investigation. J. Ethnobiol. Ethnomed..

[61] Teklay A., Abera B., Giday M. (2013). An ethnobotanical study of medicinal plants used in Kilte Awulaelo District, Tigray region of Ethiopia. J. Ethnobiol. Ethnomed..

[62] Orme C.D.L., Davies R.G., Burgess M., Eigenbrod F., Pickup N., Olson V.A., Webster A.J., Ding T.S., Rasmussen P.C., Ridgely R.S. (2005). Global hotspots of species richness are not congruent with endemism or threat. Nature.

[63] Sun S., Huang B., Wu R.D., Zhou R.L. (2013). The spatial distribution patterns of rare plants and endemic species in China. Yunnan. Geogr. Environ. Res..

[64] Li R. (2019). Protecting rare and endangered species under climate change on the Qinghai Plateau, China. Ecol. Evol..

[65] Qin F., Han B.C., Bussmann R.W., Xue T.T., Liang Y.F., Zhang W.D., Liu Q., Chen T., Yu S. (2024). Present status, future trends, and control strategies of invasive alien plants in China affected by human activities and climate change. Ecography.

[66] Zhang Z., He J.S., Li J., Tang Z. (2015). Distribution and conservation of threatened plants in China. Biol. Conserv..

[67] Williams P.H., Prance G.T., Humphries C.J., Edwards K.S. (1996). Promise and problems in applying quantitative complementary areas for representing the diversity of some neotropical plants (families Dichapetalaceae, Lecythidaceae, Caryocaraceae, Chrysobalanaceae and Proteaceae). Biol. J. Linn. Soc..

[68] Xu W.B., Svenning J.C., Chen G.K., Zhang M.G., Huang J.H., Chen B., Ordonez A., Ma K.P. (2019). Human activities have opposing effects on distributions of narrow-ranged and widespread plant species in China. Proc. Natl. Acad. Sci. USA.

[69] Ci X., Li J. (2017). Phylogenetic diversity and its application in floristics and biodiversity conservation. Biodivers. Sci..

[70] Xu Y., Shen Z., Ying L., Wang Z., Huang J., Zang R., Jiang Y. (2017). Hotspot analyses indicate significant conservation gaps for evergreen broadleaved woody plants in China. Sci. Rep..

[71] Jin Y., Qian H. (2022). V.PhyloMaker2, An updated and enlarged R package that can generate very large phylogenies for vascular plants. Plant Divers..

[72] Grenyer R., Orme C.D.L., Jackson S.F., Thomas G.H., Davies R.G., Davies T.J., Jones K.E., Olson V.A., Ridgely R.S., Rasmussen P.C. (2006). Global distribution and conservation of rare and threatened vertebrates. Nature.

[73] Li G., Xiao N., Luo Z., Liu D., Zhao Z., Guan X., Zang C., Li J., Shen Z. (2021). Identifying conservation priority areas for gymnosperm species under climate changes in China. Biol. Conserv..

[74] Wei T.Y., Simko V.R. (2017). package “corrplot”: Visualization of a Correlation Matrix (Version 0.84). https://github.com/taiyun/corrplot.

[75] Tang X.P., Jiang Y.F., Liu Z.L., Chen J.Z., Liang B.K., Lin C. (2019). Top-level design of the natural protected area system in China. Forest. Resour. Manag..

[76] Gao J., Liu X., Zhou D., Ma K., Wu Q., Li G. (2021). Some opinions on the integration and optimization of natural protected areas in China. Biodivers. Sci..

[77] Quan J., Ouyang Z.Y., Xu W.H., Miao H. (2009). Management effectiveness of China nature reserves status quo assessment and counter measures. Chin. J. Appl. Ecol..

[78] Zhao G.H., Tian Y., Tang Z.Y., Li J.S., Zeng H. (2013). Distribution of terrestrial national nature reserves in relation to human activities and natural environments in China. Biodivers. Sci..

[79] Chen I.C., Hill J.K., Ohlemüller R., Roy D.B., Thomas C.D. (2011). Rapid range shifts of species associated with high levels of climate warming. Science.

[80] Naimi B., Araújo M.B. (2016). Sdm, a reproducible and extensible R platform for species distribution modelling. Ecography.

[81] Dullinger I., Wessely J., Bossdorf O., Dawson W., Essl F., Gattringer A., Klonner G., Kreft H., Kuttner M., Moser D. (2017). Climate change will increase the naturalization risk from garden plants in Europe. Glob. Ecol. Biogeogr..

[82] Liu C., Comte L., Xian W., Chen Y., Olden J.D. (2019). Current and projected future risks of freshwater fish invasions in China. Ecography.

[83] Zhang J., Jiang F., Li G., Qin W., Li S., Gao H., Cai Z., Lin G., Zhang T. (2019). Maxent modeling for predicting the spatial distribution of three raptors in the Sanjiangyuan National Park, China. Ecol. Evol..

[84] Zhu G., Gutierrez Illan J., Looney C., Crowder D.W. (2020). Assessing the ecological niche and invasion potential of the Asian giant hornet. Proc. Natl. Acad. Sci. USA.

[85] Wisz M.S., Guisan A. (2009). Do pseudo-absence selection strategies influence species distribution models and their predictions? An information-theoretic approach based on simulated data. BMC Ecol..

[86] Barbet-Massin M., Jiguet F., Albert C.H., Thuiller W. (2012). Selecting pseudo-absences for species distribution models, how, where and how many?. Methods Ecol. Evol..

[87] Yan Y., Li Y., Wang W.J., He J.S., Yang R.H., Wu H.J., Wang X.L., Jiao L., Tang Z., Yao Y.J. (2017). Range shifts in response to climate change of Ophiocordyceps sinensis, a fungus endemic to the Tibetan Plateau. Biol. Conserv..

[88] Thuiller W., Lafourcade B., Engler R., Araújo M.B. (2009). BIOMOD-a platform for ensemble forecasting of species distributions. Ecography.

[89] Gallardo B., Aldridge D.C., González-Moreno P., Pergl J., Pizarro M., Pyšek P., Thuiller W., Yesson C., Vilà M. (2017). Protected areas offer refuge from invasive species spreading under climate change. Glob. Chang. Biol..

[90] Thuiller W., Pironon S., Psomas A., Barbet-Massin M., Jiguet F., Lavergne S., Pearman P.B., Renaud J., Zupan L., Zimmermann N.E. (2014). The European functional tree of bird life in the face of global change. Nat. Commun..

[91] Fourcade Y., Besnard A.G., Secondi J. (2018). Paintings predict the distribution of species, or the challenge of selecting environmental predictors and evaluation statistics. Glob. Ecol. Biogeogr..

