# Supplementary material for: Conservation status of ethnic minority medicinal plants in China for the thirty by thirty target
Source: iScience. 2026 Jun 1;29(6):116140. doi: 10.1016/j.isci.2026.116140 (PMC13233616; doi:10.1016/j.isci.2026.116140)
Supplement: Document S1. Figures S1–S36 and Tables S3–S7 [file mmc1.pdf]

iScience, Volume *29*

Supplemental information

Conservation status of ethnic minority medicinal plants in China for the thirty by thirty target

Baocai Han, Jiejing Gao, Changying Xia, Ruyu Yao, Xiaoxia Zhang, Yunfeng Huang, Yude Peng, Tiantian Xue, Chong Luo, Rainer W. Bussmann, Yaodong Qi, and Shengxiang Yu

Supplemental Figures and Tables

Figure S1. Distribution map of 24 selected ethnic minorities. Different graphics represent different ethnic minorities.

Figure S2. Map of the main mountain ranges and administrative divisions in China.

Figure S3. Cluster and composition analysis of EMMPs.

Figure S4. Spatial distribution patterns of species richness (SR) for different plant categories.

Figure S5. Correlogram of distribution patterns of EMMPs in different categories.

Figure S6. Spatial distribution patterns of species richness (SR) for different endemic plant categories.

Figure S7. Spatial distribution patterns of species richness (SR) for different threatened plant categories.

Figure S8. Spatial distribution patterns of species complementarity (SC) for different plant categories.

Figure S9. Spatial distribution patterns of species complementarity (SC) for different endemic plant categories.

Figure S10. Spatial distribution patterns of species complementarity (SC) for different threatened plant categories.

Figure S11. Spatial distribution patterns of weighted endemism (WE) for different plant categories.

Figure S12. Spatial distribution patterns of weighted endemism (WE) for different endemic plant categories.

Figure S13. Spatial distribution patterns of weighted endemism (WE) for different threatened plant categories.

Figure S14. Spatial distribution patterns of phylogenetic diversity (PD) for different plant categories.

Figure S15. Spatial distribution patterns of phylogenetic endemism (PE) for different plant categories.

Figure S16. Spatial distribution patterns of top 5% hotspots of different algorithms.

Figure S17. Spatial distribution patterns of diversity hotspots for all EMMPs of TJ-YA clade at thresholds of top 5% (A), top 10% (B), top17 % (C), and top 30% (D).

Figure S18. Spatial distribution patterns of diversity hotspots for all EMMPs of DA-HN clade at thresholds of top 5% (A), top 10% (B), top17 % (C), and top 30% (D).

Figure S19. Spatial distribution patterns of diversity hotspots for all EMMPs of YI-LS clade at thresholds of top 5% (A), top 10% (B), top17 % (C), and top 30% (D).

Figure S20. Spatial distribution patterns of diversity hotspots for all EMMPs of DE-JP clade at thresholds of top 5% (A), top 10% (B), top17 % (C), and top 30% (D).

Figure S21. Spatial distribution patterns of diversity hotspots for all EMMPs of ZA-MG clade at thresholds of top 5% (A), top 10% (B), top17 % (C), and top 30% (D).

Figure S22. Statistics of conservation effectiveness of all EMMPs (A), endemic EMMPs (B), threatened EMMPs (C), and protected EMMPs (D) at thresholds of top 5%, top 10%, top17 %, and top 30%.

Figure S23. Spatial distribution patterns of conservation effectiveness and gaps for all EMMPs of TJ-YA clade based on four different thresholds.

Figure S24. Spatial distribution patterns of conservation effectiveness and gaps for all EMMPs

of DA-HN clade based on different four thresholds.

Figure S25. Spatial distribution patterns of conservation effectiveness and gaps for all EMMPs of YI-LS clade based on different four thresholds.

Figure S26. Spatial distribution patterns of conservation effectiveness and gaps for all EMMPs of DE-JP clade based on different four thresholds.

Figure S27. Spatial distribution patterns of conservation effectiveness and gaps for all EMMPs of ZA-MG clade based on different four thresholds.

Figure S28. Changes in range sizes of endemic and threatened EMMPs under four emission scenarios: representative concentration pathway [RCP] 2.6 (A), RCP 4.5 (B), RCP 6.0 (C), and RCP 8.5 (D).

Figure S29. Spatial distribution patterns of species richness for different plant categories based on the results of MaxEnt. Time periods: near current (1960–1990).

Figure S30. Spatial distribution patterns of species richness for different plant categories based on the results of MaxEnt. Time periods: future (2070). Emission scenarios: Representative concentration pathway (RCP) 2.6.

Figure S31. Spatial distribution patterns of species richness for different plant categories based on the results of MaxEnt. Time periods: future (2070). Emission scenarios: Representative concentration pathway (RCP) 4.5.

Figure S32. Spatial distribution patterns of species richness for different plant categories based on the results of MaxEnt. Time periods: future (2070). Emission scenarios: Representative concentration pathway (RCP) 6.0.

Figure S33. Spatial distribution patterns of species richness for different plant categories based on the results of MaxEnt. Time periods: future (2070). Emission scenarios: Representative concentration pathway (RCP) 8.5.

Figure S34. Species richness change for different plant categories by 2070 under the emission scenarios of representative concentration pathway [RCP] 4.5.

Figure S35. Species richness change for different plant categories by 2070 under the emission scenarios of representative concentration pathway [RCP] 6.0.

Figure S36. Species richness change for different plant categories by 2070 under the emission scenarios of representative concentration pathway [RCP] 8.5.

Table S3. Environmental variables used in the species distribution modeling process.

Table S4. Numbers and proportions of grids in hotspots and nature reserves.

Table S5. Numbers and proportions of species in hotspots and nature reserves.

Table S6. Number of species with potential change in distribution area.

Table S7. Number of grids with potential change in distribution area.

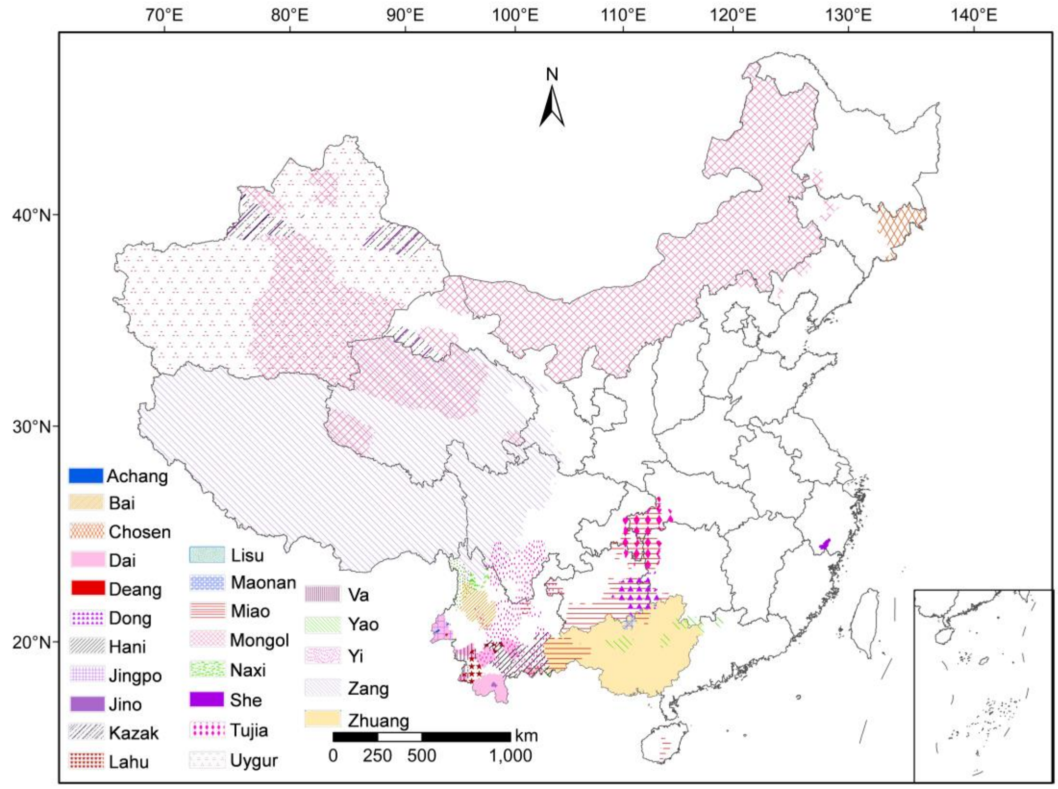

Figure S1. Distribution map of 24 selected ethnic minorities. Different graphics represent different ethnic minorities.

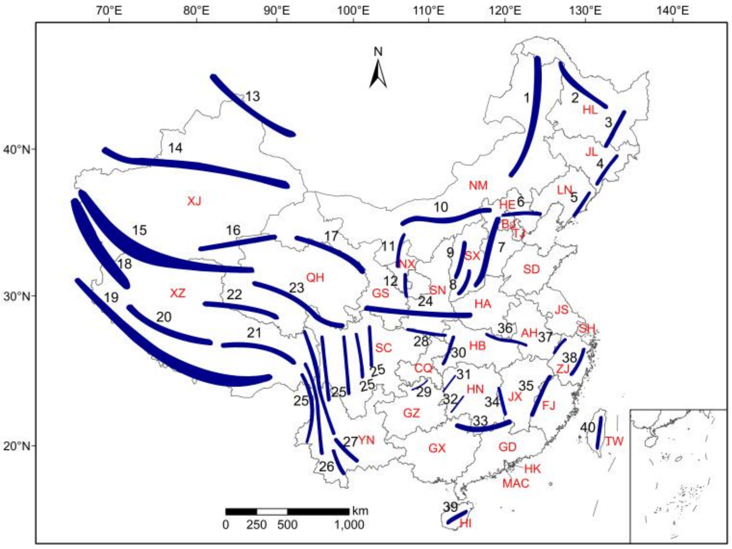

Figure S2. Map of the main mountain ranges and administrative divisions in China: 1. Daxing'anling Mountains, 2. Xiaoxing'anling Mountains, 3. Changbai Mountains, 4. Zhangguangcailing Mountains, 5. Longgang Mountains, 6. Yanshan Mountains, 7. Taihang Mountains, 8. Zhongtiao Mountains, 9. Luliang Mountains, 10. Yinshan Mountains, 11. Helan Mountains, 12. Liupan Mountains, 13. Altai Mountains 14. Tianshan Mountains, 15. Kunlun Mountains, 16. Aerjin Mountains, 17. Qilian Mountains, 18. Karakorum Mountains, 19. Himalayas, 20. Gangdisi Mountains, 21. Nyainqntanglha Mountains, 22. Danggula Mountains, 23. Bayankala Mountains, 24. Qinling Mountains, 25. Hengduan Mountains, 26. Wuliang Mountains, 27. Ailao Mountains, 28. Bashan Mountains, 29. Dalou Mountains, 30. Wushan Mountains, 31. Wuling Mountains, 32. Xuefeng Mountains, 33. Nanling Mountains, 34. Luoxiao Mountains, 35. Wuyi Mountains, 36. Dabie Mountains, 37. Tianmu Mountains, 38. Yandang Mountains, 39. Wuzhi Mountains, 40. Yushan Mountains (Wang et al., 2004). The red characters represent China's administrative divisions: Heilongjiang (HL), Jilin (JL), Liaoning (LN), Neimeng (NM), Hebei (HE), Beijing (BJ), Tianjing (TJ), Xinjiang (XJ), Xizang (XZ), Qinghai (QH), Gansu (GS), Ningxia (NX), Shaanxi (SN), Shanxi (SX), Shandong (SD), Henan (HA), Sichuan (SC), Chongqing (CQ), Hubei (HB), Anhui (AH), Jiangsu (JS), Shanghai (SH), Yunnan (YN), Guizhou (GZ), Hunan (HN), Jiangxi (JX), Zhejiang (ZJ), Fujian (FJ), Guangxi (GX), Guangdong (GD), Taiwan (TW), Hong Kong (HK), Macau (MAC), and Hainan (HI).

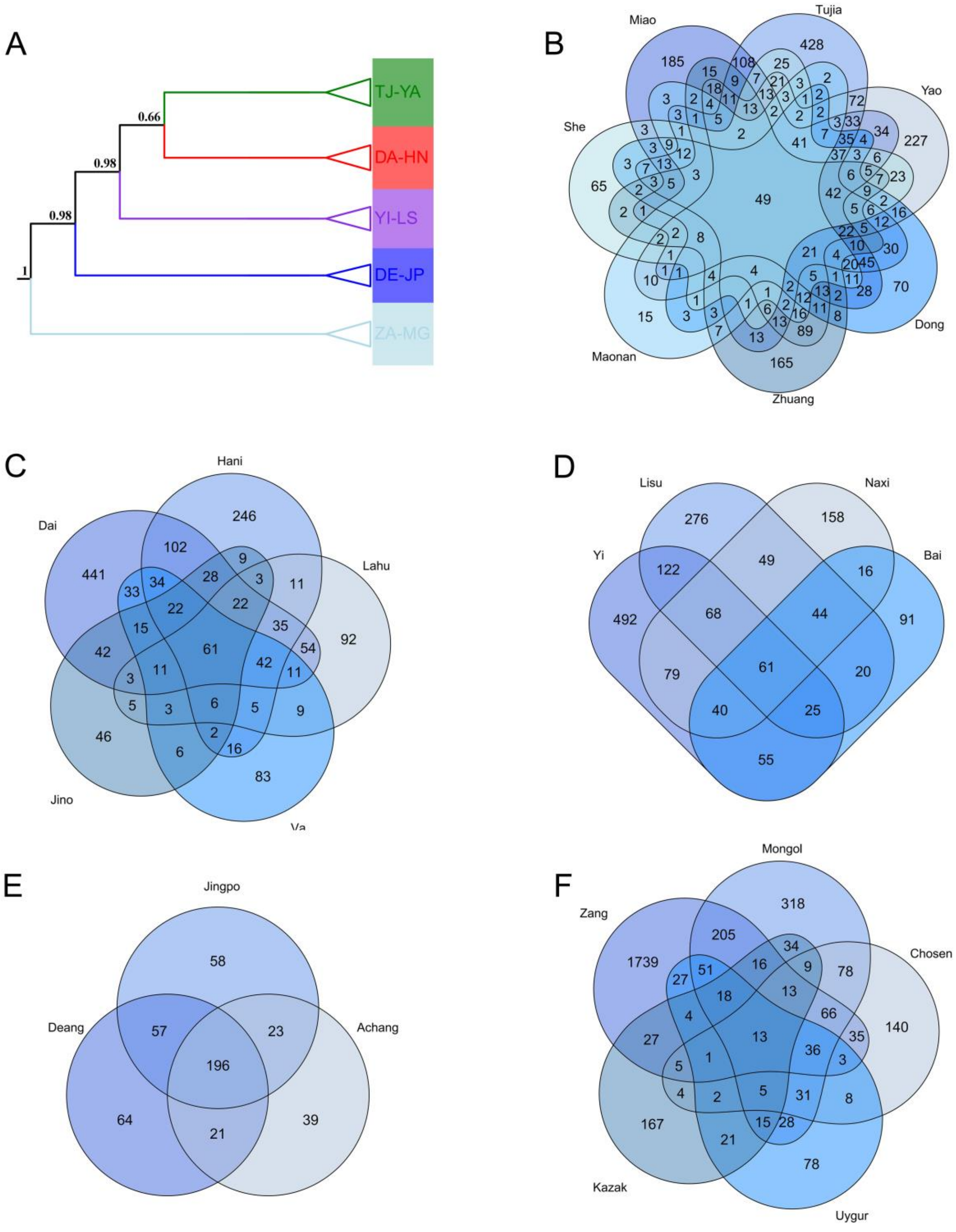

Figure S3. Cluster and composition analysis of EMMPs. (A) Cluster analysis of all EMMPs of 24 ethnic minorities. (B-F) Species composition of EMMPs of TJ-YA clade (B), DA-HN clade (C), YI-LS clade (D), DE-JP clade (E), and ZA-MG clade (F).

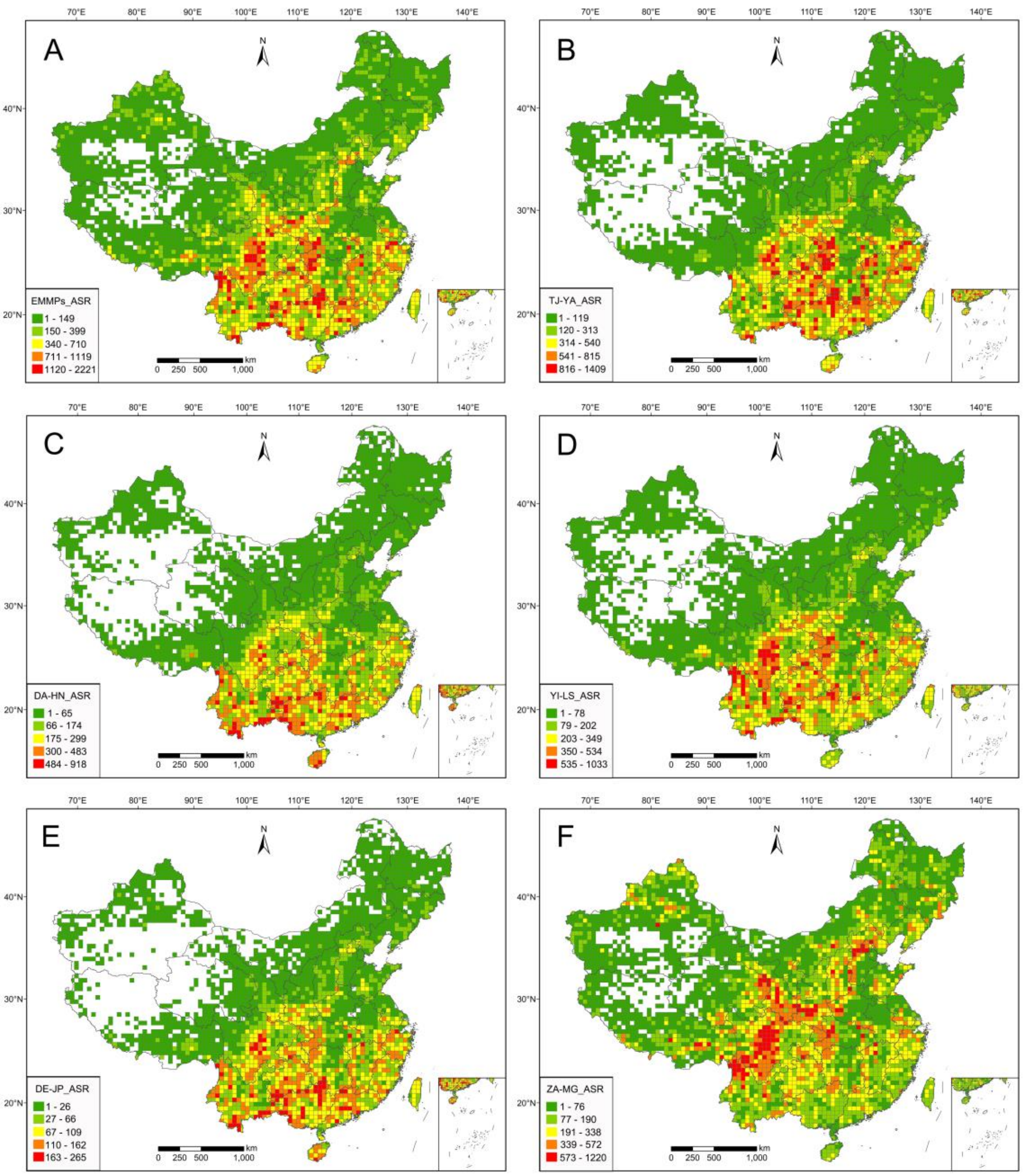

Figure S4. Spatial distribution patterns of species richness (SR) for different plant categories: (A) all EMMPs of 24 ethnic minorities, (B) all EMMPs of TJ-YA clade, (C) all EMMPs of DA-HN clade, (D) all EMMPs of YI-LS clade, (E) all EMMPs of DE-JP clade, (F) all EMMPs of ZA-MG clade.

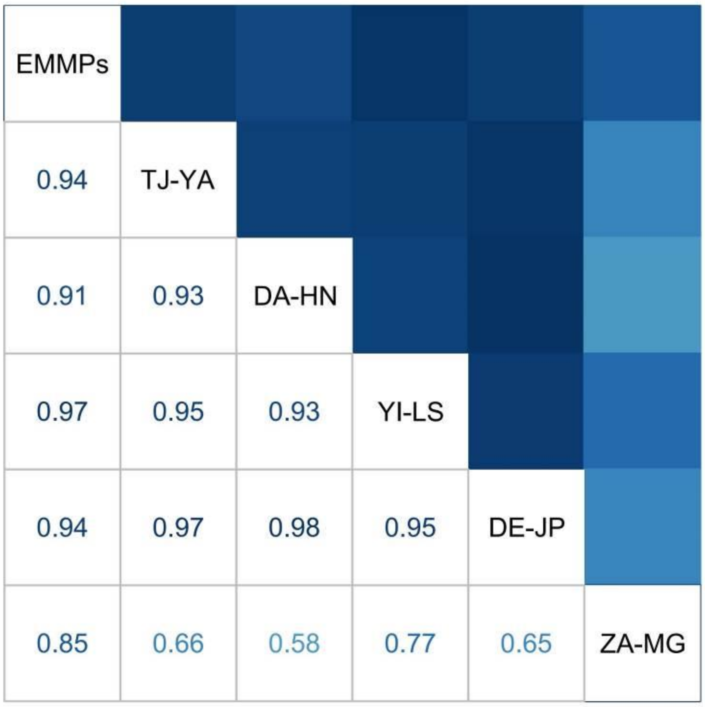

Figure S5. Correlogram of distribution patterns of EMMPs in different categories. Distribution patterns of all EMMPs of 24 ethnic minorities (EMMPs), all EMMPs of TJ-YA clade (TJ-YA), all EMMPs of DA-HN clade (DA-HN), all EMMPs of YI-LS clade (YI-LS), all EMMPs of DE-JP clade (DE-JP), and all EMMPs of ZA-MG clade (ZA-MG) based on species richness. All correlation coefficients are significant at $P < 0.01$.

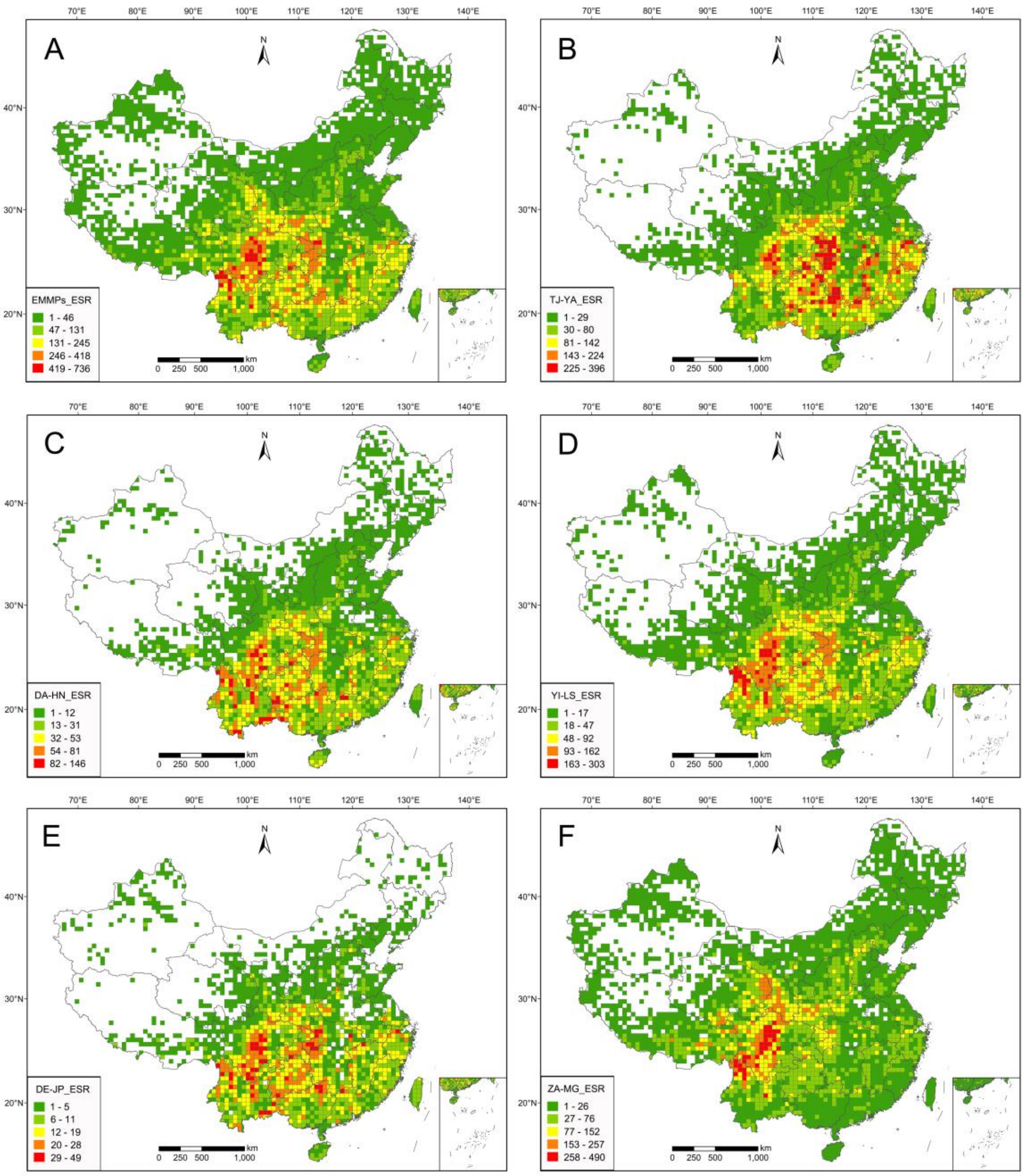

Figure S6. Spatial distribution patterns of species richness (SR) for different endemic plant categories: (A) endemic EMMPs of 24 ethnic minorities, (B) endemic EMMPs of TJ-YA clade, (C) endemic EMMPs of DA-HN clade, (D) endemic EMMPs of YI-LS clade, (E) endemic EMMPs of DE-JP clade, (F) endemic EMMPs of ZA-MG clade.

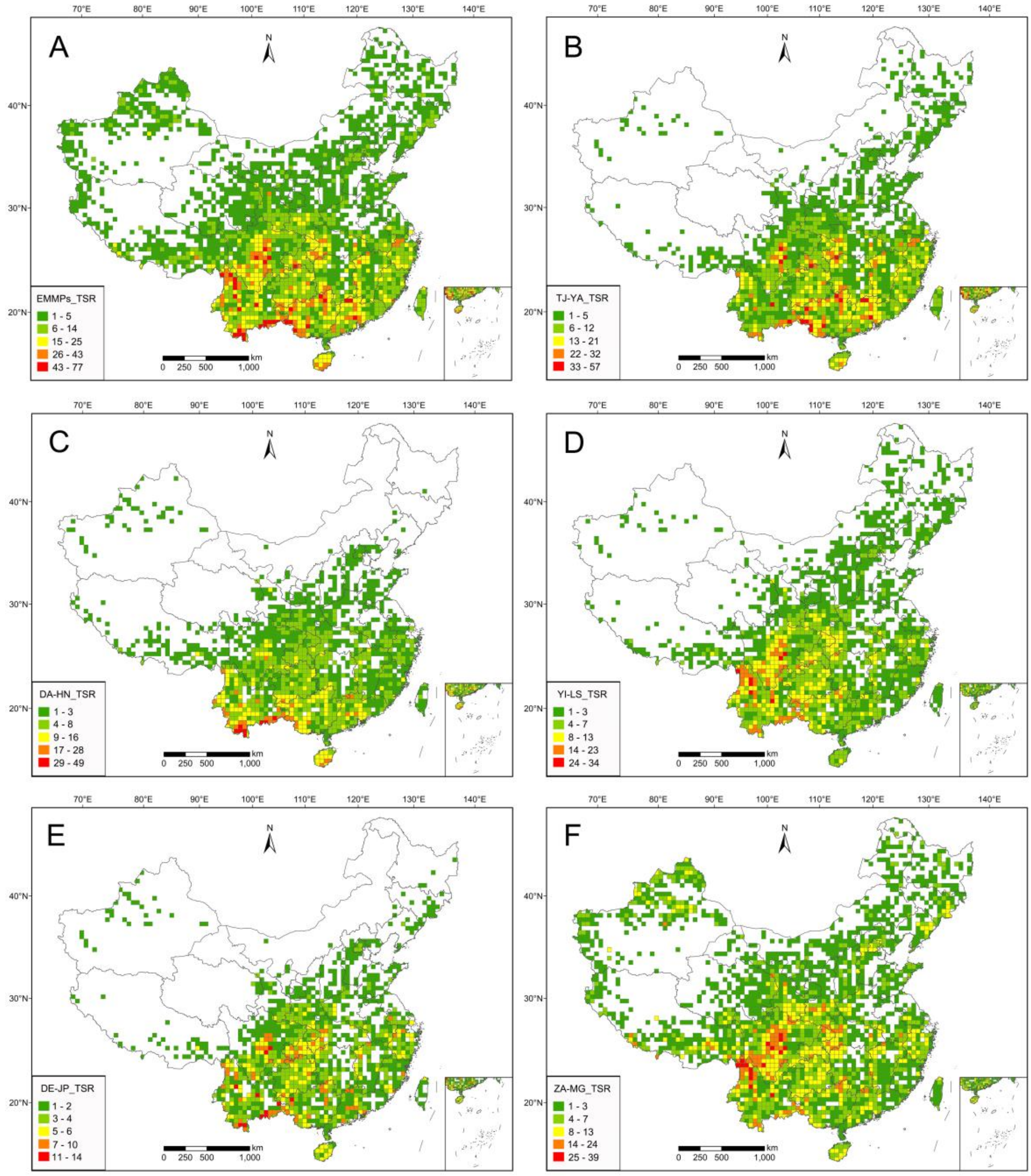

Figure S7. Spatial distribution patterns of species richness (SR) for different threatened plant categories: (A) threatened EMMPs of 24 ethnic minorities, (B) threatened EMMPs of TJ-YA clade, (C) threatened EMMPs of DA-HN clade, (D) threatened EMMPs of YI-LS clade, (E) threatened EMMPs of DE-JP clade, (F) threatened EMMPs of ZA-MG clade.

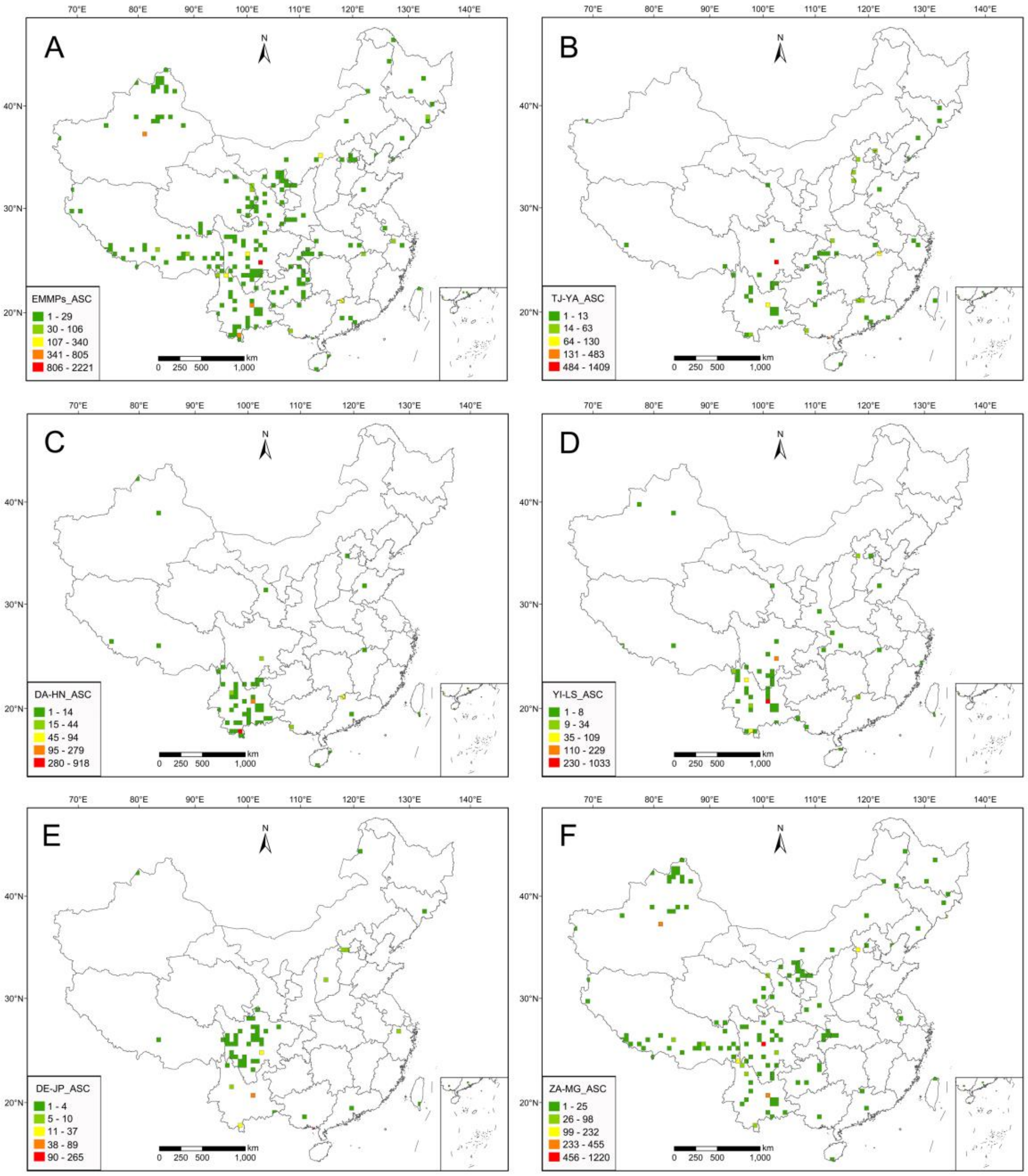

Figure S8. Spatial distribution patterns of species complementarity (SC) for different plant categories: (A) all EMMPs of 24 ethnic minorities, (B) all EMMPs of TJ-YA clade, (C) all EMMPs of DA-HN clade, (D) all EMMPs of YI-LS clade, (E) all EMMPs of DE-JP clade, (F) all EMMPs of ZA-MG clade.

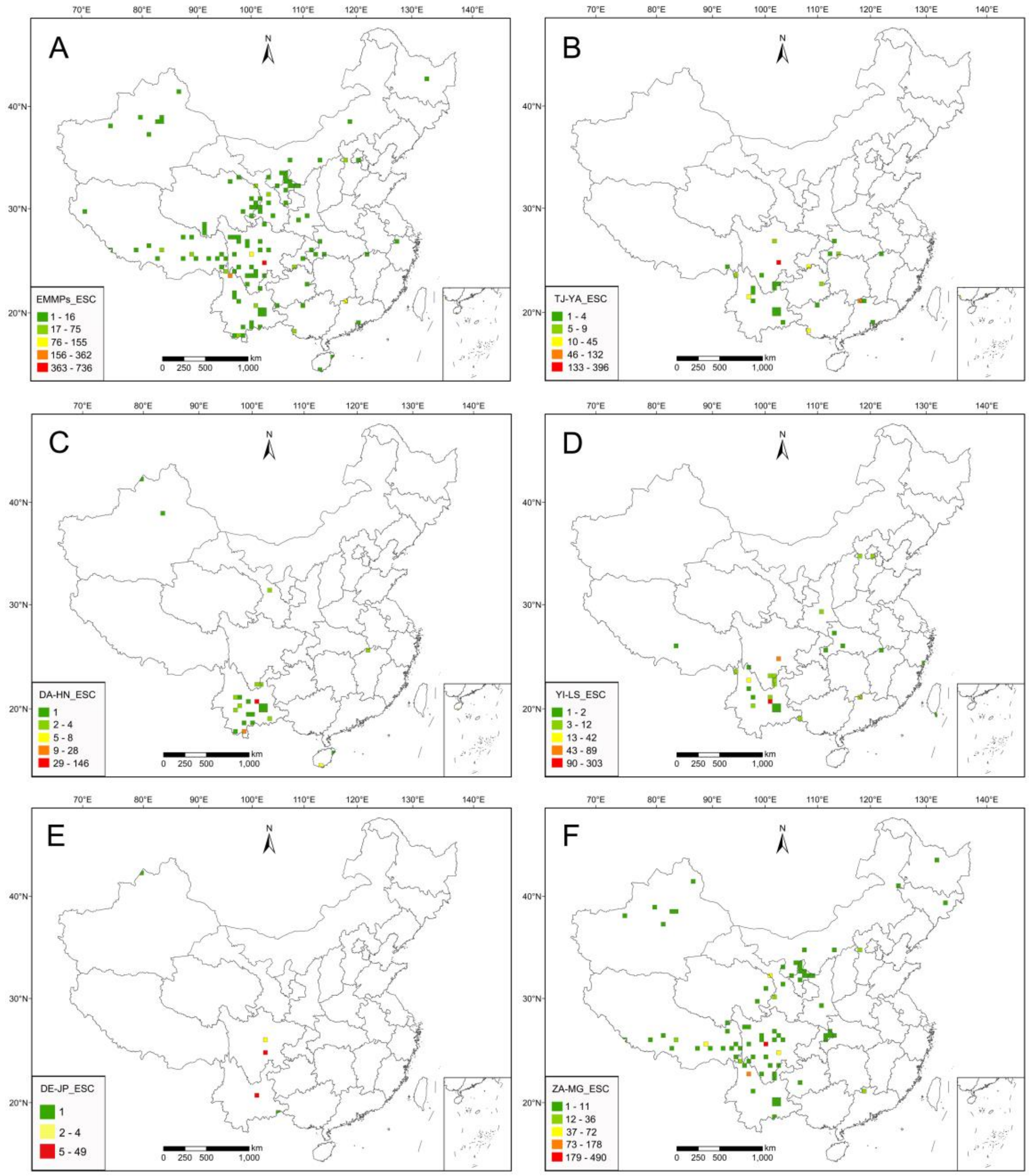

Figure S9. Spatial distribution patterns of species complementarity (SC) for different endemic plant categories: (A) endemic EMMPs of 24 ethnic minorities, (B) endemic EMMPs of TJ-YA clade, (C) endemic EMMPs of DA-HN clade, (D) endemic EMMPs of YI-LS clade, (E) endemic EMMPs of DE-JP clade, (F) endemic EMMPs of ZA-MG clade.

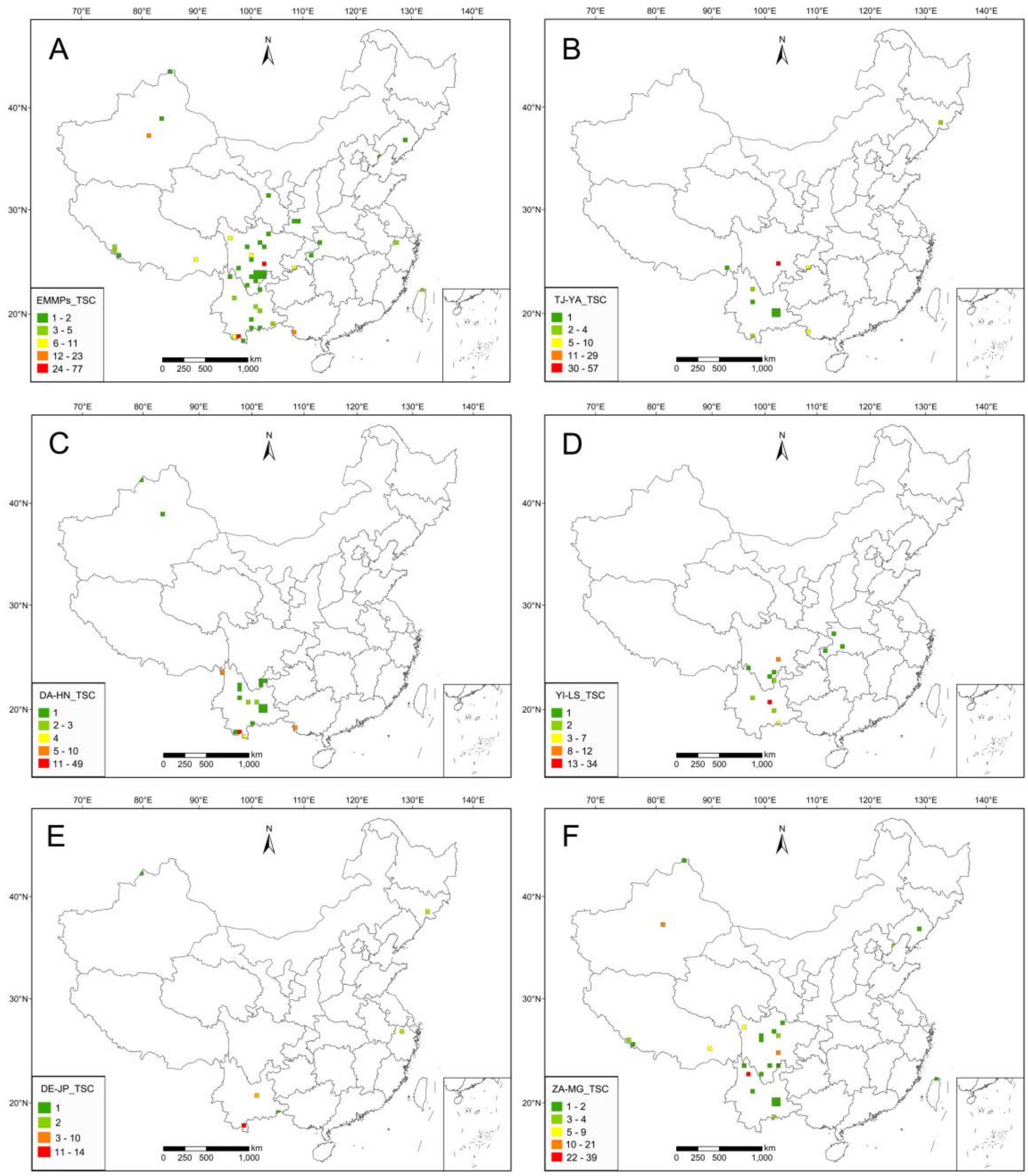

Figure S10. Spatial distribution patterns of species complementarity (SC) for different threatened plant categories: (A) threatened EMMPs of 24 ethnic minorities, (B) threatened EMMPs of TJ-YA clade, (C) threatened EMMPs of DA-HN clade, (D) threatened EMMPs of YI-LS clade, (E) threatened EMMPs of DE-JP clade, (F) threatened EMMPs of ZA-MG clade.

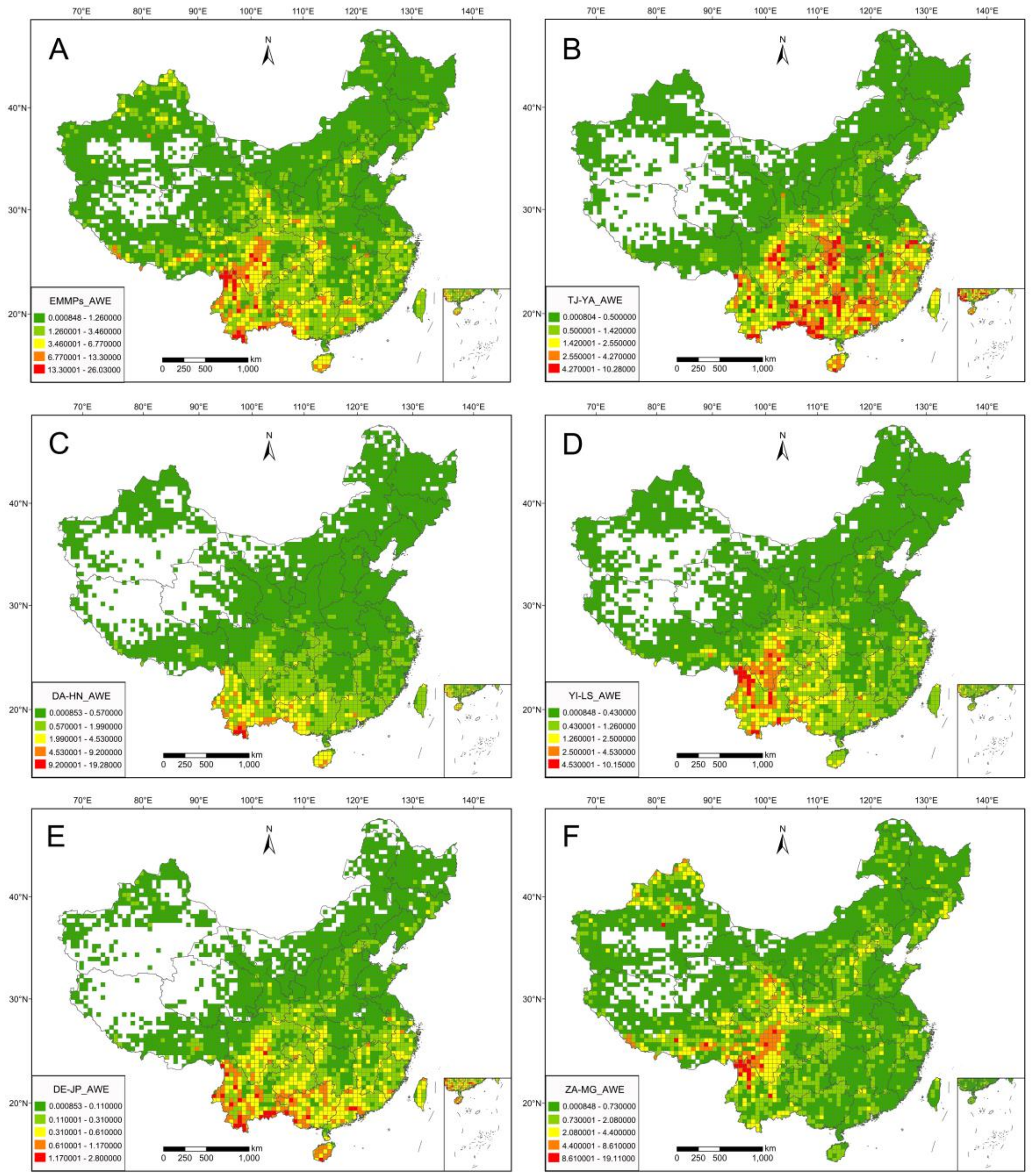

Figure S11. Spatial distribution patterns of weighted endemism (WE) for different plant categories: (A) all EMMPs of 24 ethnic minorities, (B) all EMMPs of TJ-YA clade, (C) all EMMPs of DA-HN clade, (D) all EMMPs of YI-LS clade, (E) all EMMPs of DE-JP clade, (F) all EMMPs of ZA-MG clade.

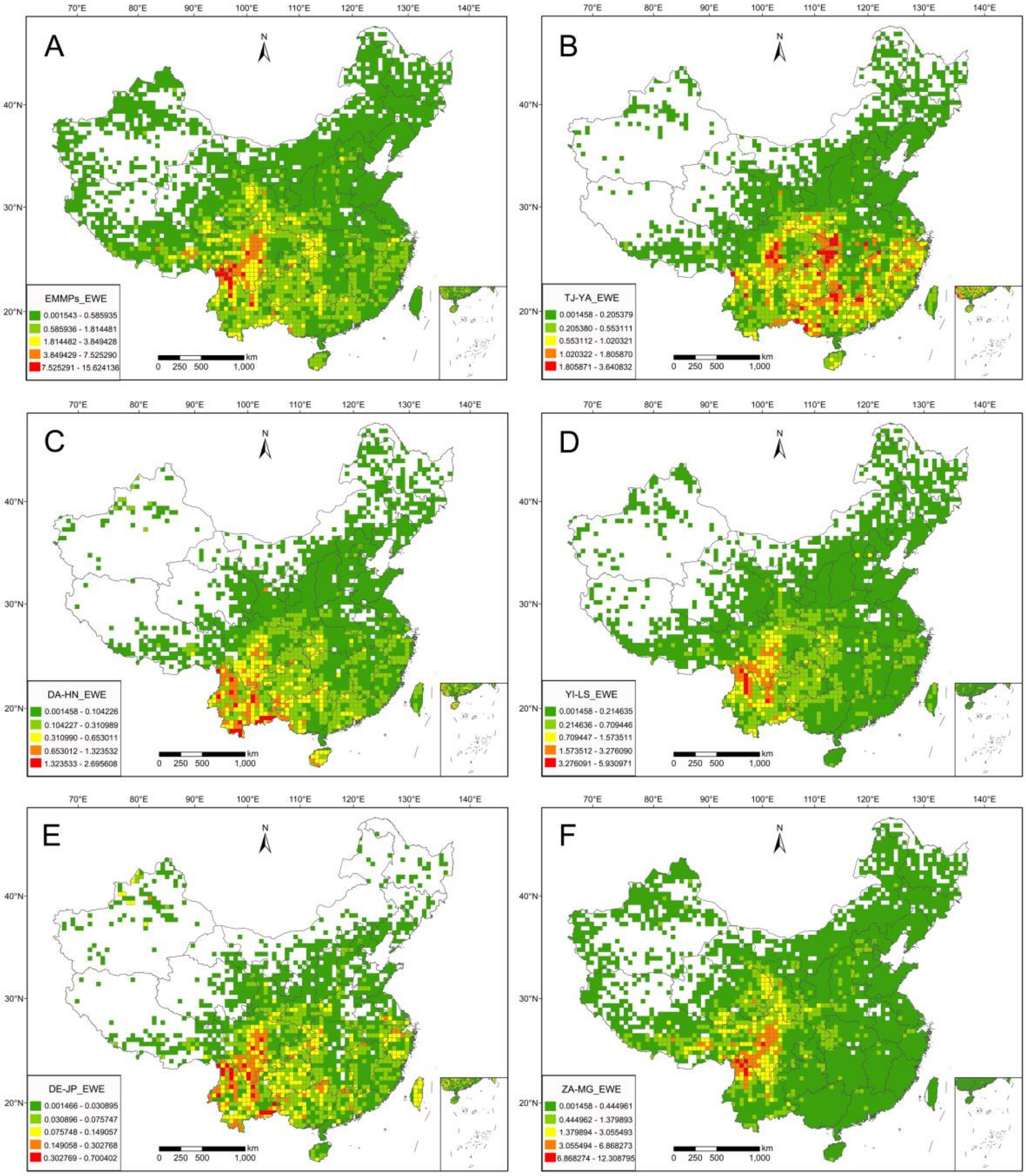

Figure S12. Spatial distribution patterns of weighted endemism (WE) for different endemic plant categories: (A) endemic EMMPs of 24 ethnic minorities, (B) endemic EMMPs of TJ-YA clade, (C) endemic EMMPs of DA-HN clade, (D) endemic EMMPs of YI-LS clade, (E) endemic EMMPs of DE-JP clade, (F) endemic EMMPs of ZA-MG clade.

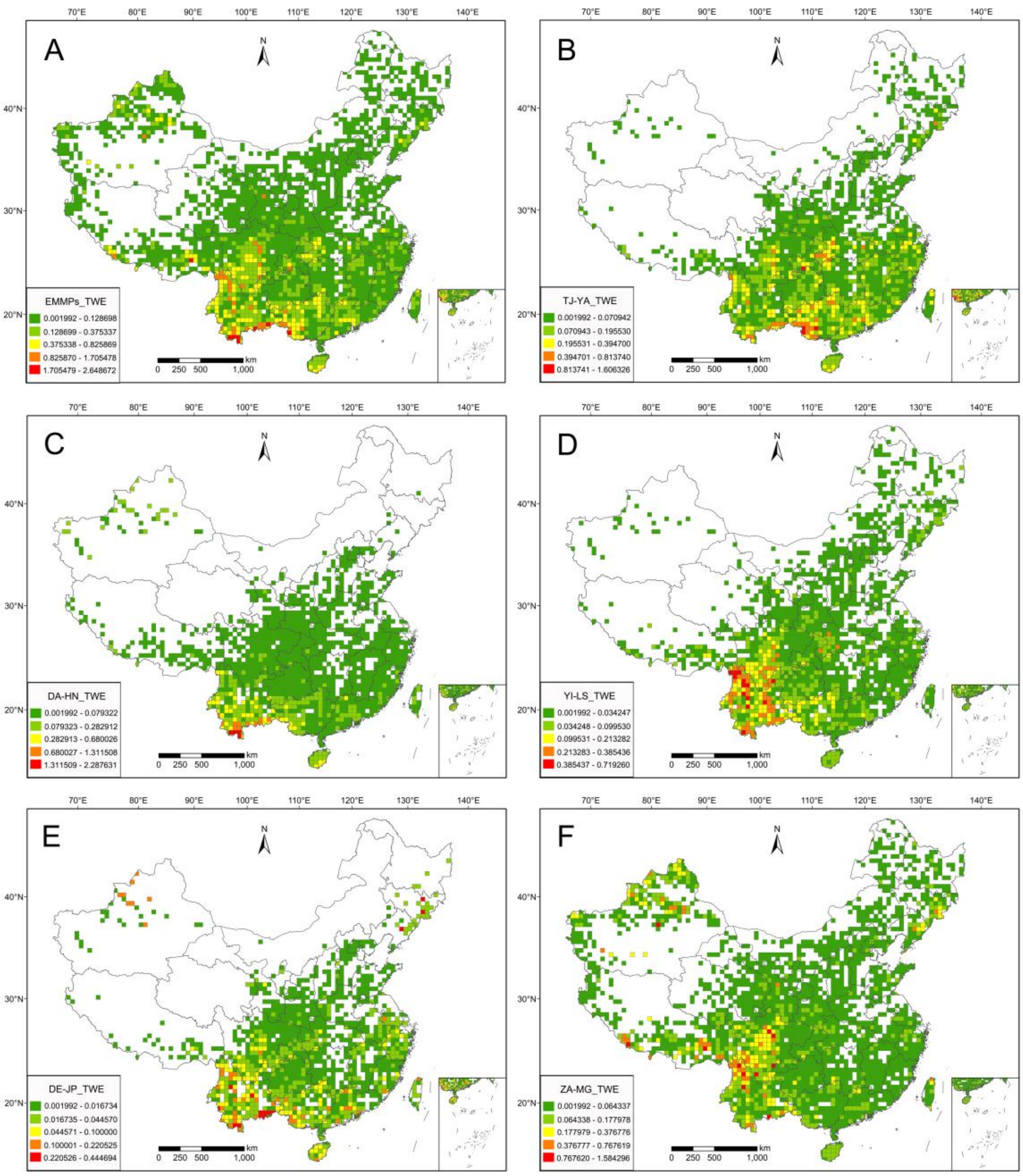

Figure S13. Spatial distribution patterns of weighted endemism (WE) for different threatened plant categories: (A) threatened EMMPs of 24 ethnic minorities, (B) threatened EMMPs of TJ-YA clade, (C) threatened EMMPs of DA-HN clade, (D) threatened EMMPs of YI-LS clade, (E) threatened EMMPs of DE-JP clade, (F) threatened EMMPs of ZA-MG clade.

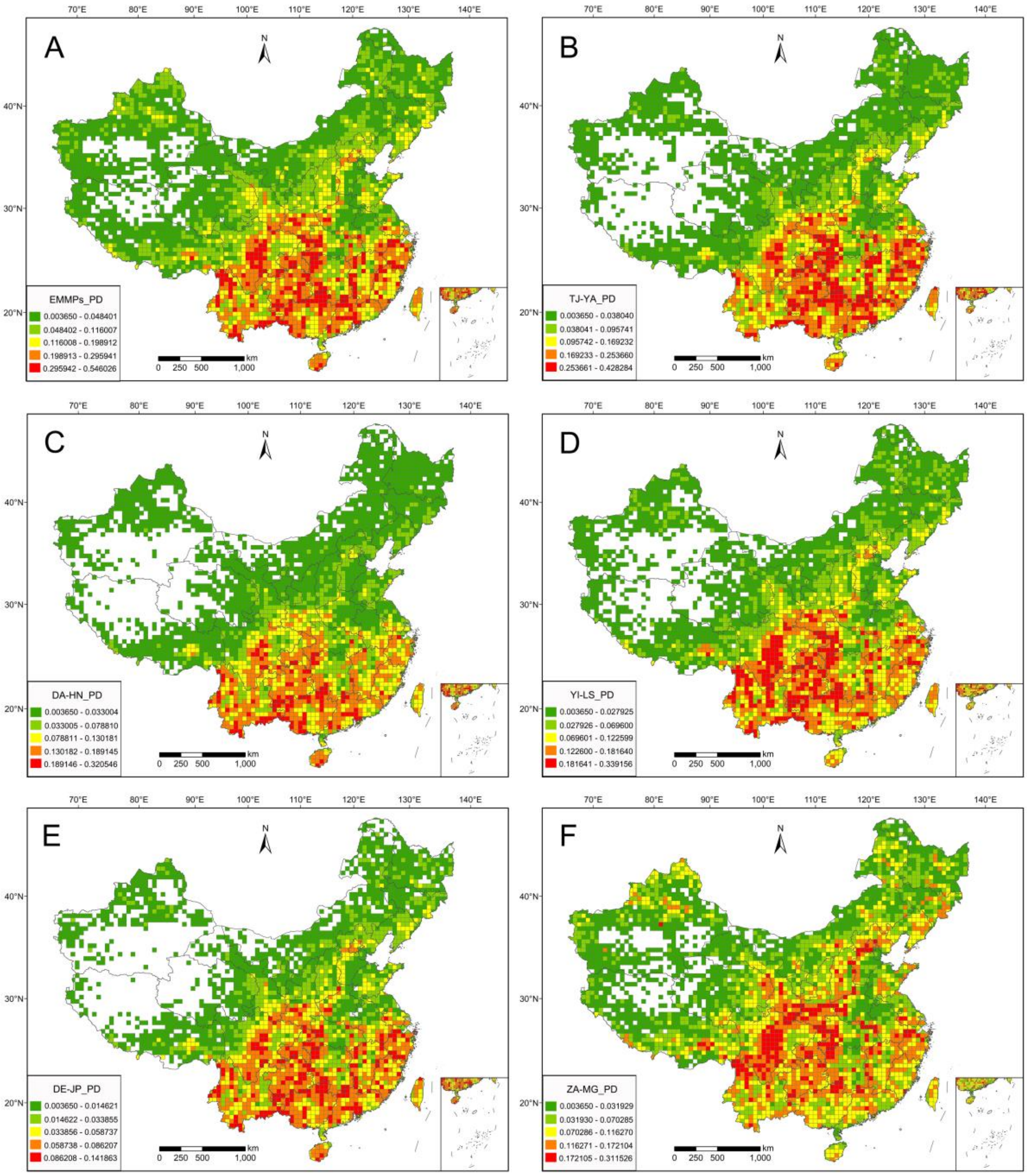

Figure S14. Spatial distribution patterns of phylogenetic diversity (PD) for different plant categories: (A) all EMMPs of 24 ethnic minorities, (B) all EMMPs of TJ-YA clade, (C) all EMMPs of DA-HN clade, (D) all EMMPs of YI-LS clade, (E) all EMMPs of DE-JP clade, (F) all EMMPs of ZA-MG clade.

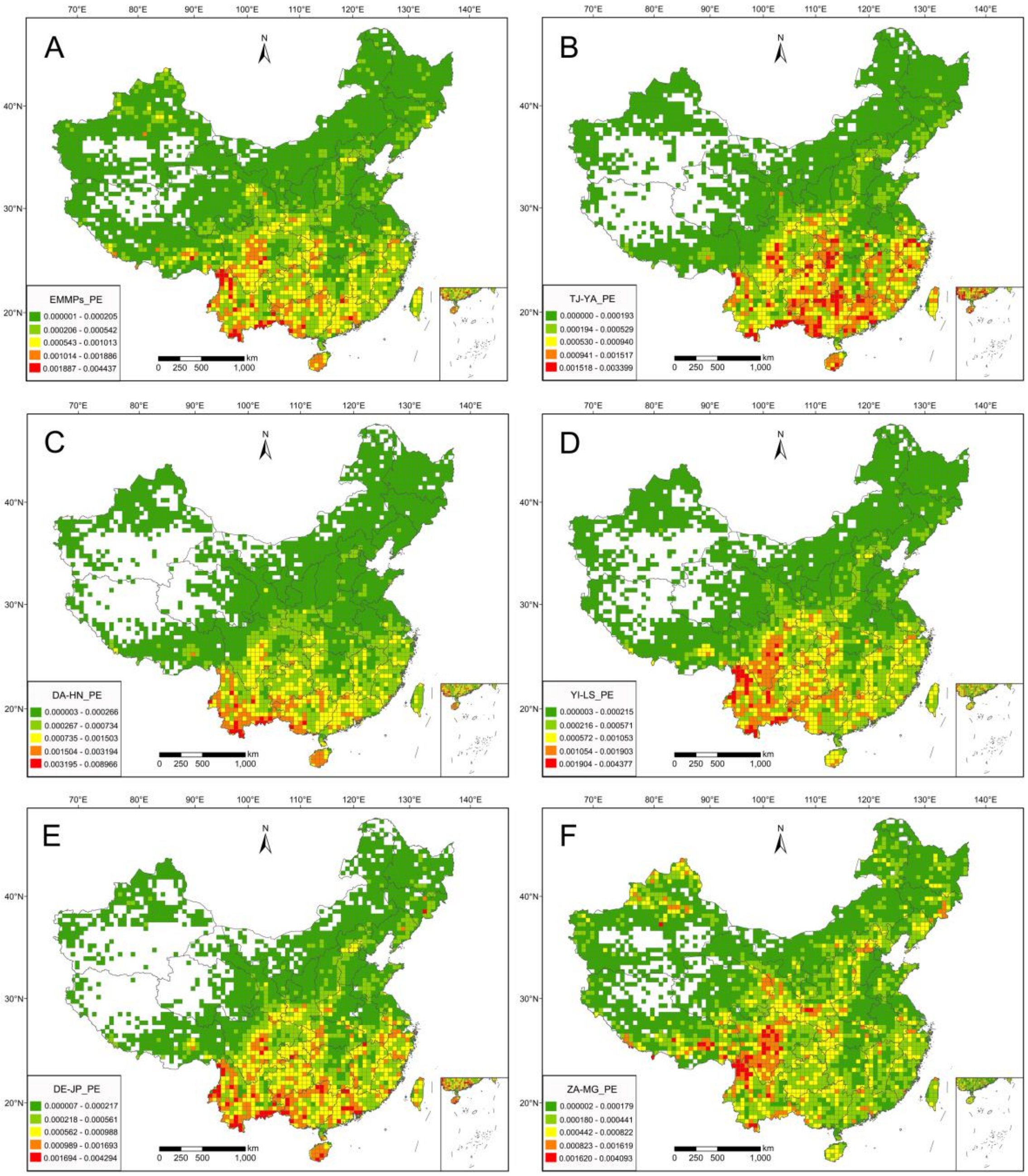

Figure S15. Spatial distribution patterns of phylogenetic endemism (PE) for different plant categories: (A) all EMMPs of 24 ethnic minorities, (B) all EMMPs of TJ-YA clade, (C) all EMMPs of DA-HN clade, (D) all EMMPs of YI-LS clade, (E) all EMMPs of DE-JP clade, (F) all EMMPs of ZA-MG clade.

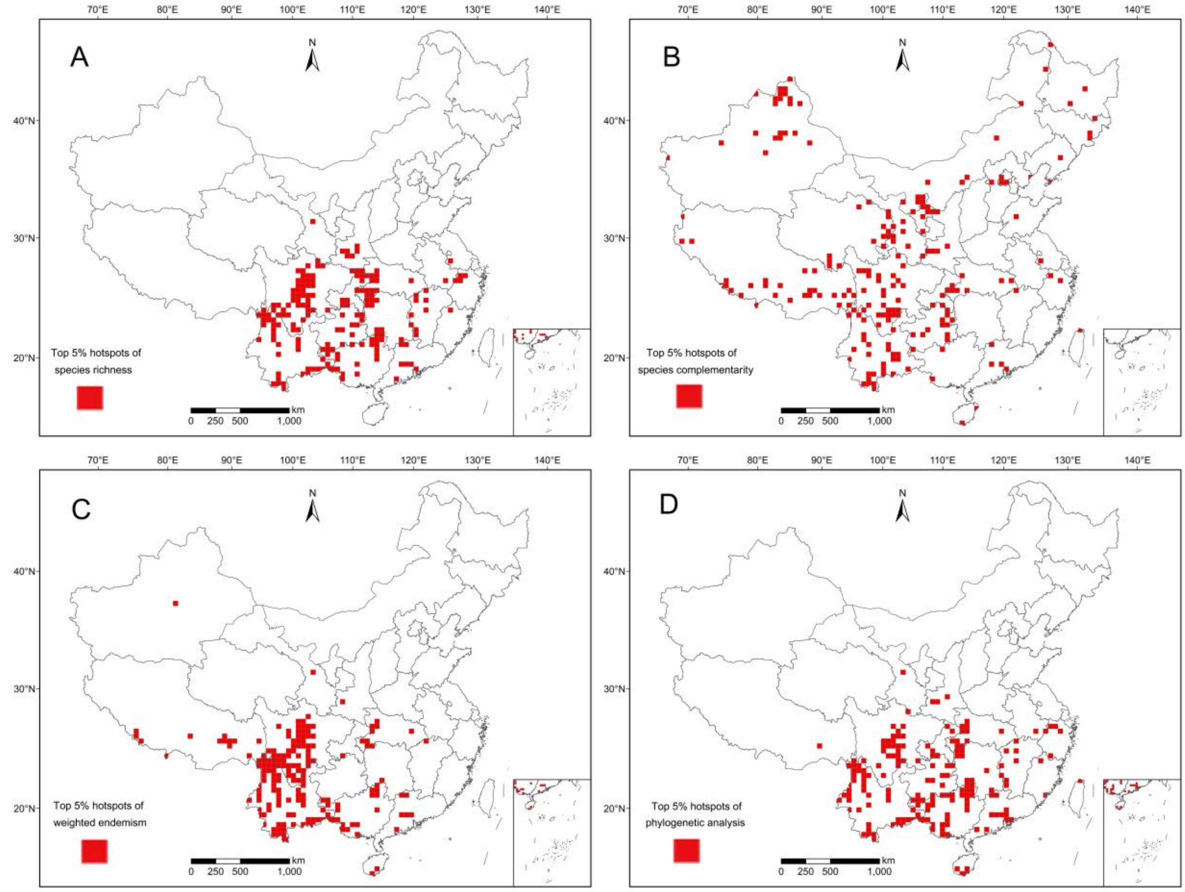

Figure S16. Spatial distribution patterns of top 5% hotspots of different algorithms. (A) Top 5% hotspots of species richness. (B) Top 5% hotspots of species complementarity. (C) Top 5% hotspots of weighted endemism. (D) Top 5% hotspots of phylogenetic analysis.

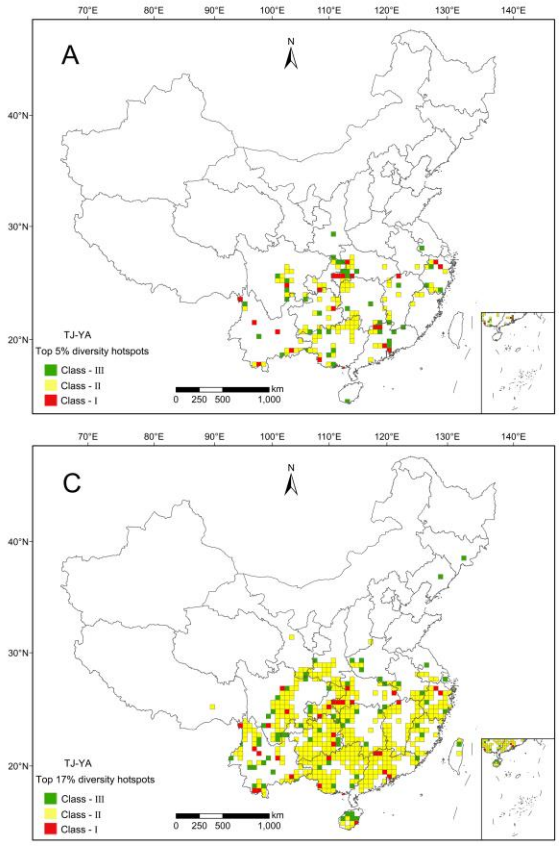

Figure S17. Spatial distribution patterns of diversity hotspots for all EMMPs of TJ-YA clade at thresholds of top 5% (A), top 10% (B), top17 % (C), and top 30% (D).

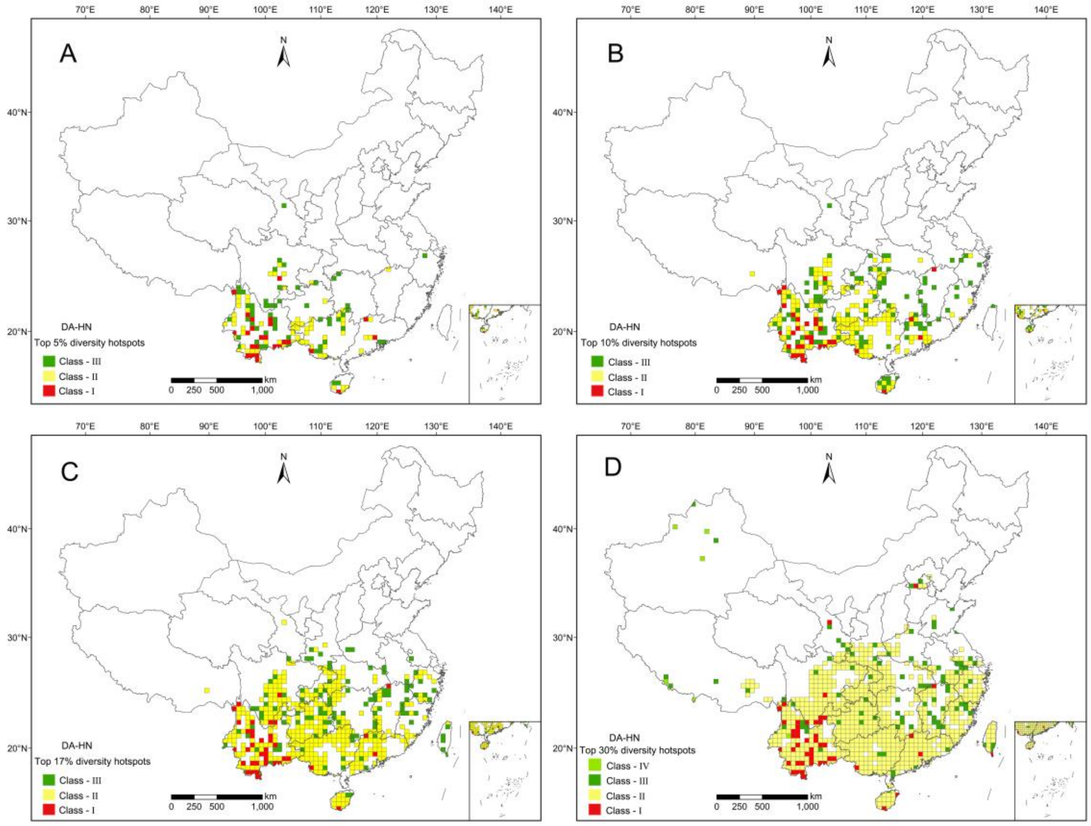

Figure S18. Spatial distribution patterns of diversity hotspots for all EMMPs of DA-HN clade at thresholds of top 5% (A), top 10% (B), top17 % (C), and top 30% (D).

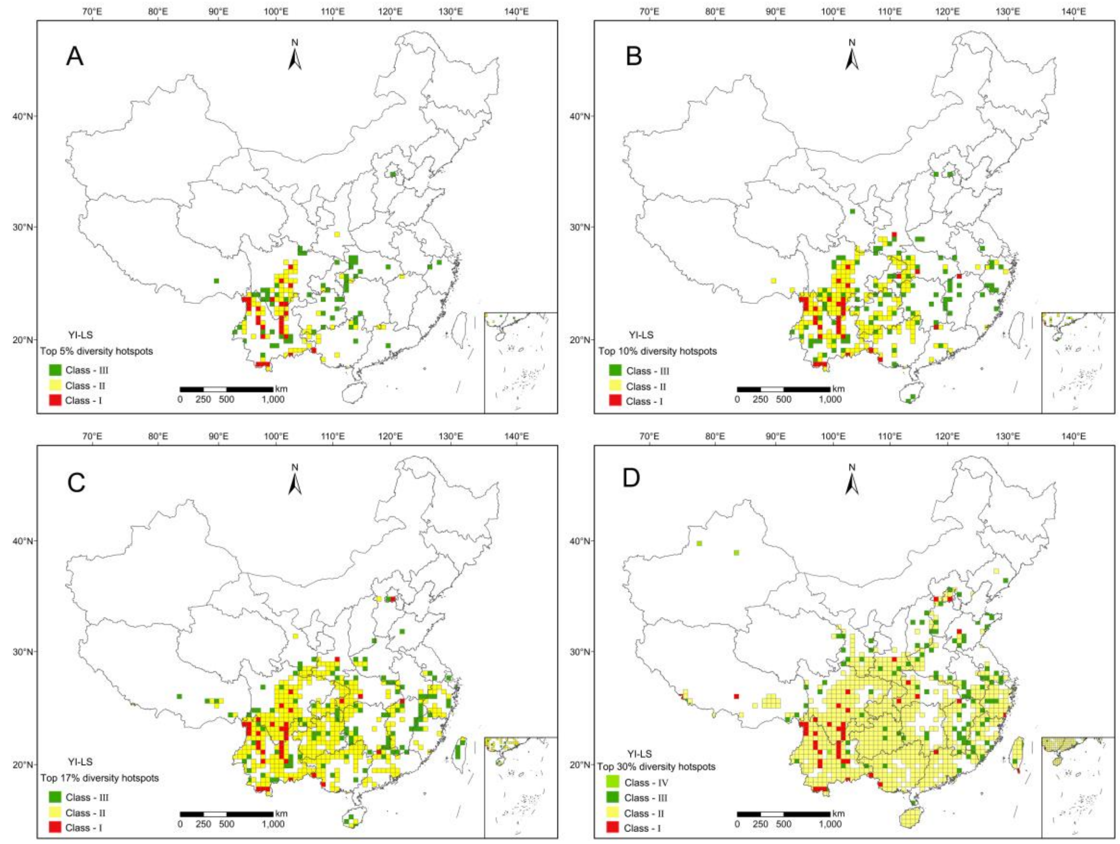

Figure S19. Spatial distribution patterns of diversity hotspots for all EMMPs of YI-LS clade at thresholds of top 5% (A), top 10% (B), top17 % (C), and top 30% (D).

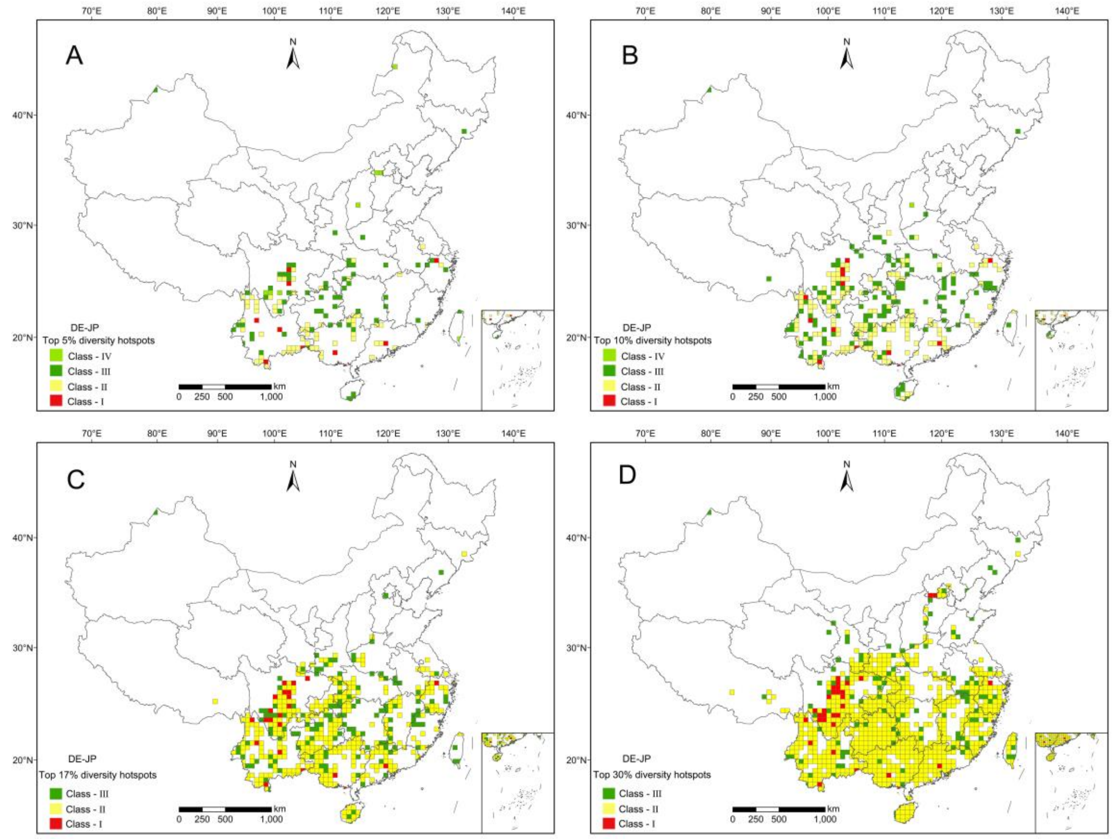

Figure S20. Spatial distribution patterns of diversity hotspots for all EMMPs of DE-JP clade at thresholds of top 5% (A), top 10% (B), top17 % (C), and top 30% (D).

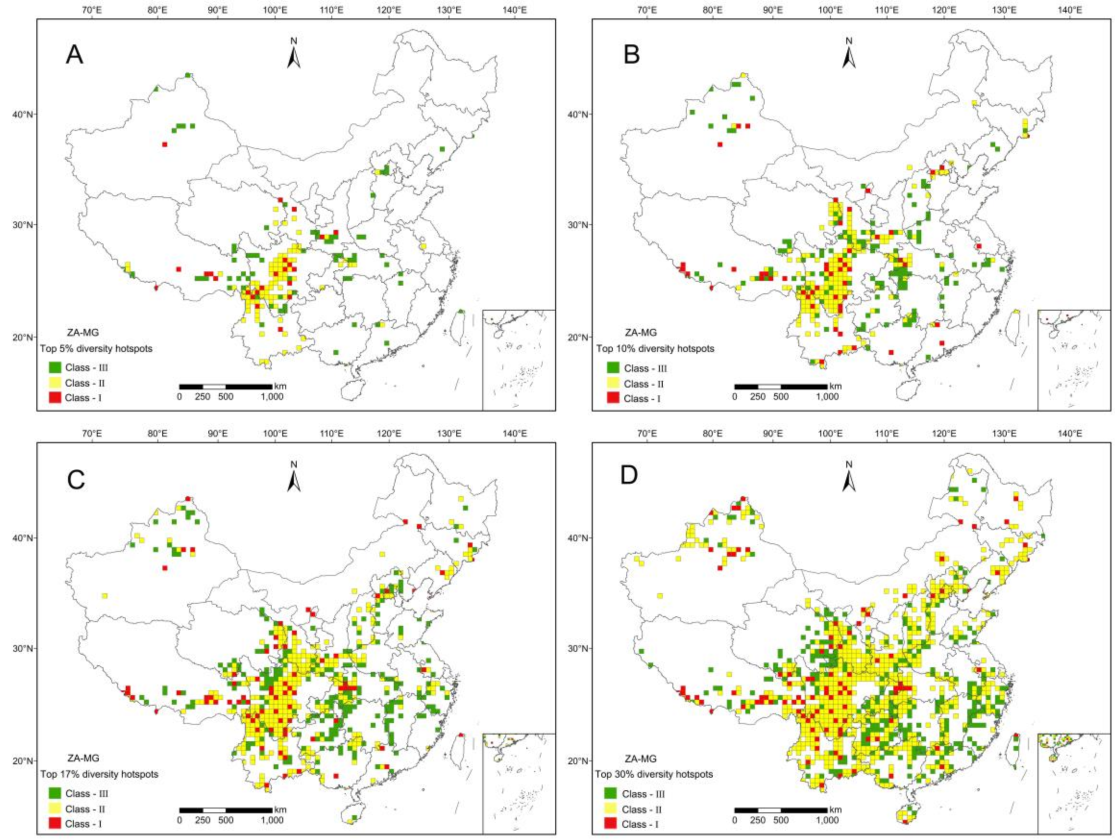

Figure S21. Spatial distribution patterns of diversity hotspots for all EMMPs of ZA-MG clade at thresholds of top 5% (A), top 10% (B), top17 % (C), and top 30% (D).

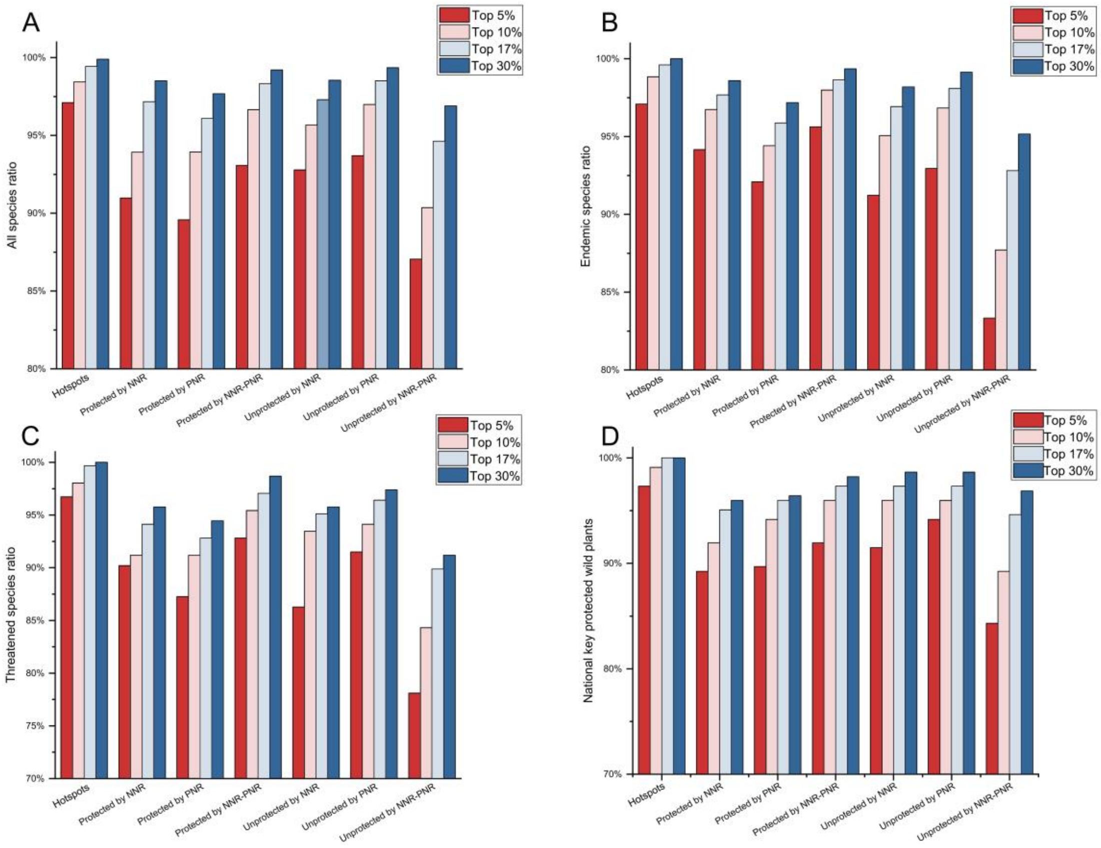

Figure S22. Statistics of conservation effectiveness of all EMMPs (A), endemic EMMPs(B), threatened EMMPs (C), and protected EMMPs (D) at thresholds of top 5%, top 10%, top17 %, and top 30%.

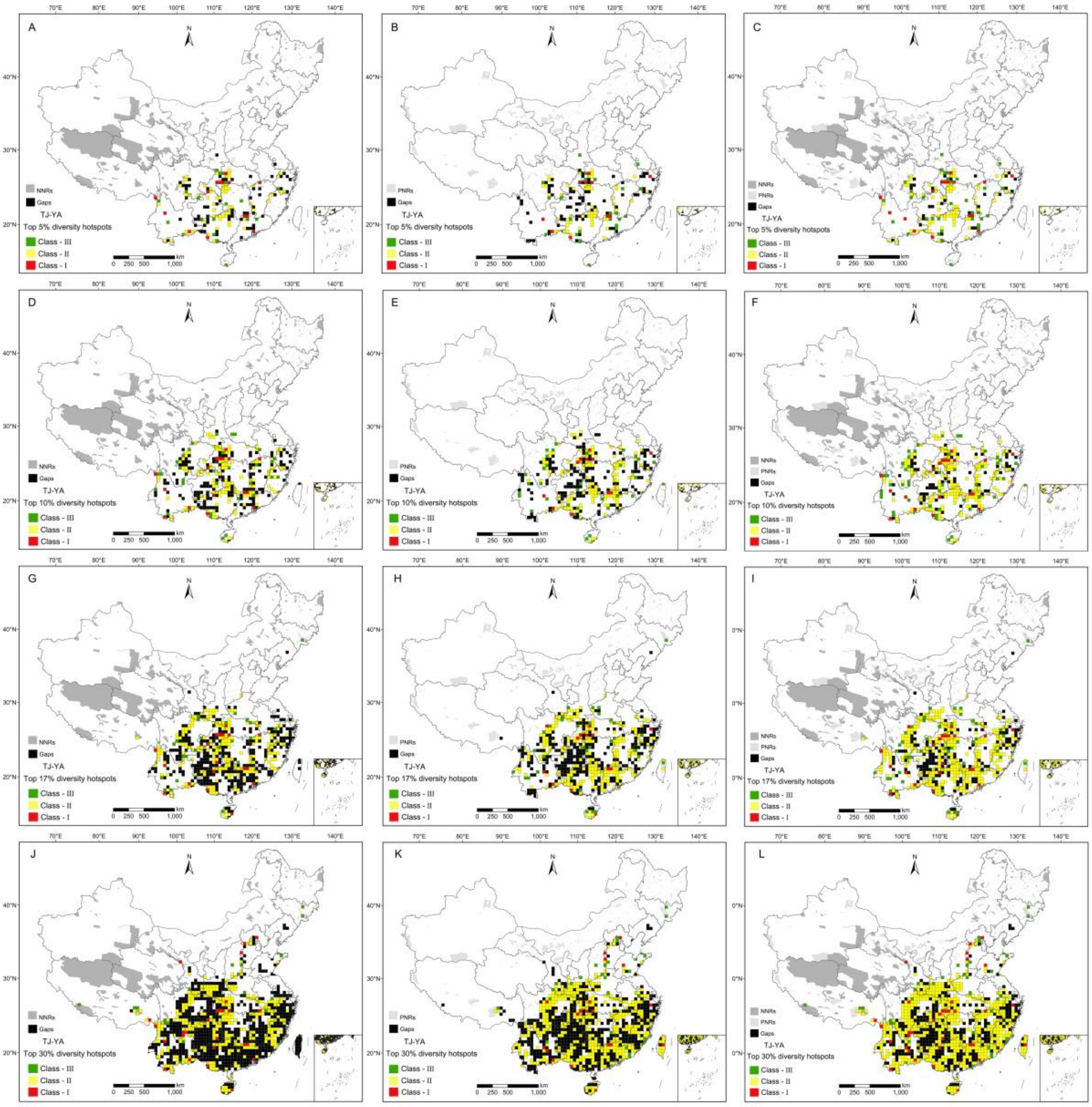

Figure S23. Spatial distribution patterns of conservation effectiveness and gaps for all EMMPs of TJ-YA clade based on four different thresholds. (A-C) Based on top 5% threshold, conservation effectiveness and gaps for national nature reserves (NNRs), provincial nature reserves (PNRs), and NNRs-PNRs, respectively. (D-F) Based on top 10% threshold, conservation effectiveness and gaps for NNRs, PNRs, and NNRs-PNRs, respectively. (G-I) Based on top 17% threshold, conservation effectiveness and gaps for NNRs, PNRs, and NNRs-PNRs, respectively. (J-L) Based on top 30% threshold, conservation effectiveness and gaps for NNRs, PNRs, and NNRs-PNRs, respectively.

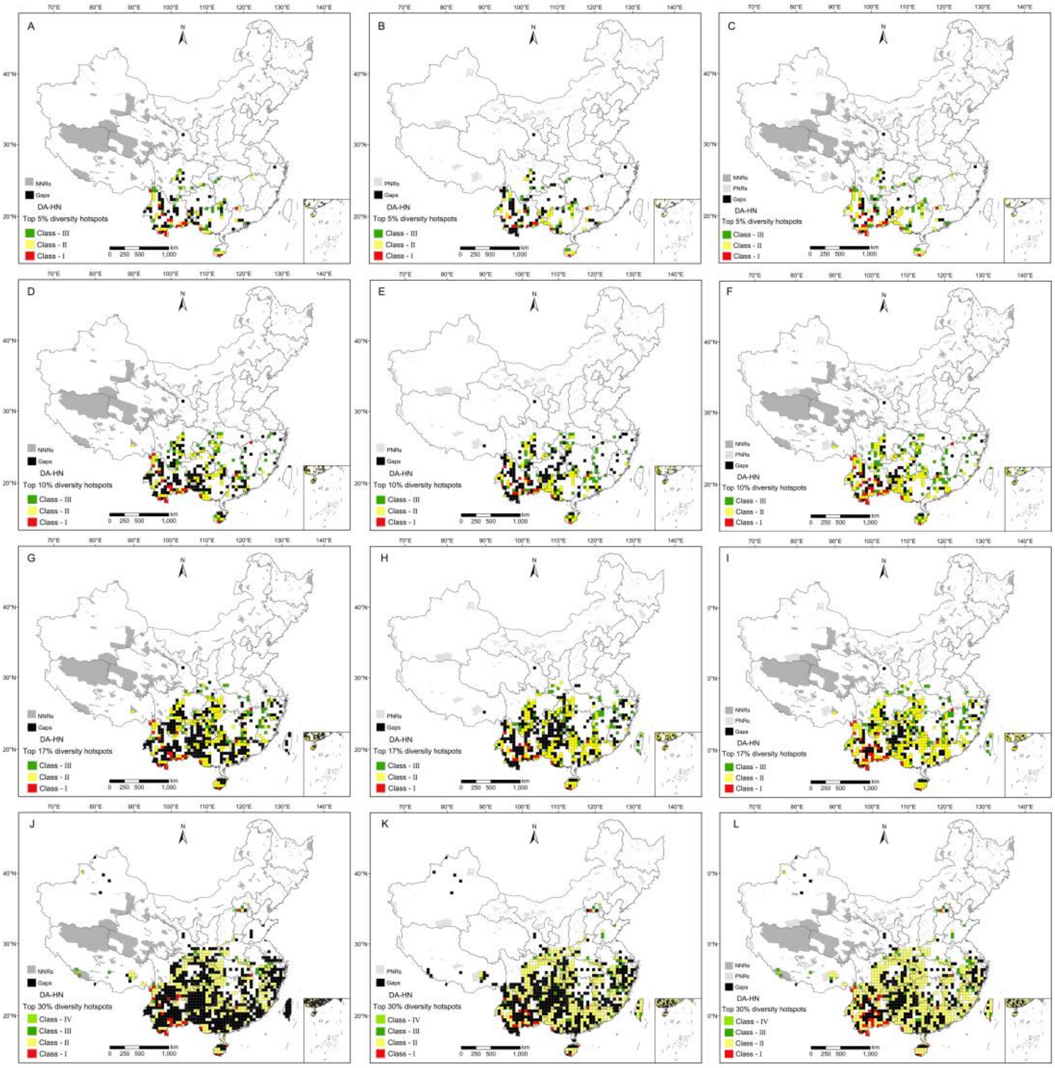

Figure S24. Spatial distribution patterns of conservation effectiveness and gaps for all EMMPs of DA-HN clade based on different four thresholds. (A-C) Based on top 5% threshold, conservation effectiveness and gaps for national nature reserves (NNRs), provincial nature reserves (PNRs), and NNRs-PNRs, respectively. (D-F) Based on top 10% threshold, conservation effectiveness and gaps for NNRs, PNRs, and NNRs-PNRs, respectively. (G-I) Based on top 17% threshold, conservation effectiveness and gaps for NNRs, PNRs, and NNRs-PNRs, respectively. (J-L) Based on top 30% threshold, conservation effectiveness and gaps for NNRs, PNRs, and NNRs-PNRs, respectively.

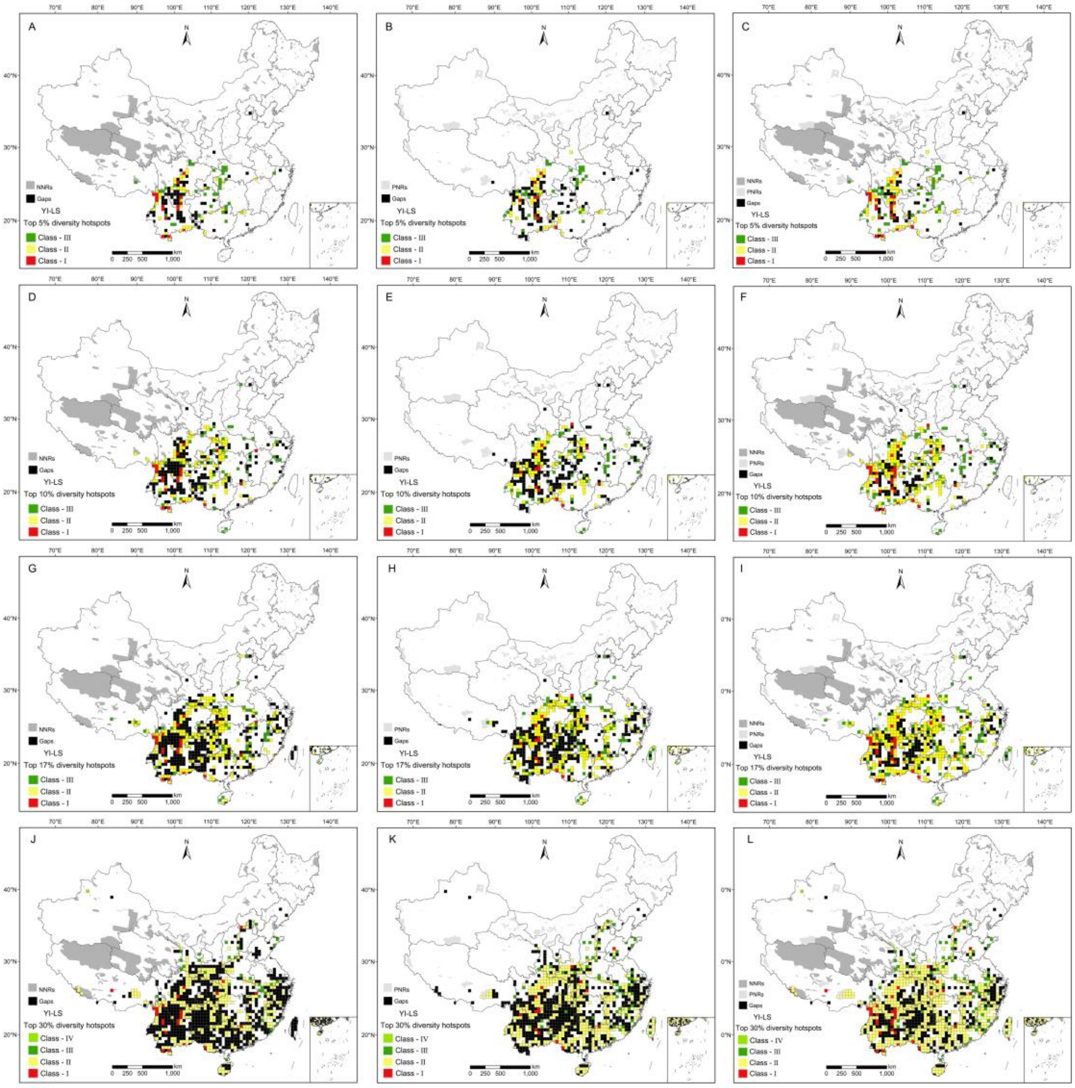

Figure S25. Spatial distribution patterns of conservation effectiveness and gaps for all EMMPs of YI-LS clade based on different four thresholds. (A-C) Based on top 5% threshold, conservation effectiveness and gaps for national nature reserves (NNRs), provincial nature reserves (PNRs), and NNRs-PNRs, respectively. (D-F) Based on top 10% threshold, conservation effectiveness and gaps for NNRs, PNRs, and NNRs-PNRs, respectively. (G-I) Based on top 17% threshold, conservation effectiveness and gaps for NNRs, PNRs, and NNRs-PNRs, respectively. (J-L) Based on top 30% threshold, conservation effectiveness and gaps for NNRs, PNRs, and NNRs-PNRs, respectively.

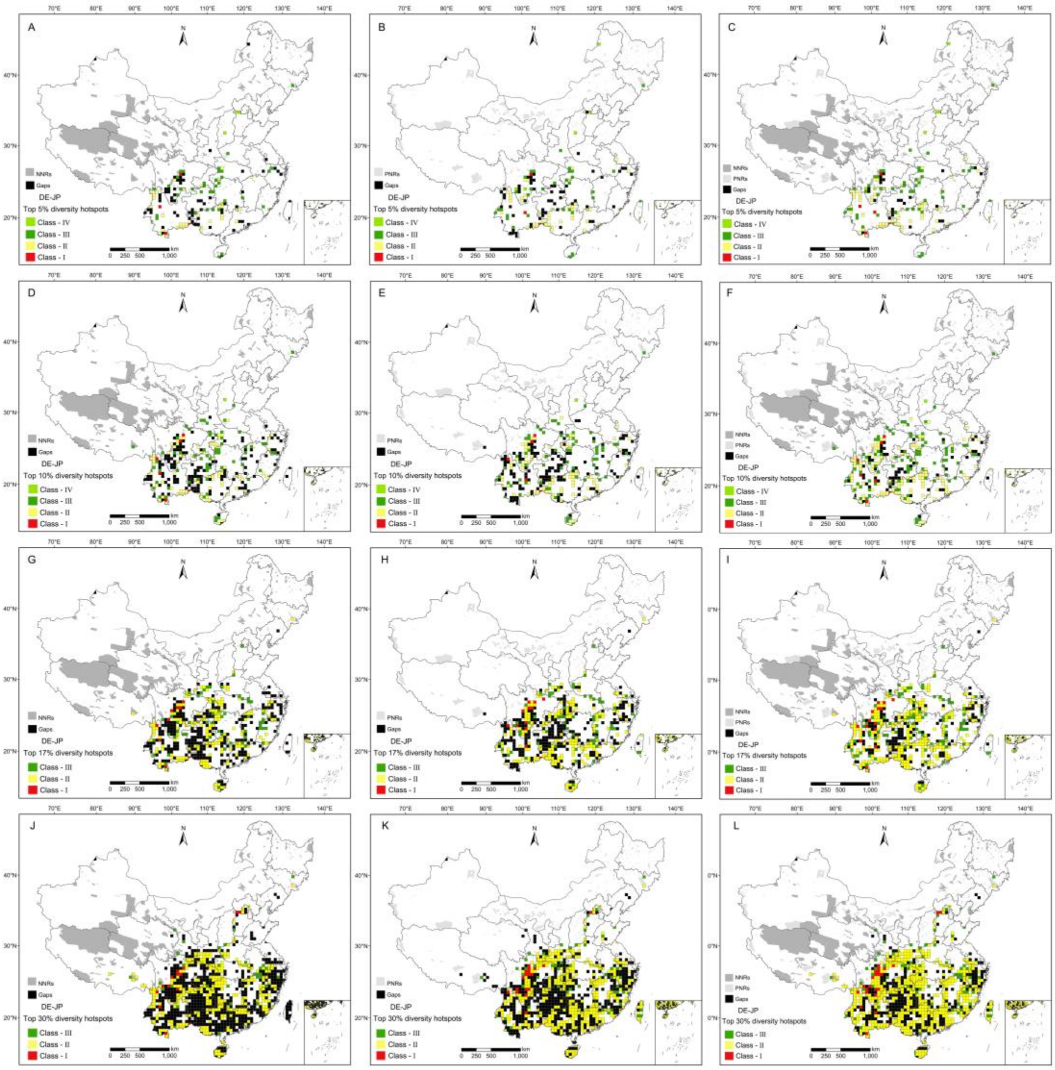

Figure S26. Spatial distribution patterns of conservation effectiveness and gaps for all EMMPs of DE-JP clade based on different four thresholds. (A-C) Based on top 5% threshold, conservation effectiveness and gaps for national nature reserves (NNRs), provincial nature reserves (PNRs), and NNRs-PNRs, respectively. (D-F) Based on top 10% threshold, conservation effectiveness and gaps for NNRs, PNRs, and NNRs-PNRs, respectively. (G-I) Based on top 17% threshold, conservation effectiveness and gaps for NNRs, PNRs, and NNRs-PNRs, respectively. (J-L) Based on top 30% threshold, conservation effectiveness and gaps for NNRs, PNRs, and NNRs-PNRs, respectively.

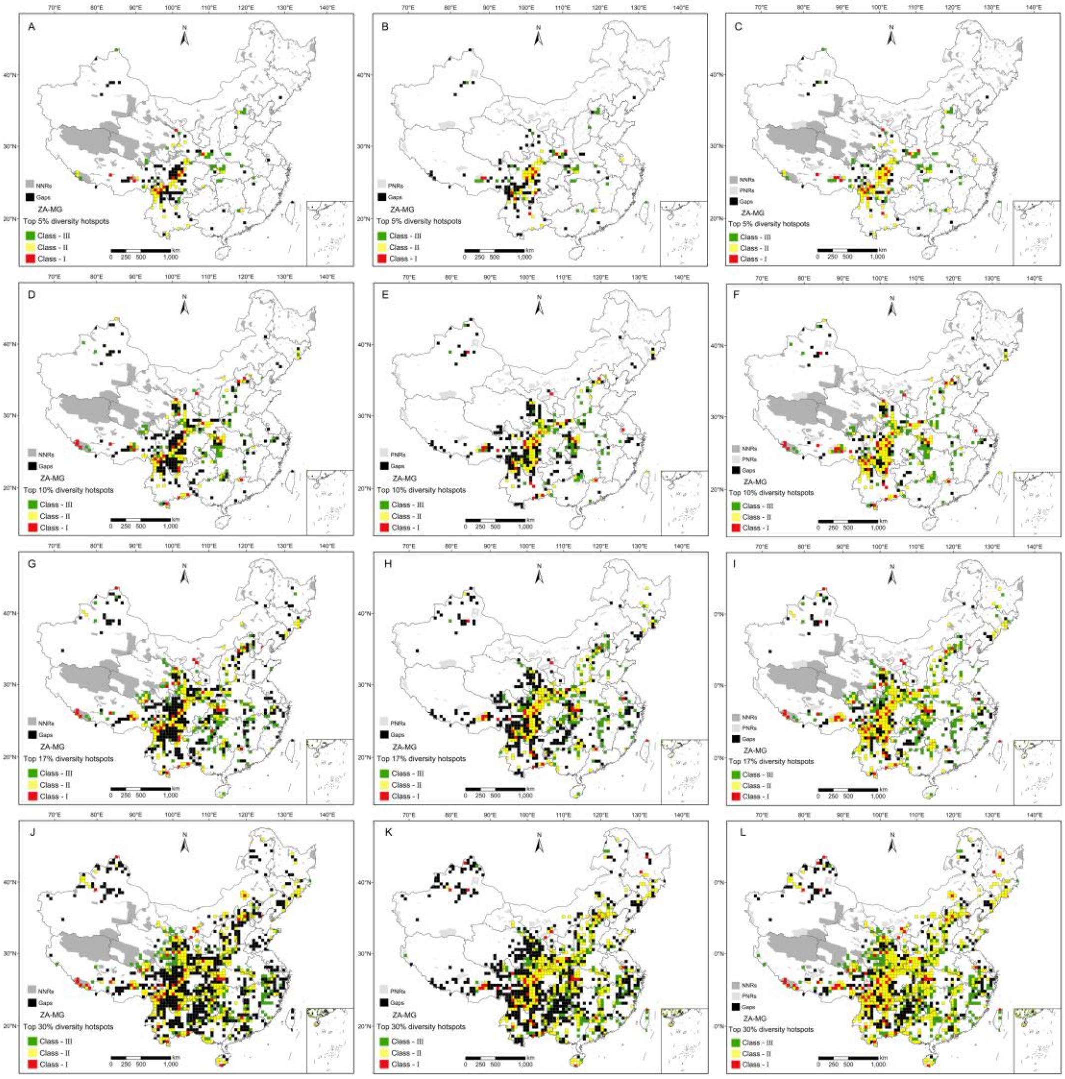

Figure S27. Spatial distribution patterns of conservation effectiveness and gaps for all EMMPs of ZA-MG clade based on different four thresholds. (A-C) Based on top 5% threshold, conservation effectiveness and gaps for national nature reserves (NNRs), provincial nature reserves (PNRs), and NNRs-PNRs, respectively. (D-F) Based on top 10% threshold, conservation effectiveness and gaps for NNRs, PNRs, and NNRs-PNRs, respectively. (G-I) Based on top 17% threshold, conservation effectiveness and gaps for NNRs, PNRs, and NNRs-PNRs, respectively. (J-L) Based on top 30% threshold, conservation effectiveness and gaps for NNRs, PNRs, and NNRs-PNRs, respectively.

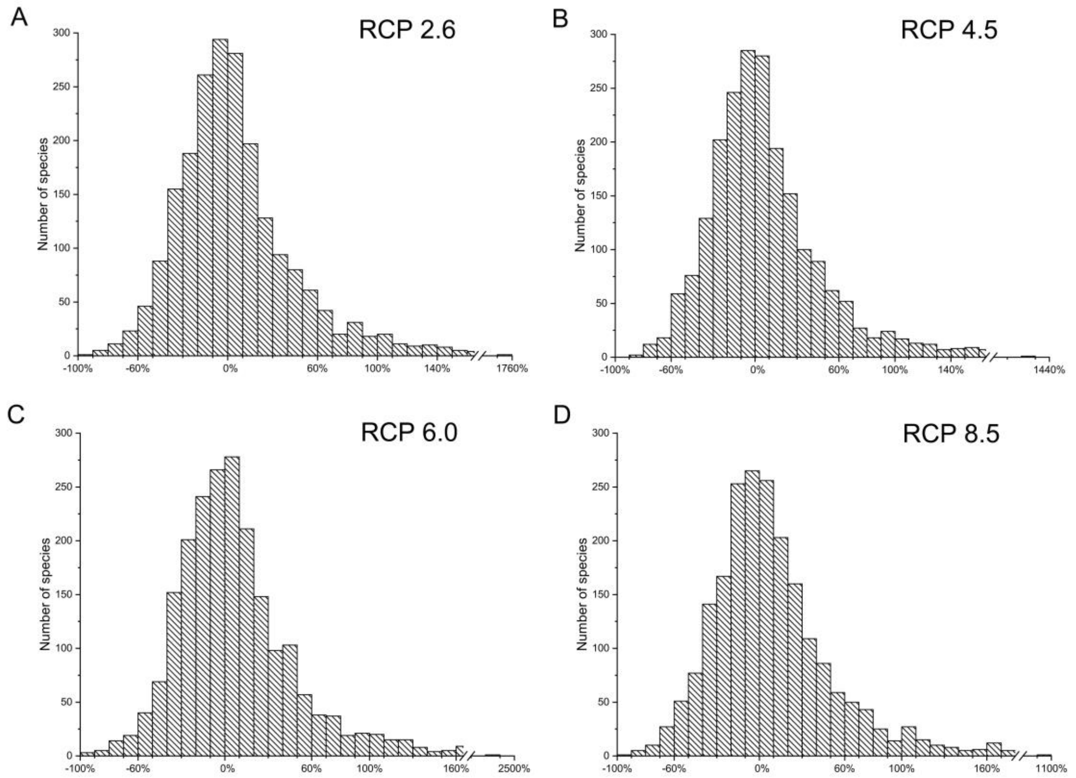

Figure S28. Changes in range sizes of endemic and threatened EMMPs under four emission scenarios: representative concentration pathway [RCP] 2.6 (A), RCP 4.5 (B), RCP 6.0 (C), and RCP 8.5 (D).

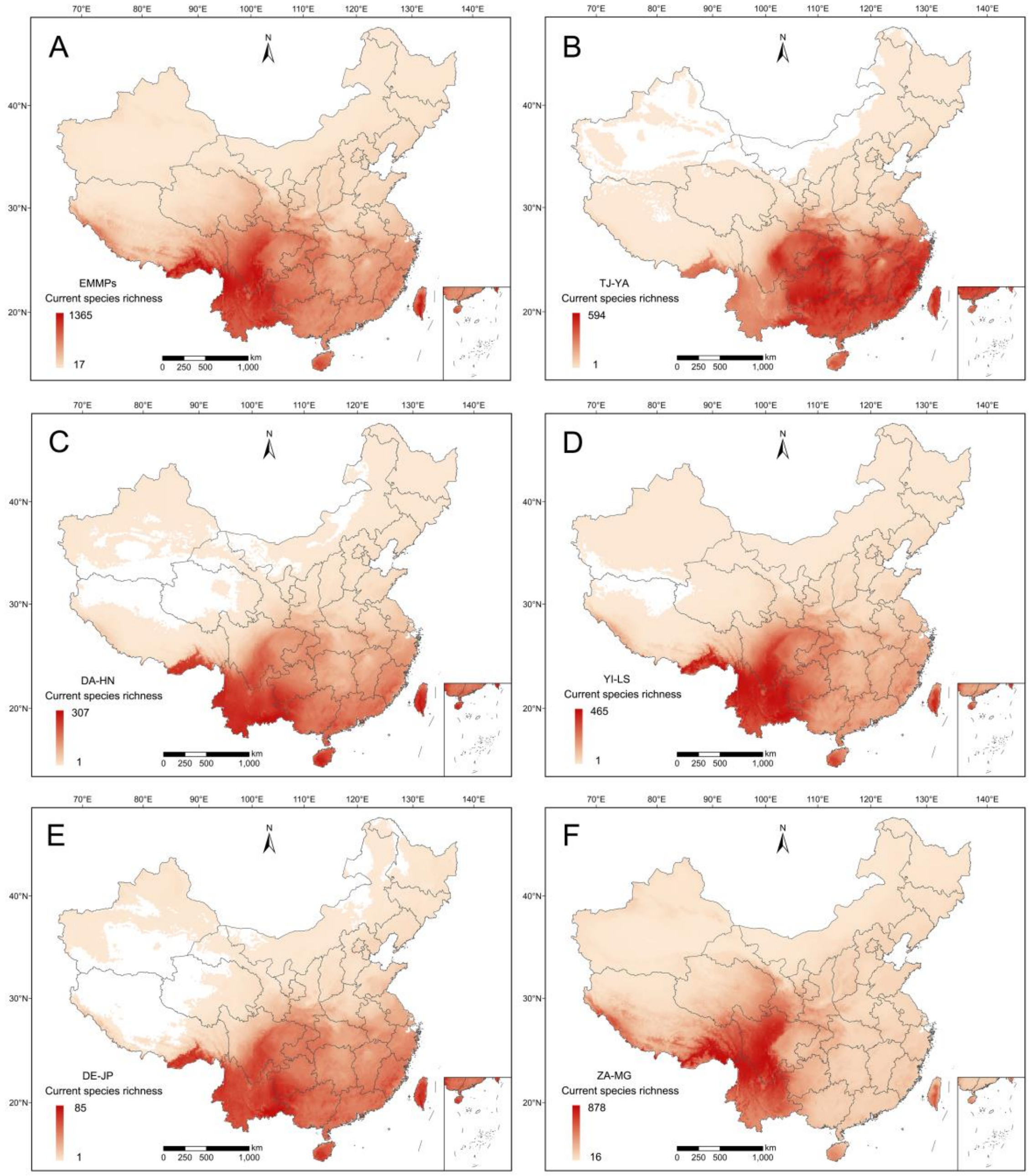

Figure S29. Spatial distribution patterns of species richness for different plant categories based on the results of MaxEnt. Time periods: near current (1960–1990). (A) Endemic and threatened EMMPs of 24 ethnic minorities. (B) Endemic and threatened EMMPs of TJ-YA clade. (C) Endemic and threatened EMMPs of DA-HN clade. (D) Endemic and threatened EMMPs of YI-LS clade. (E) Endemic and threatened EMMPs of DE-JP clade. (F) Endemic and threatened EMMPs of ZA-MG clade.

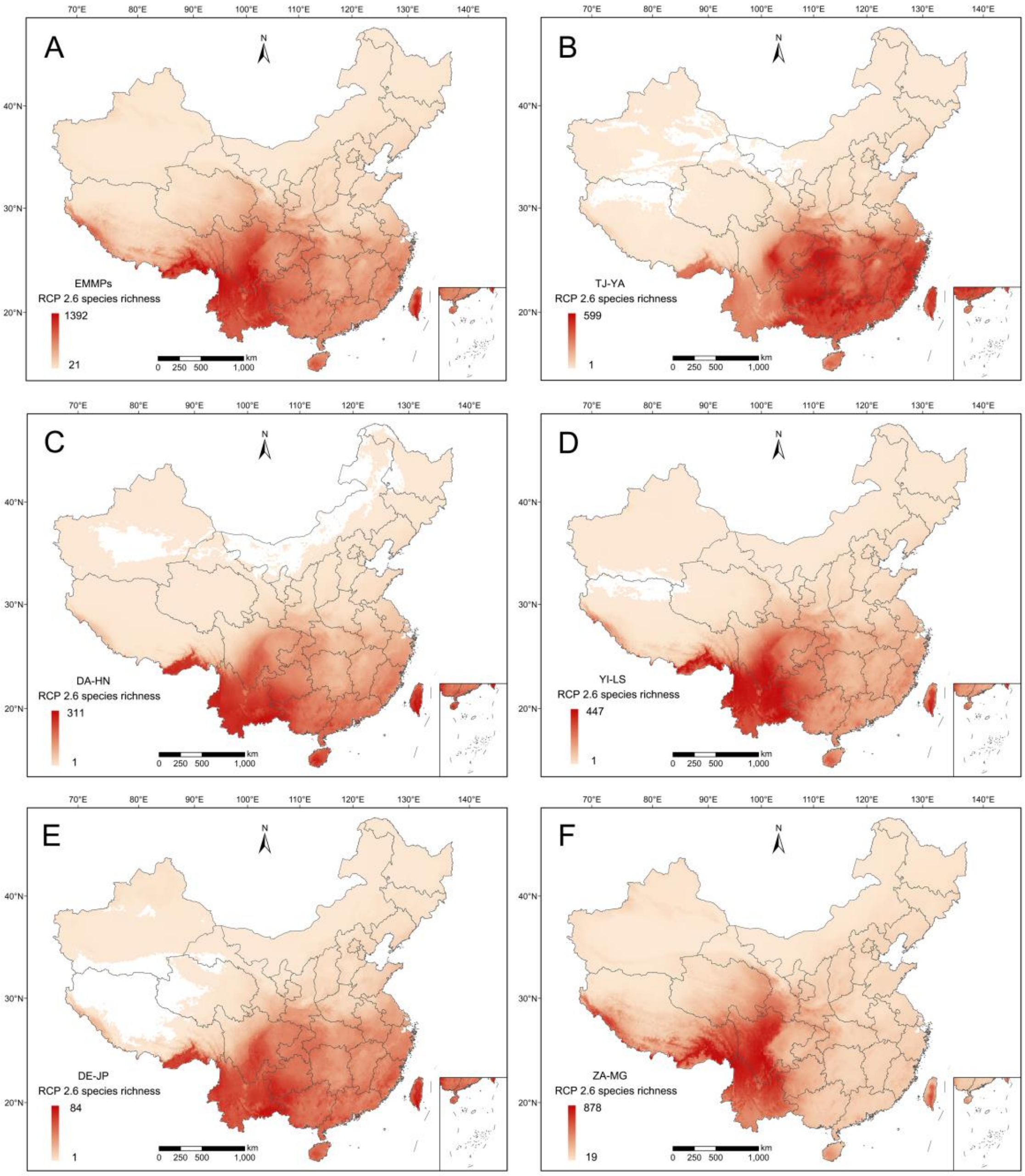

Figure S30. Spatial distribution patterns of species richness for different plant categories based on the results of MaxEnt. Time periods: future (2070). Emission scenarios: Representative concentration pathway (RCP) 2.6. (A) Endemic and threatened EMMPs of 24 ethnic minorities. (B) Endemic and threatened EMMPs of TJ-YA clade. (C) Endemic and threatened EMMPs of DA-HN clade. (D) Endemic and threatened EMMPs of YI-LS clade. (E) Endemic and threatened EMMPs of DE-JP clade. (F) Endemic and threatened EMMPs of ZA-MG clade.

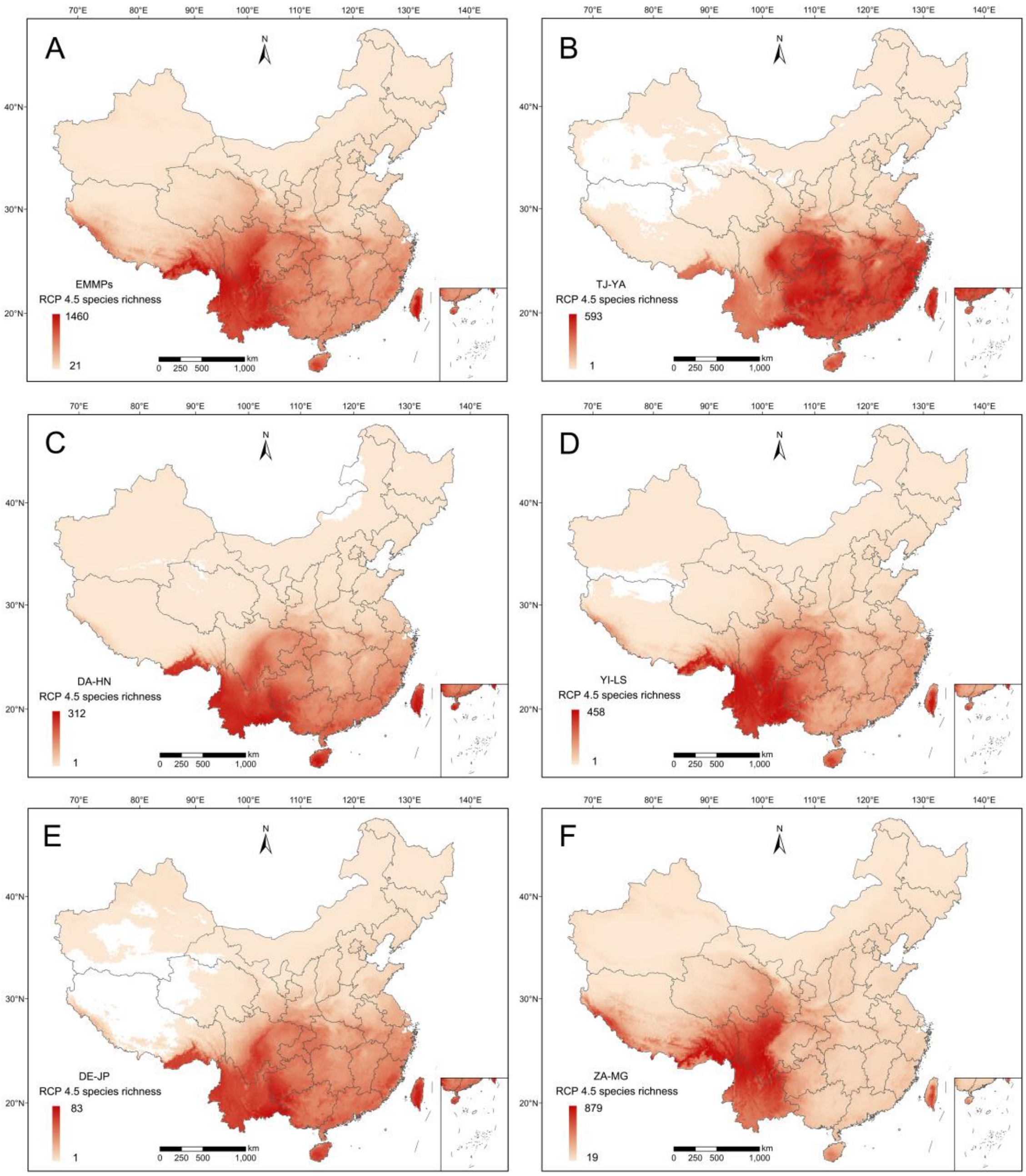

Figure S31. Spatial distribution patterns of species richness for different plant categories based on the results of MaxEnt. Time periods: future (2070). Emission scenarios: Representative concentration pathway (RCP) 4.5. (A) Endemic and threatened EMMPs of 24 ethnic minorities. (B) Endemic and threatened EMMPs of TJ-YA clade. (C) Endemic and threatened EMMPs of DA-HN clade. (D) Endemic and threatened EMMPs of YI-LS clade. (E) Endemic and threatened EMMPs of DE-JP clade. (F) Endemic and threatened EMMPs of ZA-MG clade.

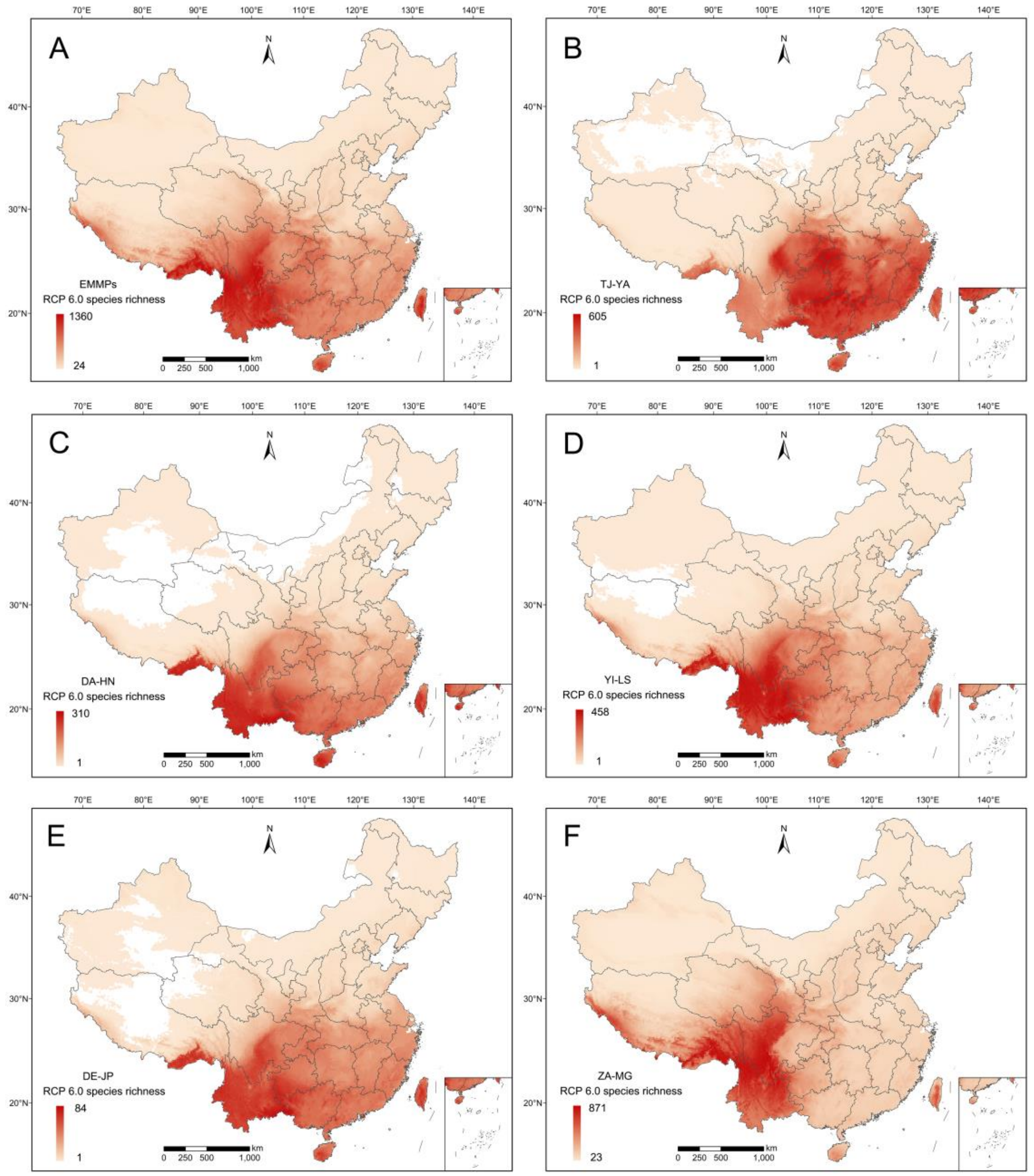

Figure S32. Spatial distribution patterns of species richness for different plant categories based on the results of MaxEnt. Time periods: future (2070). Emission scenarios: Representative concentration pathway (RCP) 6.0. (A) Endemic and threatened EMMPs of 24 ethnic minorities. (B) Endemic and threatened EMMPs of TJ-YA clade. (C) Endemic and threatened EMMPs of DA-HN clade. (D) Endemic and threatened EMMPs of YI-LS clade. (E) Endemic and threatened EMMPs of DE-JP clade. (F) Endemic and threatened EMMPs of ZA-MG clade.

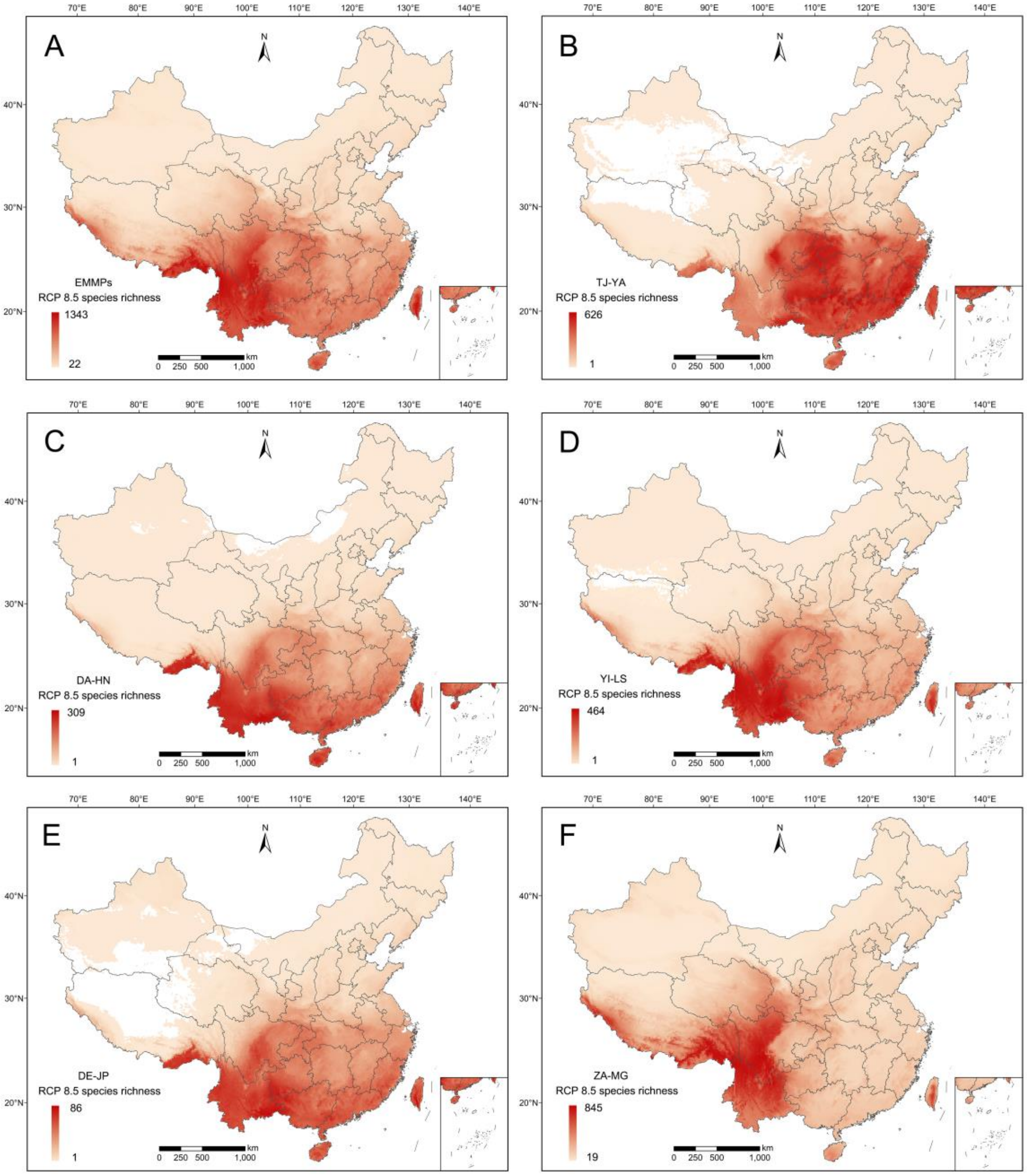

Figure S33. Spatial distribution patterns of species richness for different plant categories based on the results of MaxEnt. Time periods: future (2070). Emission scenarios: Representative concentration pathway (RCP) 8.5. (A) Endemic and threatened EMMPs of 24 ethnic minorities. (B) Endemic and threatened EMMPs of TJ-YA clade. (C) Endemic and threatened EMMPs of DA-HN clade. (D) Endemic and threatened EMMPs of YI-LS clade. (E) Endemic and threatened EMMPs of DE-JP clade. (F) Endemic and threatened EMMPs of ZA-MG clade.

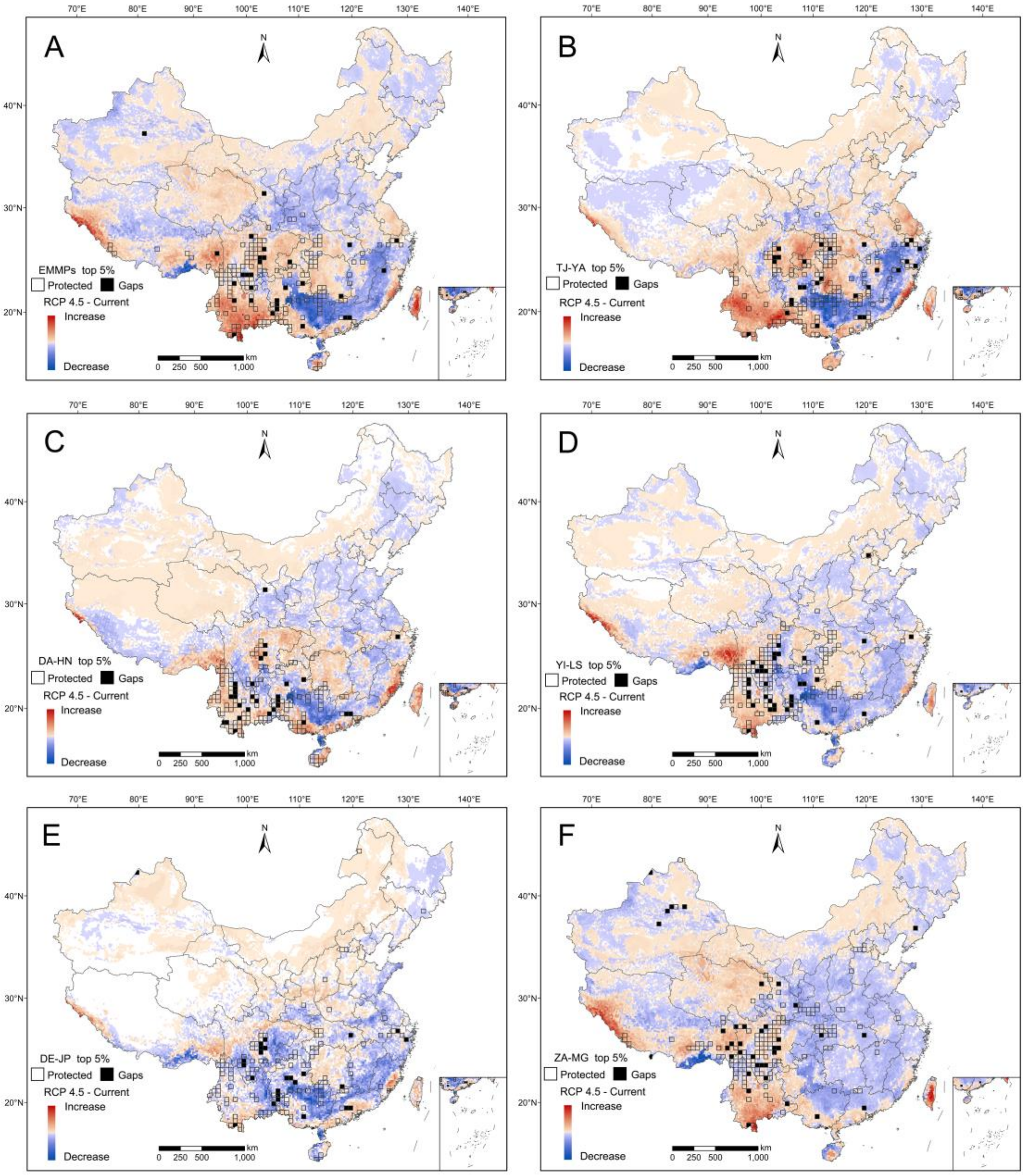

Figure S34. Species richness change for different plant categories by 2070 under the emission scenarios of representative concentration pathway [RCP] 4.5. The white and black grids represent the top 5% hotspot grids covered or uncovered by national and provincial nature reserves, respectively. (A) Endemic and threatened EMMPs of 24 ethnic minorities. (B) Endemic and threatened EMMPs of TJ-YA clade. (C) Endemic and threatened EMMPs of DA-HN clade. (D) Endemic and threatened EMMPs of YI-LS clade. (E) Endemic and threatened EMMPs of DE-JP clade. (F) Endemic and threatened EMMPs of ZA-MG clade.

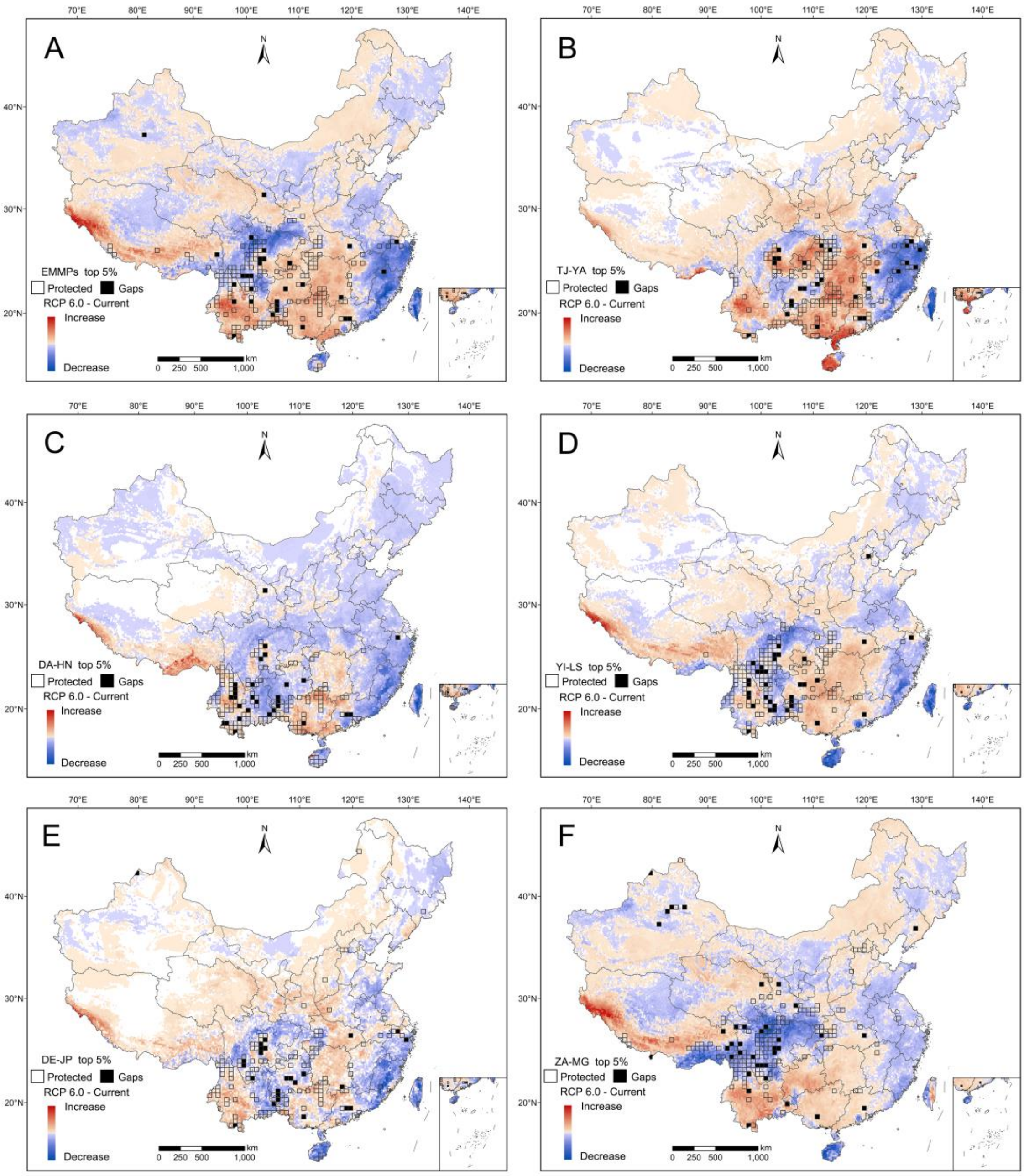

Figure S35. Species richness change for different plant categories by 2070 under the emission scenarios of representative concentration pathway [RCP] 6.0. The white and black grids represent the top 5% hotspot grids covered or uncovered by national and provincial nature reserves, respectively. (A) Endemic and threatened EMMPs of 24 ethnic minorities. (B) Endemic and threatened EMMPs of TJ-YA clade. (C) Endemic and threatened EMMPs of DA-HN clade. (D) Endemic and threatened EMMPs of YI-LS clade. (E) Endemic and threatened EMMPs of DE-JP clade. (F) Endemic and threatened EMMPs of ZA-MG clade.

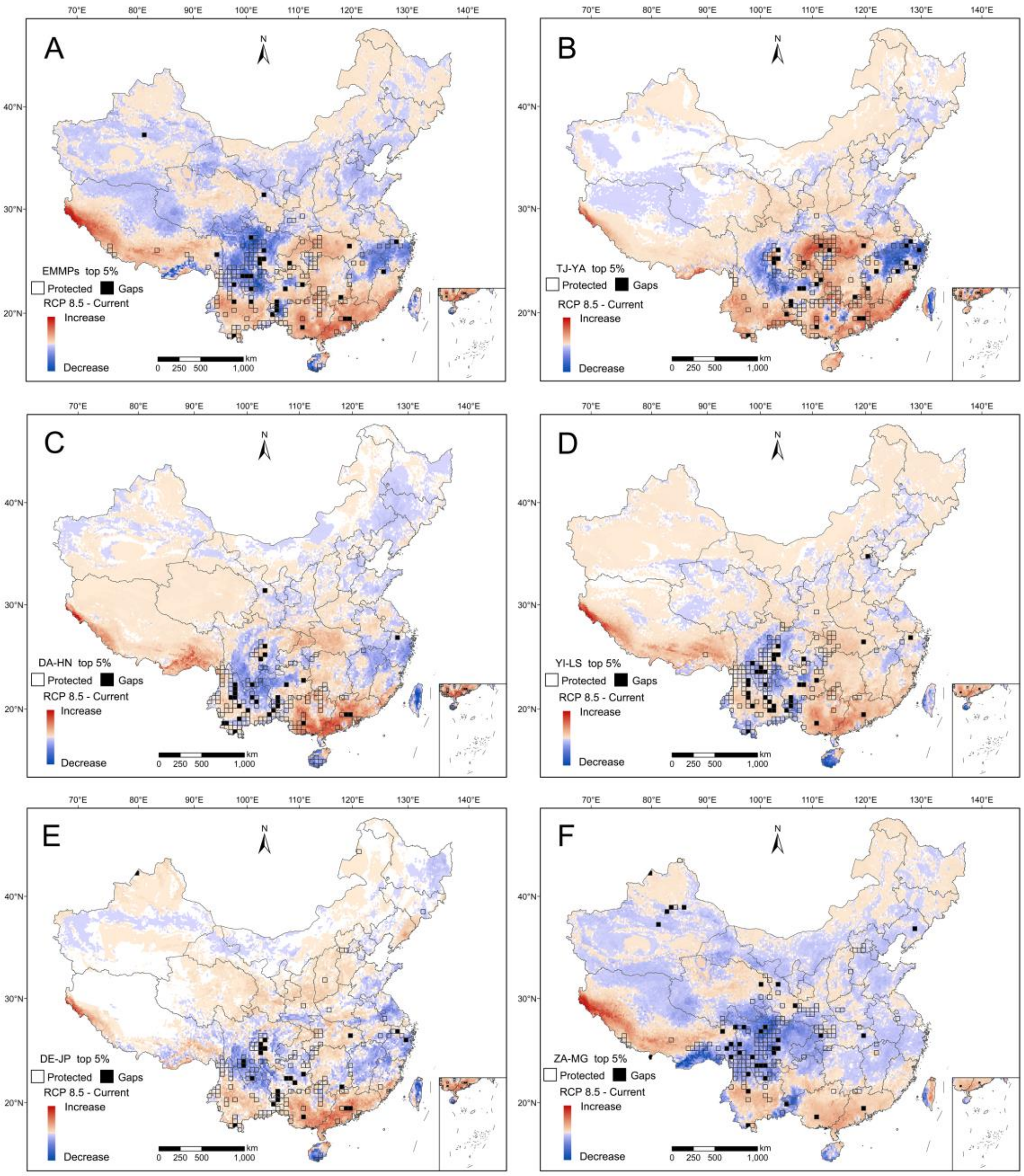

Figure S36. Species richness change for different plant categories by 2070 under the emission scenarios of representative concentration pathway [RCP] 8.5. The white and black grids represent the top 5% hotspot grids covered or uncovered by national and provincial nature reserves, respectively. (A) Endemic and threatened EMMPs of 24 ethnic minorities. (B) Endemic and threatened EMMPs of TJ-YA clade. (C) Endemic and threatened EMMPs of DA-HN clade. (D) Endemic and threatened EMMPs of YI-LS clade. (E) Endemic and threatened EMMPs of DE-JP clade. (F) Endemic and threatened EMMPs of ZA-MG clade.

Table S3. Environmental variables used in the species distribution modeling process.

Variable code	Variable type	Unit	Select
Bio1	Annual mean temperature	°C	Yes
Bio2	Mean diurnal range (mean of monthly max. and min. temp.)	°C	Yes
Bio3	Isothermally ((Bio2/Bio7) × 100)	–	Yes
Bio4	Temperature seasonality (standard deviation × 100)	(coeff. of variation °C)	Yes
Bio5	Maximum temperature of warmest month	°C	
Bio6	Minimum temperature of coldest month	°C	
Bio7	Temperature annual range (Bio5–Bio6)	°C	
Bio8	Mean temperature of wettest quarter	°C	
Bio9	Mean temperature of driest quarter	°C	
Bio10	Mean temperature of warmest quarter	°C	
Bio11	Mean temperature of coldest quarter	°C	
Bio12	Annual precipitation	mm	Yes
Bio13	Precipitation of wettest period	mm	
Bio14	Precipitation of driest period	mm	Yes
Bio15	Precipitation seasonality (CV)	(coeff. of variation; percent	Yes
Bio16	Precipitation of wettest quarter	mm	
Bio17	Precipitation of driest quarter	mm	
Bio18	Precipitation of warmest quarter	mm	Yes
Bio19	Precipitation of coldest quarter	mm	
Elevation		m	Yes

Table S4 Numbers and proportions of grids in hotspots and nature reserves.

Threshold	Type	EMMPs	TU-YA	DA-HN	YI-LS	DE-JP	ZA-MG
Top 5%	Final hotspots grids	179	158	150	162	142	179
	Protected by NNRs	101 (56.42%)	95 (60.13%)	80 (53.33%)	90 (55.56%)	77 (54.23%)	97 (54.19%)
	Unprotected by NNRs	78 (43.58%)	63 (39.87%)	70 (46.67%)	72 (44.44%)	65 (45.77%)	82 (45.81%)
	Protected by PNRs	96 (53.63%)	87 (55.06%)	77 (51.33%)	79 (48.77%)	83 (58.45%)	87 (48.60%)
	Unprotected by PNRs	83 (46.37%)	71 (44.94%)	73 (48.67%)	83 (51.23%)	59 (41.55%)	92 (51.40%)
	Protected by NNRs-PNRs	147 (82.12%)	129 (81.65%)	117 (78.00%)	127 (78.40%)	116 (81.69%)	141 (78.77%)
	Unprotected by NNRs-PNRs	32 (17.88%)	29 (18.35%)	33 (22.00%)	35 (21.60%)	26 (18.31%)	38 (21.23%)
Top 10%	Final hotspots grids	358	316	300	325	285	358
	Protected by NNRs	196 (54.75%)	177 (56.01%)	150 (50.00%)	166 (51.08%)	149 (52.28%)	196 (54.75%)
	Unprotected by NNRs	162 (45.25%)	139 (43.99%)	150 (50.00%)	159 (48.92%)	136 (47.72%)	162 (45.25%)
	Protected by PNRs	190 (53.07%)	176 (55.70%)	152 (50.67%)	161 (49.54%)	157 (55.09%)	174 (48.60%)
	Unprotected by PNRs	168 (46.93%)	140 (44.30%)	148 (49.33%)	164 (50.46%)	128 (44.91%)	184 (51.40%)
	Protected by NNRs-PNRs	291 (81.28%)	253 (80.06%)	226 (75.33%)	242 (74.46%)	223 (78.25%)	275 (76.82%)
	Unprotected by NNRs-PNRs	67 (18.72%)	63 (19.94%)	74 (24.67%)	83 (25.54%)	62 (21.75%)	83 (23.18%)
Top 17%	Final hotspots grids	609	537	509	553	484	607
	Protected by NNRs	300 (49.26%)	249 (46.37%)	231 (45.38%)	259 (46.84%)	226 (46.69%)	316 (52.06%)
	Unprotected by NNRs	309 (50.74%)	288 (53.63%)	278 (54.62%)	294 (53.16%)	258 (53.31%)	291 (47.94%)
	Protected by PNRs	308 (50.57%)	288 (53.63%)	262 (51.47%)	282 (50.99%)	256 (52.89%)	306 (50.41%)
	Unprotected by PNRs	301 (49.43%)	249 (46.37%)	247 (48.53%)	271 (49.01%)	228 (47.11%)	301 (49.59%)
	Protected by NNRs-PNRs	458 (75.21%)	397 (73.93%)	372 (73.08%)	405 (73.24%)	360 (74.38%)	464 (76.44%)
	Unprotected by NNRs-PNRs	151 (24.79%)	140 (26.07%)	137 (26.92%)	148 (26.76%)	124 (25.62%)	143 (23.56%)
Top 30%	Final hotspots grids	1075	948	899	975	854	1071
	Protected by NNRs	447 (41.58%)	366 (38.61%)	348 (38.71%)	386 (39.59%)	343 (40.16%)	496 (46.31%)
	Unprotected by NNRs	628 (58.42%)	582 (61.39%)	551 (61.29%)	589 (60.41%)	511 (59.84%)	575 (53.69%)
	Protected by PNRs	516 (48.00%)	483 (50.95%)	437 (48.61%)	493 (50.56%)	436 (51.05%)	501 (46.78%)
	Unprotected by PNRs	559 (52.00%)	465 (49.05%)	462 (51.39%)	482 (49.44%)	418 (48.95%)	570 (53.22%)
	Protected by NNRs-PNRs	741 (68.93%)	650 (68.57%)	606 (67.41%)	673 (69.03%)	594 (69.56%)	762 (71.15%)
	Unprotected by NNRs-PNRs	334 (31.07%)	298 (31.43%)	293 (32.59%)	302 (30.97%)	260 (30.44%)	309 (28.85%)

Table S5 Numbers and proportions of species in hotspots and nature reserves.

Type	Threshold	Hotspots Grids	protected by NNRs	protected by PNRs	protected by NNRs-PNRs	unprotected by NNRs	unprotected by PNRs	unprotected by NNRs-PNRs
All	Top 5%	5652 (97.11%)	5295 (90.98%)	5213 (89.57%)	5416 (93.06%)	5400 (92.78%)	5453 (93.69%)	5066 (87.04%)
	Top 10%	5729 (98.44%)	5466 (93.92%)	5467 (93.93%)	5625 (96.65%)	5568 (95.67%)	5644 (96.98%)	5259 (90.36%)
	Top 17%	5787 (99.43%)	5655 (97.16%)	5592 (96.08%)	5723 (98.33%)	5663 (97.30%)	5733 (98.51%)	5507 (94.62%)
	Top 30%	5814 (99.90%)	5733 (98.51%)	5685 (97.68%)	5774 (99.21%)	5735 (98.54%)	5782 (99.35%)	5639 (96.89%)
Endemic	Top 5%	1927 (97.08%)	1869 (94.16%)	1828 (92.09%)	1898 (95.62%)	1811 (91.23%)	1845 (92.95%)	1654 (83.32%)
	Top 10%	1962 (98.84%)	1920 (96.73%)	1874 (94.41%)	1945 (97.98%)	1887 (95.06%)	1922 (96.83%)	1741 (87.71%)
	Top 17%	1977 (99.60%)	1939 (97.68%)	1903 (95.87%)	1958 (98.64%)	1924 (96.93%)	1947 (98.09%)	1842 (92.80%)
	Top 30%	1985 (100.00%)	1957 (98.59%)	1929 (97.18%)	1972 (99.35%)	1949 (98.19%)	1968 (99.14%)	1889 (95.16%)
Threatened	Top 5%	296 (96.73%)	276 (90.20%)	267 (87.25%)	284 (92.81%)	264 (86.27%)	280 (91.50%)	239 (78.10%)
	Top 10%	300 (98.04%)	279 (91.18%)	279 (91.18%)	292 (95.42%)	286 (93.46%)	288 (94.12%)	258 (84.31%)
	Top 17%	305 (99.67%)	288 (94.12%)	284 (92.81%)	297 (97.06%)	291 (95.10%)	295 (96.41%)	275 (89.87%)
	Top 30%	306 (100.00%)	293 (95.75%)	289 (94.44%)	302 (98.69%)	293 (95.75%)	298 (97.39%)	279 (91.18%)
National key protected wild plants	Top 5%	217 (97.31%)	199 (89.24%)	200 (89.69%)	205 (91.93%)	204 (91.48%)	210 (94.17%)	188 (84.30%)
	Top 10%	221 (99.10%)	205 (91.93%)	210 (94.17%)	214 (95.96%)	214 (95.96%)	214 (95.96%)	199 (89.24%)
	Top 17%	223 (100%)	212 (95.07%)	214 (95.96%)	217 (97.31%)	217 (97.31%)	217 (97.31%)	211 (94.62%)
	Top 30%	223 (100%)	214 (95.96%)	215 (96.41%)	219 (98.21%)	220 (98.65%)	220 (98.65%)	216 (96.86%)

Table S6 Number of species with potential change in distribution area.

Climate scenarios	Increase	Decrease	Remain unaltered
RCP 2.6	1058（49.62%）	1072(50.28%)	2(0.09%)
RCP 4.5	1103（51.74%）	1029(48.26%)	0.00%
RCP 6.0	1122（52.63%）	1010(47.37%)	0.00%
RCP 8.5	1134（53.19）	997(46.76)	1(0.05%)

Table S7 Number of grids with potential change in distribution area.

Climate scenarios	Increase	Decrease	Remain unaltered
RCP 2.6	20074(57.99%)	13029(37.64%)	1515(4.38%)
RCP 4.5	19573(56.54%)	13409(38.73%)	1636(4.73%)
RCP 6.0	19675(56.83%)	13447(38.84%)	1496(4.32%)
RCP 8.5	19175(55.39%)	14012(40.48)	1431(4.13)
